# Structure-enhanced deep learning accelerates aptamer selection for small molecule families like steroids

**DOI:** 10.1093/bib/bbaf680

**Published:** 2025-12-18

**Authors:** Zibin Zhao, Haosi Lin, Hoi Ying Lau, Hao Chen, I-Ming Hsing

**Affiliations:** Department of Chemical and Biological Engineering, The Hong Kong University of Science and Technology, Clear Water Bay, Kowloon, Hong Kong SAR 999077, China; Department of Chemical and Biological Engineering, The Hong Kong University of Science and Technology, Clear Water Bay, Kowloon, Hong Kong SAR 999077, China; Department of Chemical and Biological Engineering, The Hong Kong University of Science and Technology, Clear Water Bay, Kowloon, Hong Kong SAR 999077, China; Department of Chemical and Biological Engineering, The Hong Kong University of Science and Technology, Clear Water Bay, Kowloon, Hong Kong SAR 999077, China; Department of Computer Science and Engineering, The Hong Kong University of Science and Technology, Clear Water Bay, Kowloon, Hong Kong SAR 999077, China; Department of Chemical and Biological Engineering, The Hong Kong University of Science and Technology, Clear Water Bay, Kowloon, Hong Kong SAR 999077, China

**Keywords:** deep learning, SELEX, steroid aptamers, small molecules, variational autoencoder, isothermal titration calorimetry

## Abstract

The efficient discovery of high-affinity small-molecule aptamers via the Systematic Evolution of Ligands by EXponential enrichment (SELEX) is often constrained by challenges in navigating vast sequence spaces and rationally designing initial libraries. In this study, we introduce Deep Learning-assisted SELEX (DL-SELEX), a novel two-step framework that employs variational autoencoders (VAEs) to accelerate and refine small-molecule aptamer selection. This approach is the first to integrate deep learning to design initial aptamer libraries, marking a significant advancement in SELEX workflows. DL-SELEX leverages shared structural features within molecular families (e.g. steroids) to guide aptamer design: AptaVAE, the first VAE enriched with transfer learning from foundation models, generates tailored initial pools, whereas AptaClux, a second VAE, identifies high-performance candidates from SELEX-derived next-generation sequencing (NGS) data by capturing consensus structural features. The application of DL-SELEX to hydrocortisone (CS) and testosterone (TES) yielded aptamers with up to 450-fold higher affinity than previously reported aptamers and reduced SELEX iterations by up to 80%. Critically, these results demonstrate that structural commonalities can be used to train deep learning models to design aptamers for structurally similar targets. DL-SELEX provides an effective, generalizable strategy to streamline aptamer discovery and enables *de novo* design of high-affinity aptamers for challenging small molecules.

## Graphical Abstract

**Graphical Abstract:**
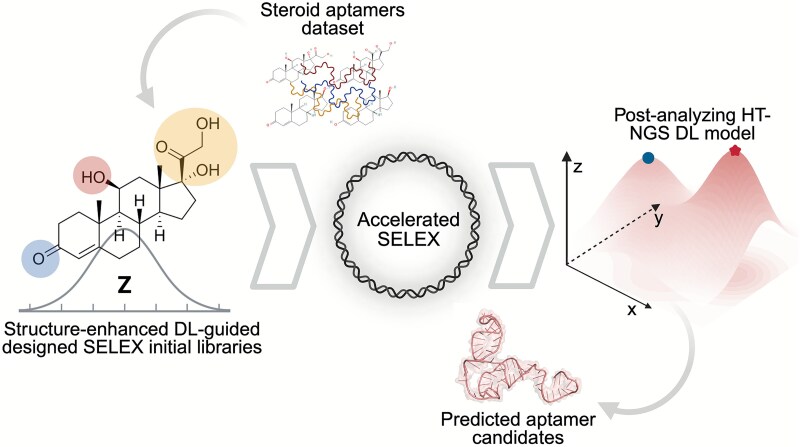

## Introduction

Aptamers, which are short single-stranded oligonucleotides (ssDNA or ssRNA), are highly regarded for their exceptional affinity and specificity. These properties make them valuable tools in applications such as biosensors, cancer therapy, and neuroscience [1–3]. Compared to common bioreceptors, such as antibodies, aptamers offer several advantages, including lower production costs, greater batch stability, and ease of chemical synthesis [4]. Despite these benefits, the discovery and selection of aptamers remains a time-consuming process that relies heavily on *in vitro* methods such as SELEX [5].

SELEX is a repetitive process in which a randomized pool of ssDNA or ssRNA sequences is subjected to selection rounds for various target compounds, including small molecules, proteins, and cells. After 8–30 rounds, the enriched pool is sequenced via next-generation sequencing (NGS) and screened for high-affinity candidates, followed by labor-intensive experimental validation [6]. Although various SELEX modifications (e.g. NA-SELEX [7], nonfouling hydrogel-based SELEX [8] and motif-SELEX [9]) have improved speed and specificity under certain conditions, significant challenges persist, especially for small molecules, because of their limited moieties for binding and handling [10]. The experimental process often spans weeks to months, and the randomized initial library serves as a “black box” with sequence information emerging only at the final stages [11]. Furthermore, it is impractical for any initial library to include all potential high-performance sequences, implying that some optimal candidates may never enter the selection process [12]. Although the above-mentioned issue of SELEX persists, many researchers still prefer to perform customized SELEX under specific experimental conditions and usage scenarios for optimal performance of aptamers in their applications [10]. These limitations and demands highlight the need for strategies that accelerate selection while improving candidate quality.

Recent advances in artificial intelligence (AI) have created new opportunities to address these challenges, particularly using machine learning (ML). AI applications in aptamer research can be broadly divided into two domains: generation and classification. Generative models such as MLPD [13], HFNAP [14], RaptGen [15], RBM [16], ProBound [17], DeepBind [18], and AptaDiff [19] have been used to analyze sequencing data, score sequence performance, predict binding sites, and generate novel aptamer candidates with enhanced affinity. Classification models, such as AptaNet [20] and AptaTrans [21], predict the binding activity against specific targets. While promising, these approaches mainly focus on postselection processing or target-specific predictions from SELEX-selected pools, often lacking generalizability across diverse targets or sufficient confidence in classification models because of the limited available datasets. Moreover, current workflows barely address inefficiencies in library design, where a randomized initial pool may hinder the selection of aptamers for poorly understood or challenging small-molecule targets.

In this study, we aimed to rationally design initial libraries and intelligently predict optimal sequences from NGS data using deep learning approaches for small-molecule aptamer selection, using the steroid family as a representative target group. Steroid molecules have been proven to be critical biomarkers for stress monitoring (e.g. cortisol) and sports performance (e.g. testosterone, cholesterol) [22]. However, aptamer selection for steroids presents a unique challenge because of its hydrophobicity and conserved structural features, including a four-ring carbon backbone [23]. Recent advancements by Yang *et al.* [24] have shown that functional group relationships can enhance the probability of successful selection, especially for challenging small-molecule targets, such as steroids. Many researchers have attempted to manually design initial libraries based on known structures (e.g. G-quadruplexes or known stem duplexes) to accelerate the aptamer selection process [25, 26]. These findings suggest that guided initial libraries can be useful in shortening the selection process, as the structural features shared among steroid molecules can guide the design of aptamers with greater specificity and affinity. More importantly, these shared characteristics may provide a foundational assumption for training deep learning models, enabling the design of aptamers for other as-yet-unknown steroid molecules with only minor functional group differences.

To address these challenges, we propose a novel deep learning-guided approach, DL-SELEX, for designing initial libraries for streamline aptamer selection. By incorporating structural knowledge from the outset, our method integrates target-specific information, thereby accelerating the selection process to identify high-performance aptamers. This approach employed a two-step structure-enhanced methodology. The first component, AptaVAE, utilizes a modified VAE to design a guided initial library tailored for steroid molecules, leveraging the shared structural features within the steroid family. The second component, AptaClux, extracts consensus structural features from the NGS data to identify superior aptamer candidates. By exploiting the functional relationships between steroid molecules and their aptamers, our approach lays the foundation for a novel, functional group-informed perspective on aptamer-ligand interactions. This strategy could enhance the ML generalizability in aptamer discovery across diverse molecular families, with potential applications in diagnostics and therapeutics.

## Materials and methods

### AptaVAE: generating structural-based guided initial library

#### Data preparation and encodings

Raw aptamer sequences of eight steroid family molecules were collected from literature and online resources [23, 27–34] (Table S1). The chemical structures of all the steroids are shown in Fig. S1. The raw steroid aptamer sequences, along with their corresponding relative scores, were initially preprocessed to eliminate redundant or abnormal sequences, such as those containing regions that do not bind. These sequences were collected from various published journals and each employed different primers. To standardize the data and eliminate primer effects, aptamer sequences were truncated to the minimum length required to maintain their global secondary structure (see Fig. S2 for details). Processed truncated sequences, along with their primer-refined secondary structures and relative scores, were prepared for encoding. Nucleotide sequences and their secondary structures in the dot-bracket notation are one-hot encoded. To better capture the structural relationship between sequences and targets, a 3D tensor was constructed for each aptamer-target pair, consisting of the target distance matrix, sequence secondary structure one-hot encoding, and target adjacency matrix. The order of the 3D tensor was inspired and designed by a “sandwich” to reflect the binding mode of an aptamer and its target in a three-dimensional space (details in Fig. S2).

#### Pretrained embeddings and the modified attention mechanism

To enhance reliability and broaden the pretrained knowledge of our model, we incorporated pretrained embeddings for sequences and targets using a large language model (LLM) of bidirectional encoder representations from Transformer (BERT) models. DNA sequences were embedded with 6-mer DNABERT [35], and the target SMILES was embedded with CHEMBERT [36]. These embeddings were further processed through an attention mechanism to effectively capture their interactions. Inspired by the co-attention mechanism, we designed a model to extract aptamer-target interactions. Target embedding served as the query input, whereas aptamer sequences acted as key and value inputs. This design was analogous to identifying the sequence (key and value) that would exhibit strong interactions or binding potential with a given target (query). The resulting co-attention outputs were concatenated back into the model by integrating the pretrained knowledge into the overall framework. We modified the attention calculation with the query (target) being reiterated independently, aiming to strengthen the target-specific information that attention might capture. The additional query term was scaled back by dividing it by its own square root function. Details of the attention formalization and workflow are shown in Note S10 and Fig. S2, respectively. Padding and masking were applied, accounting for the various lengths of the targets. The modified attention calculation, designed to optimize aptamer-target interaction modeling, was


(1) \begin{equation*} Modified\ attention\left(Q,K,V\right)= softmax\left(\frac{Q{K}^T}{\sqrt{d_K}}\right)V+\frac{Q}{\sqrt{d_Q}} \end{equation*}

#### Model architecture and loss calculation

We employ a modified VAE with an integrated attention mechanism. The model architecture is shown in Fig. S3. To account for the varying representations across concatenated segments, a composite loss function was applied as follows:


(2) \begin{align*} \displaystyle model\ loss =&\ se{quence}_{loss}+ targe{t}_{loss}+ clas{s}_{loss}+2\ast scor{e}_{loss}+\nonumber\\{}& \ attentio{n}_{loss}+ matri{x}_{loss}+K{L}_{Divergence} \end{align*}

(3) \begin{equation*} matri{x}_{loss}=\alpha \ast distanc{e}_{loss}+\beta \ast structur{e}_{loss}+\left(1-\alpha -\beta \right)\ast adjacenc{y}_{loss} \end{equation*}

Sequence loss was calculated as the difference between the reconstructed sequence tensor and the original sequence tensor. The target loss was defined as the reconstruction loss for the target’s Morgan fingerprint, similar to the class loss. The score loss, which is critical for evaluating the quality of the generated sequences, was weighted more heavily by doubling the contribution. Matrix loss incorporates three structural components: the distance matrix, sequence structure matrix, and adjacency matrix, each weighted according to its importance. The weights of the hyperparameters were optimized by optimizing the validation loss using α = 0.3 and β = 0.4, giving greater emphasis to sequence structural features compared to the target matrices. Model loss was minimized with the best validation loss. We employed a binary cross-entropy loss function for the sequence, target, class, structure, and adjacency losses. The mean squared error loss was used for score, attention, and distance losses. Additionally, the Kullback–Leibler (KL) divergence loss was applied as


(4) \begin{equation*} {D}_{KL}\left(p\parallel q\right)={\mathbb{E}}_{X\sim P}\left[\frac{\log p(x)}{q(x)}\right]={\mathbb{E}}_{X\sim p}\left[\log p(x)-\log q(x)\right] \end{equation*}

The binary cross entropy loss can be denoted as:


(5) \begin{equation*} {l}_n=-{w}_n\left[{y}_n\cdotp \log{x}_n+\left(1-{y}_n\right)\cdotp \log \left(1-{x}_n\right)\right] \end{equation*}

Mean squared error loss:


(6) \begin{equation*} \kern0.5em \ell \left(x,y\right)=L={\left\{{l}_1,\dots, {l}_N\right\}}^T,{l}_n={\left({x}_n-{y}_n\right)}^2 \end{equation*}

We also investigated the impact of different latent-space dimensions on the VAE during parameter fine-tuning. Detailed comparisons of the latent-space dimension results are provided in Figs S4–S9 and Table S2.

#### Sequence decoding and sampling

New steroid affinity aptamer sequences were randomly sampled from the trained 256-dimensional latent space AptaVAE model and stabilized with batch-generated analysis (BGA). This process involves determining the optimal batch size and number of batches to ensure consistent results. Specifically, the BGA assessed the latent-space stability by evaluating the mean, standard deviation, and proportion of sequences exceeding the score threshold (0.9). These parameters were optimized to achieve a steady state, with the resulting high-scoring sequences used for the initial library design (the BGA results are shown in Fig. S10). The BGA outputs were further grouped and sorted by class and sequence scores across the eight steroid classes. The decoded sequences were then formatted in FASTA for multiple sequence alignment (MSA) analysis.

#### Multiple sequence alignment and guided SELEX initial library generation

Multiple Alignment using the Fast Fourier Transform (MAFFT) program was used for MSA with high accuracy and stability. To avoid an ad hoc choice, we also compared the MSA results across various methods (Fig. S11). The guided initial libraries for all eight steroid targets are listed in Table S3.

### AptaClux: summarizing key consensus aptamer candidates from the HT-NGS result

#### Data collection and pre-processing

NGS data were experimentally obtained from rounds 3, 5, and 7 of AptaVAE-guided SELEX for CS and TES. Sequencing was performed by Azenta Life Science, and the raw data were analyzed using AptaSuite [37] and AptaClux. Similar to the sequences processed in AptaVAE, NGS-derived sequences were encoded with one-hot representations for both nucleotides and their predicted secondary structures by NUPACK (nupack.org).

#### Model architecture and loss

A variational autoencoder (VAE) was employed to identify shared features from the NGS data. Similar to AptaVAE, the AptaClux model comprises an encoder and decoder; however, it processes only a single type of data and output. The detailed encoding and model architectures are shown in Fig. S12. The overall loss can be expressed as


(7) \begin{equation*} model\ loss= sequenc{e}_{loss}+ structur{e}_{loss}+K{L}_{Divergence} \end{equation*}

Sequence loss, structure loss, and KL divergence were calculated as described in AptaVAE. The model loss was minimized and the hyperparameters were optimized with the lowest validation loss.

#### Conventional next-generation sequencing data post-processing

Before evaluating the ability of AptaClux to generate new sequences, we applied conventional NGS data post-processing methods from published literature [24] as a benchmark for comparison. Raw NGS data obtained from Azenta Life Science were processed using the AptaSuite [37] platform. Different rounds of NGS data were obtained from the SELEX experiments for CS and TES. For each target, round, forward, and backward reads were analyzed to extract aptamer candidates as the largest cluster consensus sequence and the whole library consensus sequence. This selection approach was based on a previously designed library of hydrocortisone SELEX experiments, which demonstrated that only these criteria consistently produced high-affinity aptamers for the selected targets. Additionally, we tested other selection criteria, including the top frequency sequences across the dataset, top enrichment sequences from two rounds of NGS, and top frequency sequences from the largest cluster (see the results in Figs S13–S14 and Table S4).

#### AptaClux latent sampling

The trained latent space was used to generate new sequences. The encoded samples were passed through the latent space, and two different clustering methods were applied to the latent variables derived from the trained mean and log variance. A Gaussian kernel density estimation with a bandwidth of 0.5 was used to identify high-density regions. The top 1% density of sequences from these regions was extracted and aligned using MSA, as previously described, to derive a final high-density consensus aptamer. Second, K-means clustering with 10 fixed-seed clusters was performed, and the cluster center sequences were decoded. These sequences were aligned to generate a final cluster-consensus aptamer for further experimental validation. For each cluster, sequence logo maps were generated and are presented in Fig. S15.

### Dynamic simulation and docking

The complete molecular dynamics (MDs) workflow of this study is shown in Fig. S16. A high-accuracy 3D model of the aptamers was obtained using trRosettaRNA [38]. A full MDs pipeline was constructed using GROMACS [39]. The AMBER99 force field and TIP3P water model were employed, with the ion conditions set at 0.005 M MgCl and 0.1 M NaCl. Energy minimization was followed by stabilization using a constant number of particles, volume, temperature, and pressure ensembles (NVT and NPT) at 1 atm and 25°C. The production MD simulation was performed for 300 ns. The resulting aptamer structures were docked with their corresponding target molecules using AutoDock Vina [40, 41].

### Standardized and simplified selection protocol

All aptamer candidates were obtained from Integrated DNA Technologies IDT by HPLC purification. The DL-SELEX workflow incorporates a simplified and standardized approach to aptamer selection, utilizing iterative rounds of qPCR, annealing, selection, and asPCR for library regeneration. The detailed workflow can be found in Notes S1.

## Results

**Figure 1:**
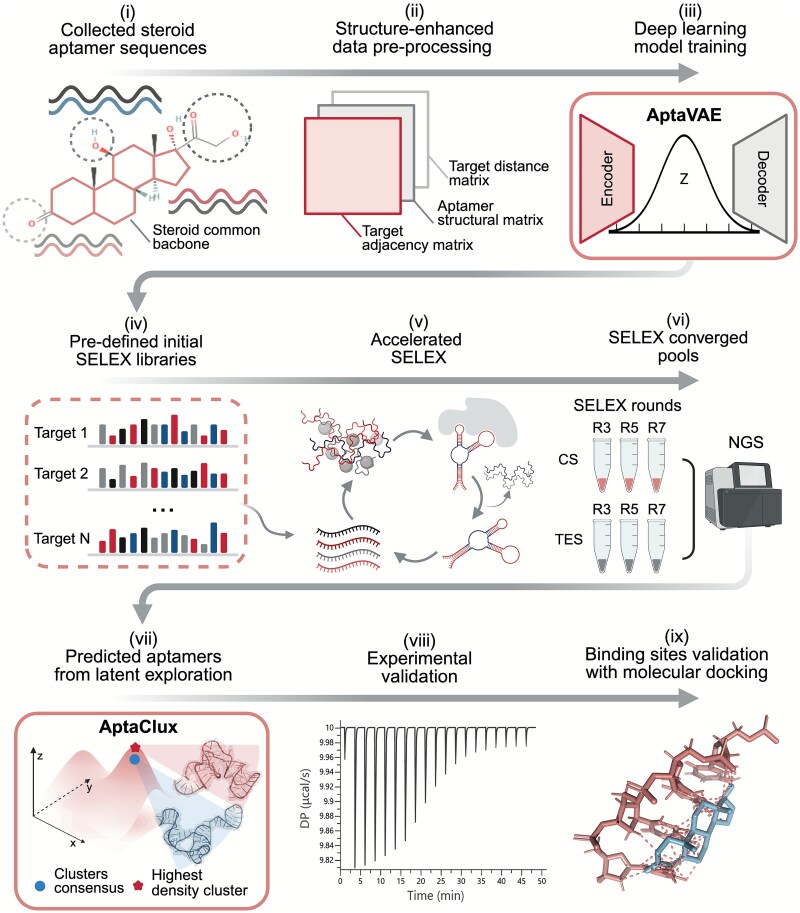
Overview of the DL-SELEX, a two-step deep learning-guided SELEX framework.

The DL-SELEX workflow was designed to accelerate SELEX by efficiently generating the top aptamer candidates. As shown in Fig. 1, DL-SELEX integrates two VAEs into two modules. The first module, AptaVAE (Fig. 1, top block), was trained on the collected steroid aptamer-target pairs ([Fig. 1 (i)]. This involved structure-enhanced data pre-processing ([Fig. 1 (ii)] feeding into the AptaVAE model [Fig. 1 (iii)], which generates pre-defined libraries tailored for steroid molecules [Fig. 1 (iv)]. For each trained steroid target, AptaVAE created a unique library [Fig. 1 (v)], allowing a simplified, standardized SELEX experimental workflow (Note S1 [42]). The second module, AptaClux (Fig. 1, bottom block), summarizes NGS data from enriched SELEX pools in the early, middle, and late rounds for hydrocortisone (CS) and testosterone (TES) [Fig. 1 (vi)]. AptaClux clusters NGS data with structural information to generate aptamer candidates based on consensus secondary structures that are favorable for binding [Fig. 1 (vii)]. The candidates underwent experimental validation via isothermal titration calorimetry (ITC) [Fig. 1 (viii)], and the selected aptamers were further analyzed using MDs and docking simulations [Fig. 1 (ix)]. The first role of the VAE is to generate guided initial libraries, thereby improving sequence quality by reducing randomness. Although trained on shared structural backbones within the steroid group, specificity limitations were experimentally addressed through counterselection. The second VAE, AptaClux, provided robust post-selection analysis and further streamlined the aptamer discovery process. By simplifying the workflow complexity, DL-SELEX efficiently accelerates the development of small-molecule aptamers for diagnostic and therapeutic applications.

**Figure 2:**
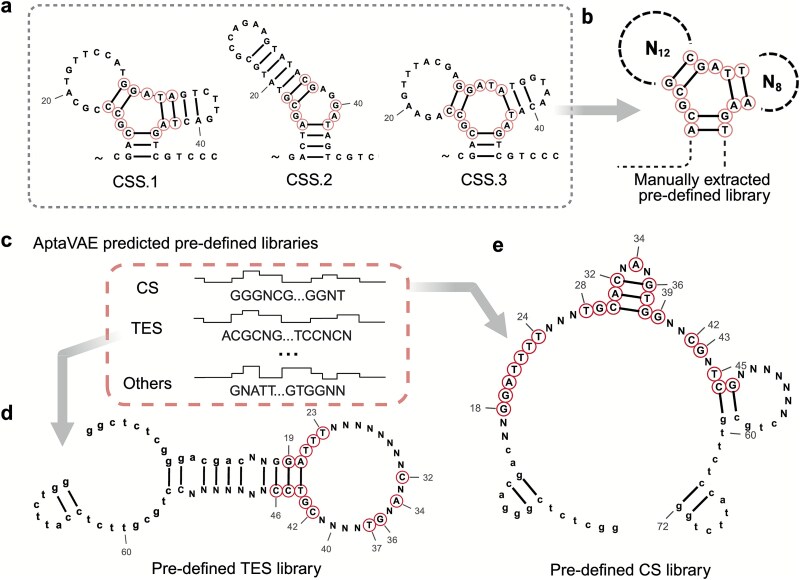
Structure-guided design of pre-defined libraries using manual extraction and AptaVAE prediction for CS and TES. (a) Secondary structure of three published cortisol aptamers (CSS.1, CSS.2, and CSS.3), adapted from Yang *et al.* [23], and (b) represent the extracted confined bases for the manual design initial library for the hydrocortisone (CS) SELEX experiment as a proof-of-concept. The letters “A,” “G,” “C,” and “T” denote the four DNA bases, while “N” signifies any DNA base. The notation “N_12_” refers to a sequence of 12 random bases used to construct the loop. Numbers next to the bases indicate their position, starting from the 5 prime end of the sequence. (c) AptaVAE-generated pre-defined libraries for trained steroid molecules. The predicted pre-defined libraries for (d) testosterone (TES), and (e) hydrocortisone (CS) were taken as the case studies in this work, with red circles indicating the confined bases identified by the AptaVAE model. Lowercase letters represent the primer region used in this study.

To evaluate the impact of a structured initial library, we used hydrocortisone (CS) as a proof-of-concept and extracted shared structural information from published CS aptamers [23] (Fig. 2a). Informed by previous reports [31] that the CS binding site involves a hydrophobic group stabilized within the loop region, we designed a stem-loop structure for CS selection. We manually designed a fixed stem with varying loops (N12 or N8) (Fig. 2b) to enhance selection efficiency and accuracy. At round eight of SELEX, two aptamer candidates for CS were identified with apparent dissociation constants (K_D, apparent_) of 18.1 μM and 11.1 μM, as determined by fluorescent resonance energy transfer (FRET) (Fig. S18). In contrast, a previously reported CS aptamer required 15 rounds of SELEX to achieve a comparable affinity [30]. This demonstrates the feasibility and importance of optimizing the initial library design in SELEX, as demonstrated by our manual design library.

While this structure-based manual design reduces the number of SELEX rounds required to identify CS aptamers, scaling this approach to other targets faces significant challenges. For example, steroid aptamers lack a clear consensus motif tied to their shared four-fused-ring structure (Fig. S19). Moreover, the vast theoretical sequence space, ~10^25^ for a 40-nucleotide random region, far exceeds the practical limits of manually prepared libraries (~10^15^ sequences) and sequencing throughput (~10^6^ sequences). These constraints limit their ability to explore diverse sequences and identify high-affinity aptamers for a wider range of targets.

Given these challenges, a high-quality, pre-defined library is essential to reduce randomness, accelerate the SELEX process, and increase the likelihood of selecting aptamers with superior binding properties. We developed a deep learning approach to systematically analyze existing data, predict optimal library designs, and enhance the efficiency and generalizability of SELEX across diverse molecular families, thereby generating aptamers with high affinity and specificity. This approach comprises two VAE modules, AptaVAE and AptaClux. The AptaVAE module predicted pre-defined libraries for steroids (Fig. 2c, Table S3) by learning common steroid molecular structures. Libraries for hydrocortisone (CS) and TES were used in SELEX experiments (Fig. 2d and e). Accelerated SELEX for CS and TES was performed, followed by structural consensus summarization and candidate prediction using AptaClux based on HT-NGS data, identifying two aptamer candidates for CS and TES for validation.

**Figure 3:**
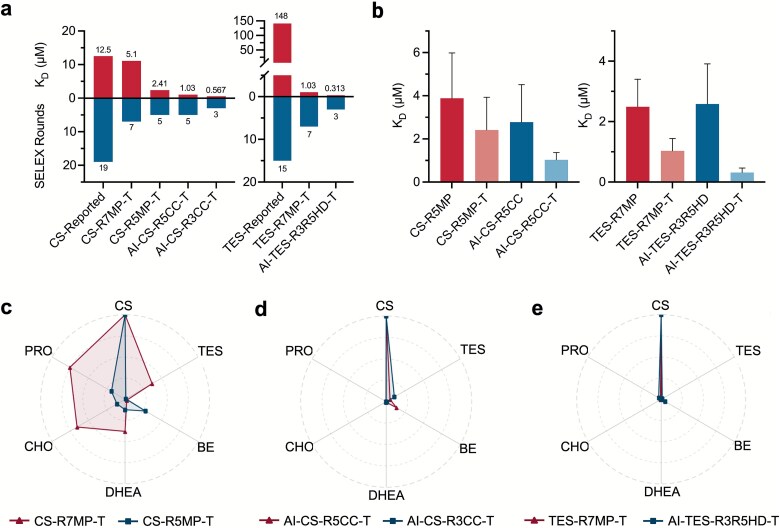
Experimental validation of DL-SELEX generated aptamers with isothermal titration calorimetry (ITC). (a) ITC results of comparing binding affinities and SELEX rounds of DL-SELEX generated aptamers to conventional analysis aptamers for CS and TES. The lower the dissociation constant (K_D_) value, the better the aptamer affinity. (b) Affinity comparison of truncated versus full-length aptamers to validate the truncation program used in this study. Error bars indicate the error values calculated by the machine-associated MicroCal PEAQ ITC analysis software with one-site fitting model. (c–e) Specificity analysis of CS against TES, BE, DHEA, CHO, and PRO, and of TES against CS, BE, DHEA, CHO, and PRO. Spikes indicate the normalized association constant (K_A_). Stronger spikes indicate higher affinity toward the respective targets. The precise affinity values expressed in K_D_ are in Figs S21–S65. The naming convention used in this figure and the rest of this study for aptamers are in Note S2. (c) Specificity analysis of the AptaVAE guided CS SELEX with conventional analysis generated aptamers. (d) Specificity analysis for DL-SELEX generated CS aptamers. (e) Specificity analysis for AptaVAE guided TES SELEX with conventional analysis (TES-R7MP-T) and DL-SELEX generated TES aptamers (AI-TES-R3R5HD-T).

Experimental validation is the most convincing approach for DL-SELEX-generated sequences. ITC is widely regarded as the gold standard for assessing aptamer quality [43]. ITC was performed to evaluate our aptamers against previously reported hydrocortisone (CS) [30] and TES [32] aptamers for benchmarking. The selection and characterization experiments were conducted identically (Fig. S1). The best DL-SELEX-generated CS aptamer (AI-CS-R3CC-T) showed a 20-fold affinity improvement and 80% reduction in SELEX rounds (Fig. 3a). Other CS aptamers demonstrated comparable affinity (K_D_ = 12.5 μM) and at least a 50% round reduction. The best TES aptamer (AI-TES-R3R5HD-T) achieved a K_D_ of 313 nM within three SELEX rounds, achieving a 450-fold affinity improvement and 80% fewer rounds. Other TES aptamers displayed at least a 100-fold affinity enhancement and a time reduction exceeding 50%. All CS aptamers, except CS-R7MP-T, showed at least a four-fold affinity improvement, whereas all TES aptamers improved by at least 20-fold. Interestingly, CS-R7MP-T did not outperform sequences from earlier selection rounds, despite the expectation that later SELEX rounds would yield higher-affinity aptamers [12]. This may indicate the accumulation of selection bias as affinity strands become enriched in the library (Note S3 [44]). We have also conducted orthogonal validation with our recent study on organic electrochemical transistors (OECTs) based aptamer investigation platform [45]. The cortisol aptamer R5MP-T, selected by the DL-SELEX workflow here, showed robust, temperature-tolerant induced-fit signals on the OECT platform, whereas several published cortisol aptamers exhibited little or no conformation change under comparable conditions. Notably, apparent affinity was consistent between immobilized (OECT) and solution ITC formats.

A primer refinement program truncated redundant sequences in the selected aptamers (Note S4 [25]), significantly enhancing their affinity, as confirmed by ITC (Fig. 3b). The truncated CS aptamers consistently exhibited enhanced affinity compared to the full-length versions. For TES aptamers, truncation produced at least two-fold affinity improvement, likely because key binding regions (bases A to T at positions 20 and 44, Fig. 2d) were less obstructed by steroid molecule tails (Fig. S20). The stem-loop structure from AptaVAE created an efficient binding pocket, enhancing TES selection efficiency compared to CS.

Specificity is another critical metric for evaluating the aptamer quality. During the selection for CS and TES, three other steroid molecules, cholesterol (CHO), dehydroepiandrosterone (DHEA), and CS/TES, were used for counter-selection. Additionally, beta-estradiol (BE) and progesterone (PRO), which are structurally similar noncounter targets, were included in the specificity analysis to evaluate the structural learning capacity of the latent space in aptamer generation. BE and PRO differed from CS by only three functional groups, and TES differed from BE and PRO by only one functional group (Fig. S1), thus providing a basis for similarity comparisons. As shown in Figs 3c–e, the DL-SELEX-generated aptamers displayed high specificity, exhibiting at least a 10-fold affinity difference against other steroid targets, particularly those included as counter-selection targets (CS/TES, CHO, and DHEA). Notably, DL-SELEX-generated TES aptamers showed closer affinity to BE (K_D_ = 6.22 μM) than PRO (K_D_ = 9.38 μM), likely due to the greater structural similarity between TES and BE. This indicates the feasibility of using structurally guided methods to generate highly specific aptamers from pre-defined libraries. The observed pattern was not evident for CS-R7MP-T, potentially owing to selection bias (Fig. S3). The full ITC results are shown in Figs S21–S65 and Table S6.

**Figure 4:**
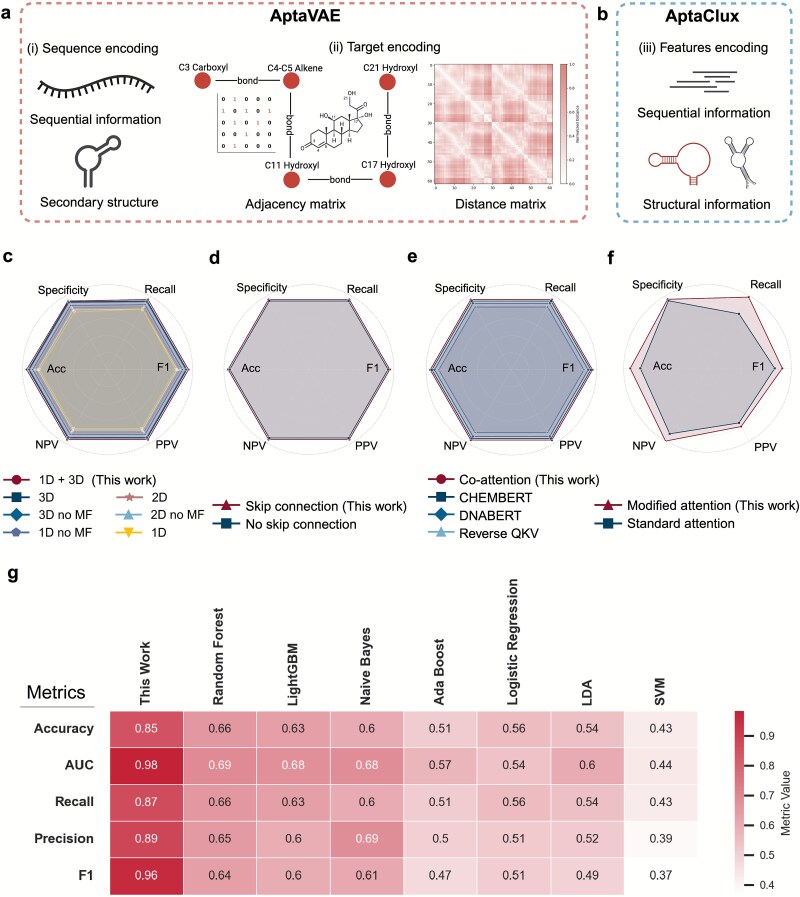
Deep learning models key pre-processing models, ablation study, and methods performance comparison. (a) Encoding methods for AptaVAE. AptaVAE encoding integrates sequence and target embeddings, with a sequence encoding capturing primary sequence and secondary structural information and target encoding incorporating distance and adjacency matrices. (b) AptaClux encoding combines secondary structural information of the sequence with primary sequence features. (c–e) Model ablation study for AptaVAE. (c) Comparison of performance across encoding dimensions, 1D (sequential encoding only), 2D (sequential + target distance matrix), and 3D (sequential + target distance matrix + target adjacency matrix). “MF” stands for Morgan fingerprints. (d) Evaluation of the skip connection module’s impact on enhancing sequence scoring features. (e) Co-attention encoding combining DNABERT and CHEMBERT embeddings. “Reverse QKV” indicates a setup with sequence as query and target as key and value; the standard configuration uses the target as query and sequence as key and value. (f) Modified attention mechanism by adding a query passthrough term. (g) Performance comparison of AptaVAE against conventional machine learning methods using five evaluation metrics. The abbreviations are for light gradient-boosting machine (LightGBM); linear discriminant analysis (LDA); support vector machine (SVM).

We evaluated various model modules and design approaches to create high-quality initial libraries efficiently. The data were pre-processed using a primer refinement program (Note S4). To capture structural information effectively, we implemented enhanced encoding mechanisms for sequences and targets in AptaVAE and AptaClux, combining structural encoding with linear sequential and secondary structure encoding (Fig. 4a and b). The ablation study (Fig. 4c) showed that integrating 1D and 3D encodings with skip connections and co-attention achieved the best performance (F1 = 0.92), enhancing the recall, precision, and specificity. The 1D encoding of integrated sequence information, Morgan fingerprints, class, and score significantly improved performance. Inspired by spatiotemporal models, 1D and 3D encodings were separated to address the sequential and spatial properties of aptamer data. The 3D encoding strategically positioned aptamer structures between the target distance and adjacency matrices (“sandwich” configuration, Fig. S2). Dilution issues from sparse 3D tensor integration were resolved by adding skip connections (Fig. S3), thus improving class prediction accuracy and overall F1 performance (Fig. 4d).

Given the limited dataset of 195 steroid-aptamer pairs, we considered that encoding alone was insufficient to capture consensus sequence information. To enhance the feature interaction, we incorporated an attention mechanism with pretrained DNABERT [35] and CHEMBERT [36] for DNA and chemical structure encoding, respectively. By treating the target as the query, the sequence as the key, and sequence embeddings as the value, the co-attention mechanism identifies aptamers most likely to bind to a given target. This integration improved the performance by 20% compared with using 1D and 3D encodings alone (Fig. 4e). Independent ablation studies further confirmed the usefulness of DNABERT and CHEMBERT, improving the model specificity by 24% (Fig. 4e).

As no salient established baseline exists for the deep learning-guided initial library task, we first performed a statistical analysis on our AptaVAE-guided initial library for CS, TES, and BE (Figs S73–S75). Overall, the statistical validation shows that AptaVAE-generated sequences are significantly different from random sequences across all measured parameters, with most showing medium to large effect sizes. This demonstrates that AptaVAE is not just generating random sequences but is learning to produce sequences with specific biological characteristics that are more likely to be functional aptamers. We also compared the performance of AptaVAE to traditional ML models on the same collected dataset. The AptaVAE outperformed all traditional models by at least 20% across metrics, including accuracy, AUC, recall, precision, and F1 score, demonstrating its superior capability in aptamer selection compared to other conventional ML models (Fig. 4f).

We further assessed the performance of structurally guided AptaVAE using MDs and docking. A significant challenge in computational biology is validating the model-generated outputs [46]. This involves assessing whether the AptaVAE-generated pre-defined library contains bases that are involved in the aptamer-ligand interaction. We evenly selected four aptamers from CS and TES from DL-SELEX for MD and docking simulations to investigate the potential binding sites. These aptamers were experimentally validated as true binders against their respective targets by ITC.

**Figure 5:**
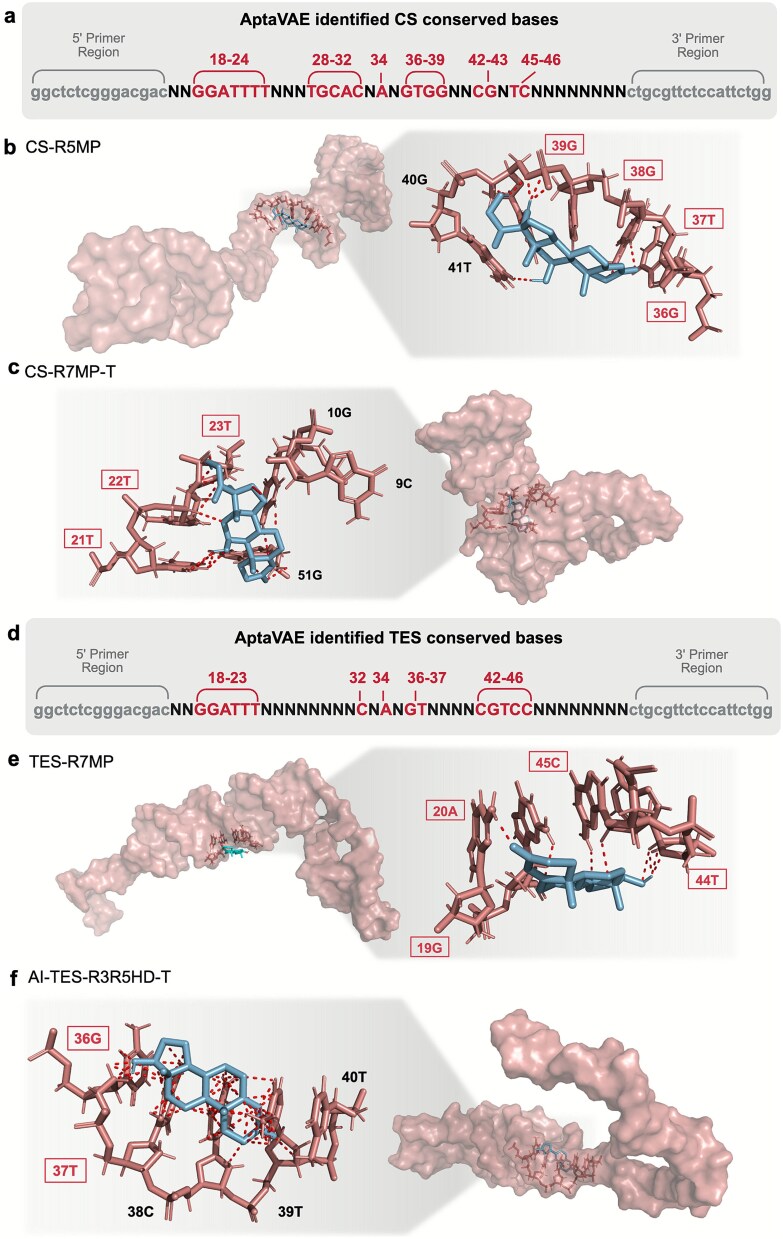
Molecular dynamics and docking analysis of aptamers targeting hydrocortisone (CS) and testosterone (TES). (a) AptaVAE identified pre-defined libraries for CS. The red-colored bases represent the confined bases. (b and c) Case study for the CS aptamers, showcasing potential binding sites identified through a 300 ns molecular dynamics simulation. The red dotted lines represent the hydrogen bonding of the aptamer to the ligand. (d) AptaVAE identified pre-defined libraries for TES. The blue-colored bases represent the confined bases. (e and f) Case study for the TES aptamer, highlighting potential binding sites.

For the CS aptamer CS-R5MP, the MD and docking (Fig. 5b) results suggest that the primary hydrogen bonding interactions occur at bases 36G–39G, with hydrophobic interactions at 37T. These bases were correctly predicted by AptaVAE (Fig. 5a) in the CS pre-defined initial library, supporting the accuracy of the model in identifying potential binding sites. To test whether these alignments were coincidental, we analyzed CS-R7MP-T (Fig. 5c), a truncated aptamer generated using our global truncation method. The predicted hydrogen bonds occurred at 21T and 22T, with hydrophobic interactions at 9C and 10G. Notably, bases 21T and 22T were also within the predicted binding region, further validating our model.

For the TES aptamer TES-R7MP, docking results (Fig. 5e) revealed binding interaction at 20A and 45C, along with hydrophobic interactions at 19G and 44T. These bases fell within the stem-loop region predicted by AptaVAE (Fig. 5d). Similarly, for the truncated TES aptamer AI-TES-R3R5HD-T (Fig. 5f), generated from NGS-VAE clustering, MD results suggested hydrogen bonds at 36G and 39T, with a hydrophobic interaction spanning bases 37T to 40T due to folding of linear DNA regions. Bases 36G and 37T match the pre-defined binding regions of AptaVAE. The AptaVAE predictions were consistent with the docking results across all test cases. Interestingly, although the binding positions aligned with the predicted regions, they did not always correspond to identical bases (Discussion in Note S5 [47]).

We next probed the MD/docking-predicted contacts by targeted point mutagenesis followed by ITC (Figs S71–S72, Note S9). In the CS aptamer (CS-R5MP), mutations were confined to the 36–39 motif that MD implicated in direct ligand contacts: Mut-1 perturbs a single contact with minimal fold disruption, whereas Mut-2 breaks the local motif. Consistent with the predictions, affinity deteriorated stepwise relative to wild type (K_D_ ≈ 2.41 μM): Mut-1 showed weak binding (K_D_ ≈ 3.95 mM) and Mut-2 was essentially nonbinding (K_D_ ≈ 192 mM). In the TES aptamer (AI-TES-R3R5HD-T), Mut-1 disrupted the first stem-loop (positions 36–37) and Mut-2 the second stem-loop (19–20); both mutants produced oscillating isotherms by ITC (no measurable K_D_), whereas wild type bound with K_D_ ≈ 0.323 μM. Together, these results provide experimental corroboration of the MD/docking assignments: the predicted contact motif in CS and the two TES stem-loops are functionally required for high-affinity binding.

**Figure 6:**
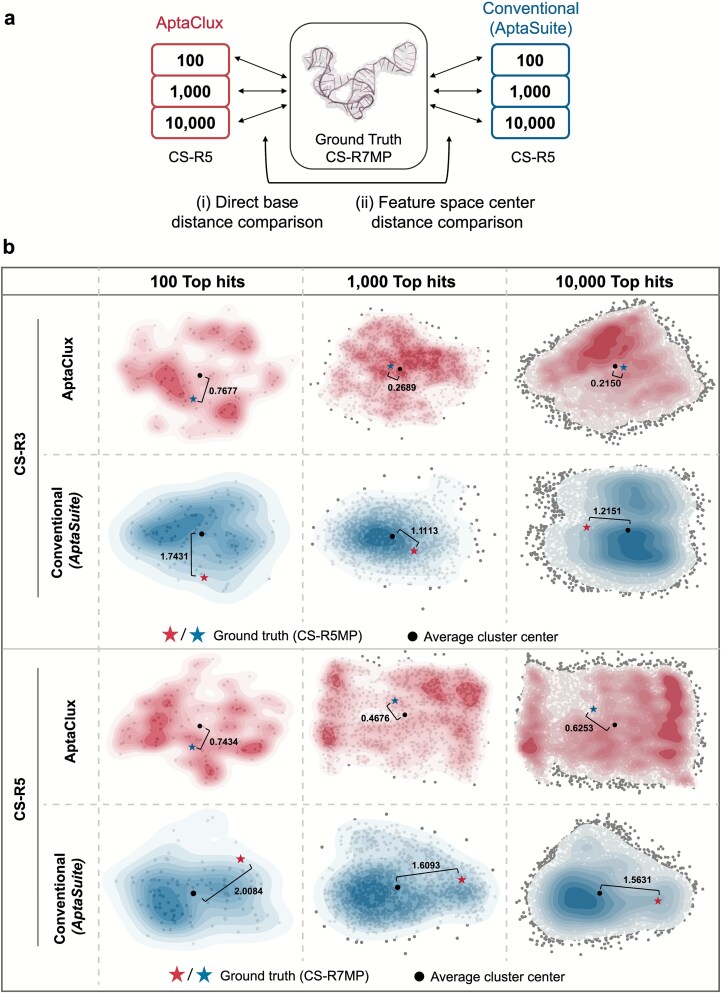
Validation of top AptaClux hits via direct base distance comparison and feature space analysis. (a) Workflow illustrating two approaches for top-hit validation: a. (i) direct base distance comparison, with results detailed in Table S5, and a. (ii) feature space center distance comparison using principal component analysis (PCA). (b) Feature space comparison visualized using PCA. Top half: Comparison of conventional and AptaClux generated sequences from round 3 with ground truth sequences in round 5 (CS-R5MP). Results are shown for the top 100, 1000, and 10000 hits (left to right columns). Bottom half: Comparison of conventional and AptaClux-generated sequences from round 5 with ground truth sequences in round 7 (CS-R7MP). Similar performance metrics are presented for the top 100, 1000, and 10 000 hits (left to right columns).

To address the challenge of evaluating a sequence generative model, we established two direct comparisons (Fig. 6a) between our AptaClux-generated top hits and those generated using the conventional AptaSuite [37] method. AptaSuite is a widely used open-source bioinformatics tool for HT-NGS post-analysis, but its clustering methods rely solely on sequence alignment and lack consideration of structural information within NGS datasets. See Note S6 for the rationale behind the selection of the ground-truth aptamer sequences.

We first calculated the average edit distance, which is a direct measure of the base-level differences between the model-generated sequences and the ground truth. The results (Table S5) showed that AptaClux-generated aptamers had, on average, two fewer base differences than AptaSuite across the top three hit classes (100, 1000, and 10000). This indicates that AptaClux-generated aptamers are more sequentially similar to the ground-truth aptamer. To further validate the top-hit comparisons, we projected the selected aptamers onto the two components using principal component analysis (PCA) (Fig. 6b). Model predictability was evaluated by comparing the average cluster centers to ground truth projections, where shorter distances indicated superior performance. HT-NGS summarization tools aim to identify high-affinity binders after fewer SELEX rounds. AptaClux consistently demonstrated shorter distances to ground truth projections, at least two-fold better than conventional methods in round 3, and nearly three-fold better in round 5 compared to the round 7 ground truth aptamer (CS-R7MP). This underscores the enhanced ability of AptaClux to generate high-quality aptamers.

Additionally, to benchmark AptaClux against modern generative approaches, we performed a head-to-head ranking comparison with AptaDiff and RaptGen under a single, model-agnostic protocol. (Methods Note S8; composition in Table S9). AptaClux achieved perfect separation on the panel (AUROC = 1.00; AUPRC = 1.00; Top-1 = 1.00), while AptaDiff [19] and RaptGen [15] were lower (Figs S67–S68), consistent with the per-method ranking strips and ROC/PR curves (Figs S69–S70). These results align with our observation that structure-enhanced clustering captures consensus features beyond round-wise abundance, and they rationalize why AptaClux-generated candidates anticipated the experimentally validated binders in CS and TES.

## Discussion

This study provides compelling evidence supporting the feasibility of a functional group-based framework for *de novo* aptamer design. Using the steroid family as an example, our findings demonstrate that structural inference from aptamer folding significantly enhances ligand binding, reduces the number of required SELEX rounds, and improves steroid aptamer performance. By dissecting aptamer structural features and leveraging deep learning to summarize common structural backbones, our approach efficiently improves selection quality, reduces time, and aligns with the broader aspiration of designing high-quality aptamers *de novo*. Currently, extensive data collection to account for experimental variability limits such generalization, where our framework pragmatically focuses on known structural features, applying these insights to structurally related targets. Our approach, leveraging the conserved four-ring carbon backbone of steroids, highlights the structural commonalities that enhance aptamer design for diverse targets. Steroids such as CHO and DHEA, which lack aptamers, differ minimally from established steroid targets (e.g. CS, TES), primarily in their functional groups (hydroxyl, ketone, and methyl modifications). These subtle structural differences are critical for the aptamer specificity. The next step is to apply DL-SELEX to design aptamers for unstudied steroids, such as CHO and DHEA, by incorporating functional group feature encodings. Moreover, expanding the structural data across molecular families could extend our functional group-based framework to nonsteroid targets, advancing the general *de novo* aptamer design.

Experimental validation remains the gold standard for assessing the aptamer quality. Obtaining nuclear magnetic resonance (NMR) or crystallographic data for all aptamers is infeasible. Thus, MDs and docking simulations are valuable tools that bridge computational predictions and experimental data by simulating aptamer-ligand interactions [48]. Despite skepticism due to their limitations, simulations combined with experimental data have improved reliability and cost-effectiveness [47]. Here, MD and docking simulations identified key binding sites for AptaVAE-generated aptamers (Note S7 [48]), demonstrating the feasibility of a structure-enhanced deep-learning approach for guiding initial library design.

A key contribution of this study is the integration of deep learning into the initial aptamer library design. Unlike traditional randomized libraries, AptaVAE incorporates target-specific structural insights, accelerating SELEX workflows while reducing the dependence on manual design, which is limited by human expertise and complexity. Previous computational approaches to initial library design include clustering and high-throughput virtual screenings [49]. Although useful, these methods face skepticism regarding their accuracy. Nevertheless, it is critical to reduce the initial pool search space. Conventional libraries often miss the structural constraints that are essential for high-affinity binding. Conversely, AptaVAE-designed libraries integrate functional group relationships and backbone features during pre-processing, thereby increasing structural interaction probabilities. Experimentally, AptaVAE-generated libraries achieved up to a 450-fold affinity improvement and 80% reduction in SELEX rounds. Interestingly, some ITC results indicated that aptamers from later rounds had a lower affinity than those from earlier rounds due to selection bias (Note S3). This suggests that earlier examination of the enriched pool in the SELEX process may be more advantageous. Our approach addresses the vast sequence diversity inherent in randomized libraries by tailoring the initial library to the structural characteristics of the target. This refinement ensures that high-quality candidates are present from the outset, enabling more targeted selection and reducing the time and costs associated with SELEX.

In addition to designing the initial libraries, DL-SELEX incorporates AptaClux, a post-selection module for rapid and accurate key aptamer summarization. Conventional bioinformatics tools, such as AptaSuite rank aptamers, are based on frequency, enrichment, or cluster analysis. However, our experimental results indicate that these criteria often fail to identify high-affinity binders (Figs S13–S14, Table S4). We attribute AptaClux’s strong performance to AptaVAE-guided pre-clustering of sequences within the same chemical family (e.g. steroids), which narrows the search space to structurally and energetically coherent motifs. With input pools already enriched for family-consistent features, AptaClux rapidly and accurately summarizes key variants from post-selection rounds, capturing consensus structures beyond abundance (Figs S13–S14, Table S4). Designed as a post-selection summarizer downstream of AptaVAE-curated libraries, this pairing underlies its efficiency and accuracy in our steroid studies. For raw NGS data lacking family guidance, users may also employ discovery tools such as (AptaDiff [19] or RaptGen [15]). Overall, AptaClux effectively complements DL-guided library design and streamlines SELEX workflows while remaining compatible with external tools.

## Conclusion

This study marks significant progress toward *de novo* aptamer design, although some challenges remain. Expanding datasets with diverse aptamer-target pairs will enhance generalizability, while developing reliable databases that account for experimental variability can improve predictive accuracy. Integrating advanced simulations with experimental workflows may further reduce the validation costs through multiplexed testing. We encourage researchers to explore the DL-SELEX pipeline (github.com/zibin-zhao/DL-SELEX) for other molecular families and to utilize the available tools for guided initial library generation and NGS summarization (http://hsingapp.ust.hk). The insights gained from this approach have broad implications for aptamer discovery, improving the selection efficiency and precision across biosensing, diagnostics, and therapeutics.

**Key Points**

- Introduced Deep Learning-assisted SELEX (DL-SELEX), a novel framework that integrates deep learning into SELEX library design and Next-Generation Sequencing (NGS) data analysis to accelerate small-molecule aptamer selection.
- Developed AptaVAE, a variational autoencoder (VAE), to generate optimized, structure-guided initial SELEX libraries tailored for specific ligand families like steroids, significantly enhancing the starting pool quality.
- Implemented AptaClux, a second VAE, to effectively analyze NGS data by extracting consensus structural features, enabling the identification of high-performing aptamer candidates from enriched pools.
- Achieved up to a 450-fold improvement in binding affinity for steroid aptamers (hydrocortisone and testosterone) and reduced SELEX experimental timelines by ~80% through the DL-SELEX pipeline.

## Supplementary Material

DL-SELEX-SI-final_bbaf680

SI-Extended-Figure-S19_bbaf680

## Data Availability

HT-SELEX experimental data for CS and TES from DL-SELEX are available via Zenodo (https://zenodo.org/records/14272647) and (https://zenodo.org/records/14272757), respectively. The manual design library HT-SELEX experimental data for CS are available via Zenodo (https://zenodo.org/records/14272347). AptaClux is available at http://hsingapp.ust.hk. The source code for DL-SELEX, including AptaVAE and AptaClux, is available for download via Zenodo (https://zenodo.org/records/14281818). The novel synthetic DNA aptamer used in this study was deposited in the European Nucleotide Archive (ENA) at EMBL-EBI under accession number PRJEB89545 (https://www.ebi.ac.uk/ena/browser/view/PRJEB89545).
