# Supplementary material for: Structure-enhanced deep learning accelerates aptamer selection for small molecule families like steroids
Source: Brief Bioinform. 2025 Dec 18;26(6):bbaf680. doi: 10.1093/bib/bbaf680 (PMC12713628; doi:10.1093/bib/bbaf680)
Supplement: DL-SELEX-SI-final_bbaf680 [file dl-selex-si-final_bbaf680.docx]

**Supporting Information**

**Structure-enhanced Deep Learning Accelerates Aptamer Selection for Small Molecule Families like Steroids**

Zibin Zhao^1,†^ , Haosi Lin^1,†^, Hoi Ying Lau^1^, Hao Chen^1,2,*^ and I-Ming Hsing^1,*^

^1^ Department of Chemical and Biological Engineering, The Hong Kong University of Science and Technology, Clear Water Bay, Kowloon, Hong Kong SAR, China

^2^ Department of Computer Science and Engineering, The Hong Kong University of Science and Technology, Clear Water Bay, Kowloon, Hong Kong SAR, China

^*^ To whom correspondence should be addressed. Tel: +852 2358 7122; Fax: +852 3106 4857; Email: kehsing@ust.hk. Correspondence may also be addressed to Tel: +852 2358 8346; Email: jhc@cse.ust.hk.

^†^ Zibin Zhao and Haosi Lin contributed equally to this work.

Contents

[Figure S1 | Chemical Structures of Steroids 4](#_Toc212557753)

[Figure S2 | AptaVAE Preprocessing Modules 5](#_Toc212557754)

[Figure S3 | AptaVAE Model Architecture 7](#_Toc212557755)

[Figure S4 – S9 | AptaVAE Latent Dimension Comparison 8](#_Toc212557756)

[Figure S10 | AptaVAE BGA Analysis 14](#_Toc212557757)

[Figure S11 | AptaVAE MSA Methods Comparison 15](#_Toc212557758)

[Figure S12 | AptaClux Encodings and Model Architecture 17](#_Toc212557759)

[Figure S13-S14 | Evaluation of Frequency and Enrichment with FRET 18](#_Toc212557760)

[Figure S15 | AptaClux MSA Logo Map for Two Clustering Methods-generated Aptamers 20](#_Toc212557761)

[Figure S16 | Molecular Dynamics and Docking Full Pipeline 21](#_Toc212557762)

[Figure S17 | FRET Experiment Protocol 22](#_Toc212557763)

[Figure S18 | FRET Results for Manual Design Library 23](#_Toc212557764)

[Figure S19 | MSA of Collected Steroid Aptamers 24](#_Toc212557765)

[Figure S20 | MD comparison of truncated aptamer versus non-truncated aptamer 25](#_Toc212557766)

[Figure S21 – S65 | ITC Results for DL-SELEX-generated Aptamers 26](#_Toc212557767)

[Figure S66 | Collected Steroid Aptamer Classes Distribution 71](#_Toc212557768)

[Figure S67-S70 | Deep Learning Models Performance Comparison 72](#_Toc212557769)

[Figure S71-S72 | Experimental Verification of Molecular Dynamics and Dockings Predicted Contacts via ITC 75](#_Toc212557770)

[Figure S73-S75 | AptaVAE Initial Library Statistical Validation 77](#_Toc212557771)

[Table S1 | Collected 195 Aptamer Sequences 80](#_Toc212557772)

[Table S2 | AptaVAE Latent Dimension Comparison Evaluation 89](#_Toc212557773)

[Table S3 | AptaVAE Generated Steroid Pre-Defined Library 90](#_Toc212557774)

[Table S4 | Summary Table of Evaluation on Frequency and Enrichment with FRET 91](#_Toc212557775)

[Table S5 | AptaClux Direct Bases Edit Distance Comparison 92](#_Toc212557776)

[Table S6 | Summary Table of Affinity (K_D_) Results from ITC 93](#_Toc212557777)

[Table S7 | Steroids Chemical Formula and SMILES Representation 94](#_Toc212557778)

[Table S8 | Aptamer Sequences Used in This Work 95](#_Toc212557779)

[Table S9 | CS Panel Aptamers for DL Models Performance Comparison 96](#_Toc212557780)

[Note S1 | Simplified and Standardized Materials and Workflow for Selection Experiment in DL-SELEX 97](#_Toc212557781)

[Note S2 | Naming Convention for DL-SELEX generated Aptamer Sequences 102](#_Toc212557782)

[Note S3 | Selection Biases Discussion 103](#_Toc212557783)

[Note S4 | Model Assumption and Primer Refinement Discussion 104](#_Toc212557784)

[Note S5 | Binding Position Prediction Discussion 105](#_Toc212557785)

[Note S6 | Ground Truth Sequence Selection Discussion 106](#_Toc212557786)

[Note S7 | Molecular Dynamics and Simulation Reveal Key Binding Sites 107](#_Toc212557787)

[Note S8 | Design Logic and Evaluation Protocol for DL Model Comparison (AptaClux, AptaDiff, RaptGen) 108](#_Toc212557788)

[Note S9 | Experimental Verification of Predicted Contacts via ITC Notes 110](#_Toc212557789)

[Note S10 | Modified Attention Mechanism Formalization 112](#_Toc212557790)

[Reference 113](#_Toc212557791)

## Figure S1 | Chemical Structures of Steroids

**
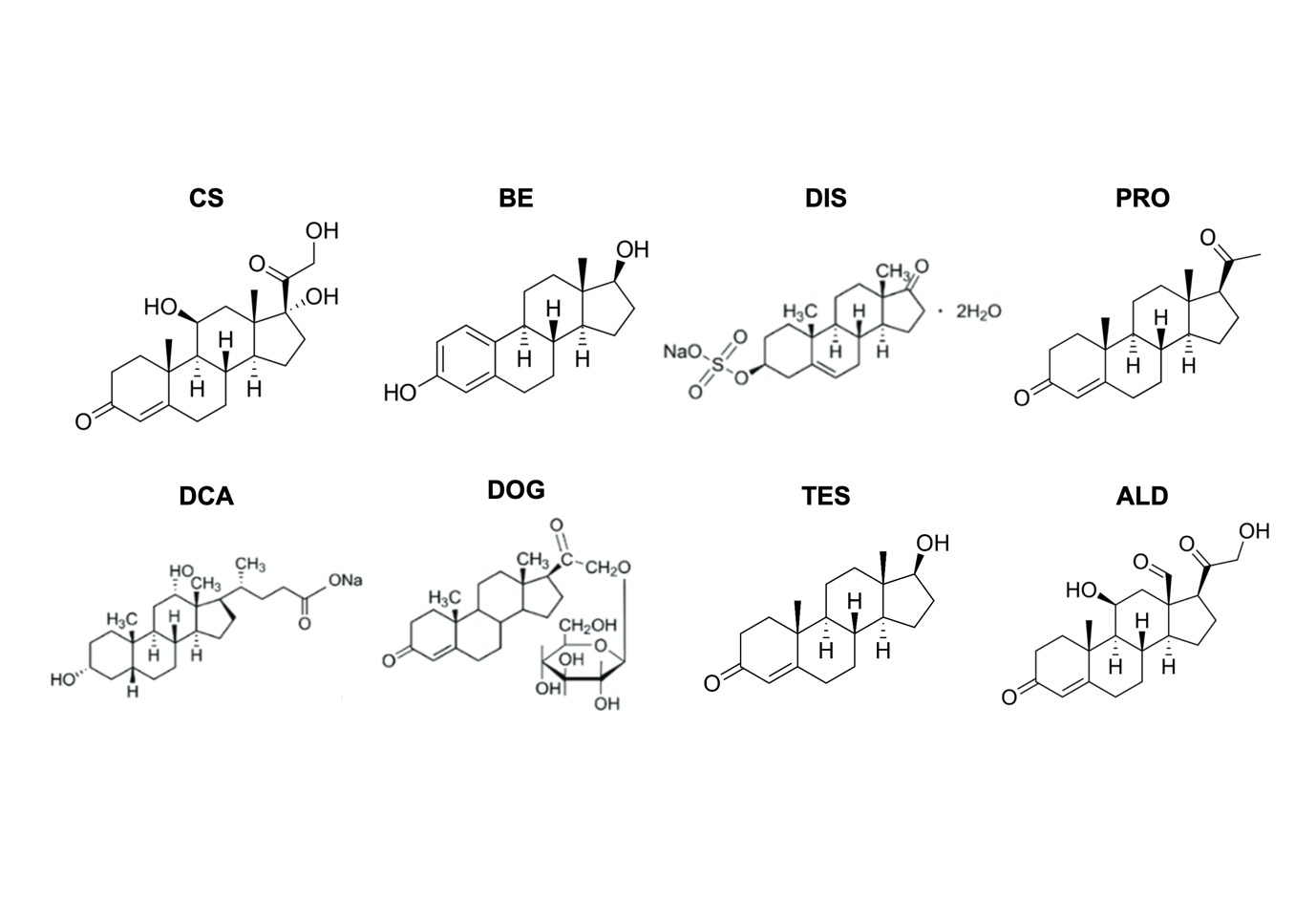
**

**Figure S1.** Chemical structures of all trained steroids. Hydrocortisone (**CS**), beta-estradiol **(BE),** dehydroisoandrosterone 3-sulfate (**DIS**), progesterone (**PRO**), deoxycholic acid sodium salt **(DCA),** deoxycorticosterone 21-glucoside (**DOG**), testosterone (**TES**), and aldosterone (**ALD**).

## Figure S2 | AptaVAE Preprocessing Modules

**
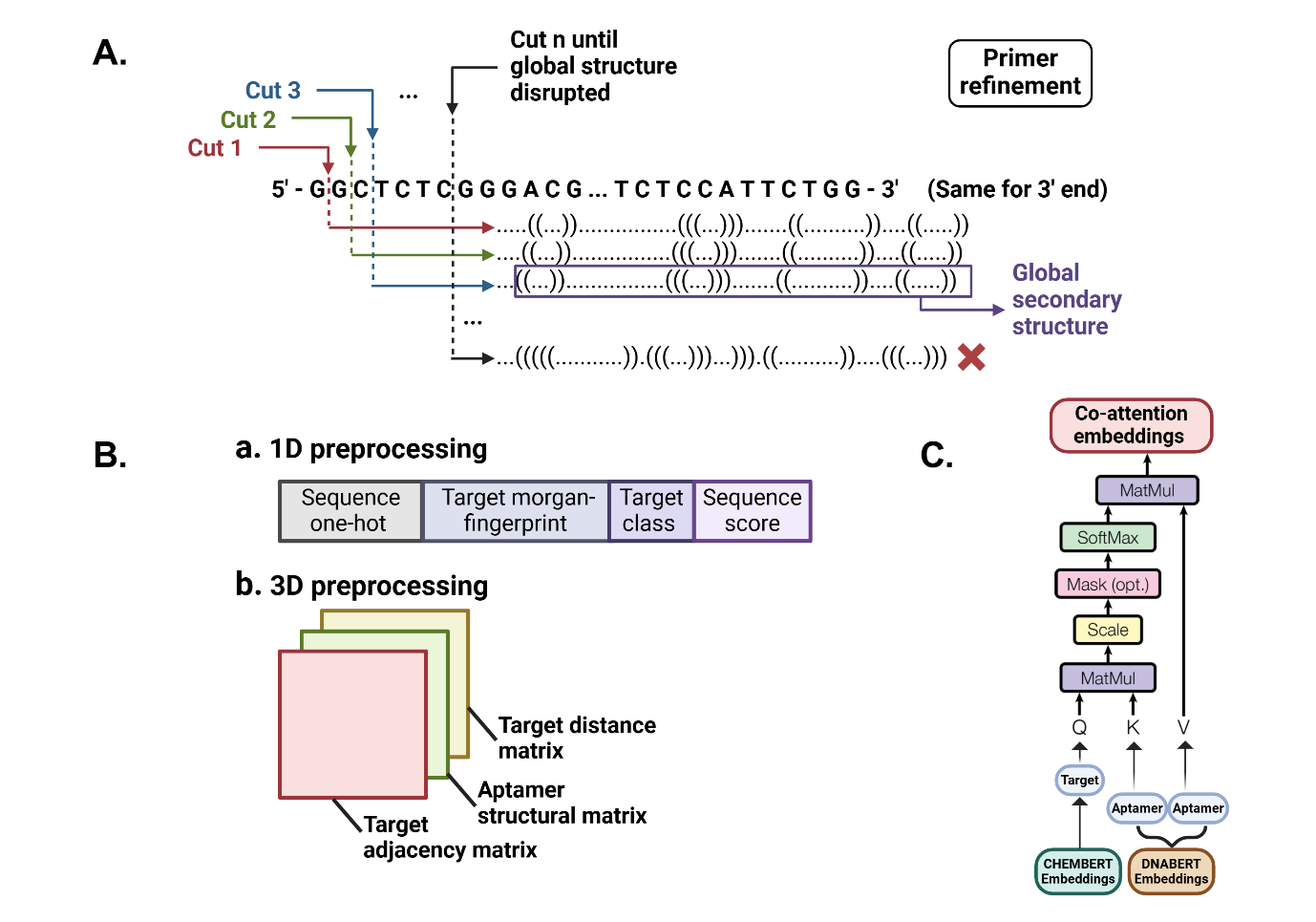
**

**Figure S2.** (A) Global secondary truncation program by a modified Python script, the script cuts the bases one-by-one until the original secondary structure by the NUPACK is differed from the last bases truncated one. The program will move to the 3-prime end for a similar truncation. (B) The AptaVAE encoding method includes both 1D and 3D preprocessing of raw sequences and their corresponding targets. The 1D preprocessing includes sequence one-hot, target Morgan fingerprint, target class, and sequence score. The 3D preprocessing includes the target’s distance and adjacency matrix and aptamer structural matrix in between. (C) Pretrained embedding of the sequence and target from DNABERT and CHEMBERT, respectively with a standard co-attention mechanism. Target is the query input and aptamers are the key and value inputs.

The truncation of all training sequences (N = 195) was performed using a modified Python script. The secondary structure of each sequence was first predicted using NUPACK with 25°C and 0.147 M potassium ion concentration. The script sequentially removed bases starting from the 5’ end, recalculated the secondary structure after each deletion, and compared it to the original global stem-loop structure. This process continued until the global secondary structure was disrupted, at which point truncation at that end was stopped. The same procedure was applied to the 3’ end.

Due to variations in sequence lengths, start and stop codons were included as additional classes to indicate the sequence boundaries, resulting in six one-hot encoding classes: four DNA bases and start/stop markers. Sequence scores were normalized to continuous values between 0 and 1. Target classes were derived from SMILES representations (Supplementary Table S7) and encoded using both one-hot encoding and Morgan fingerprints to enhance target recognition. The resulting encoded segments, sequence, target Morgan fingerprint, molecular class, and score were concatenated into a 1D tensor.

Since steroid aptamer sequences varied in length across sources, all sequences were padded with 0 and masked to a maximum length of 100 after primer refinement, ensuring compatibility for concatenation with other encoded segments. Targets, encoded with Morgan fingerprints (1024 bits) and their corresponding molecular classes, had uniform lengths and did not require padding. Similarly, padding and masking were applied during CHEMBERT and DNABERT encoding to produce uniform tensor outputs, enabling seamless concatenation for downstream processing.

## Figure S3 | AptaVAE Model Architecture

**
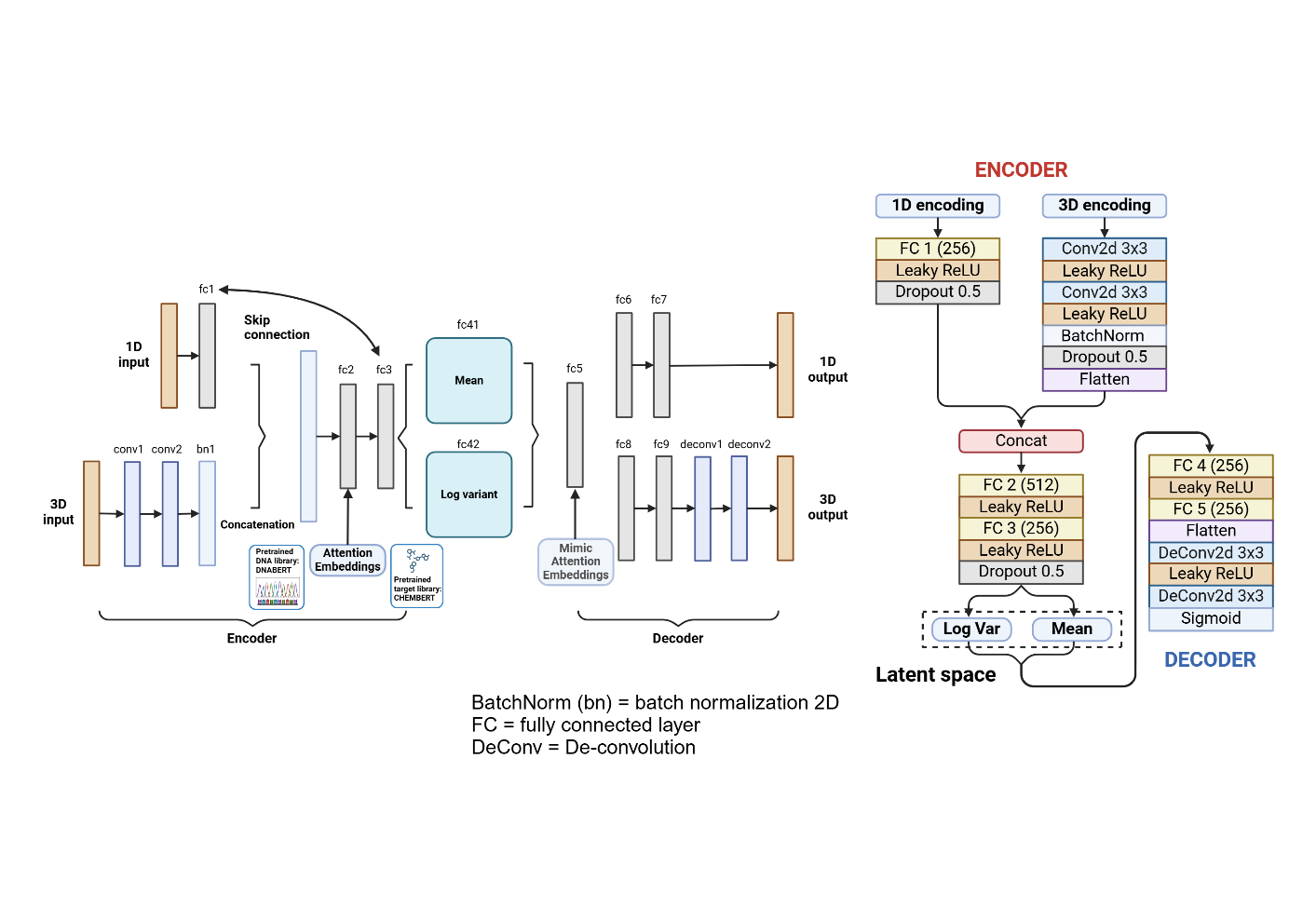
**

**Figure S3.** (Left) Overview of the AptaVAE model architecture including pretrained embeddings, 1D and 3D input, 1D and 3D decoding output. A mimic attention embedding was used for loss calculation accounting for the attention layer input loss. (Right) Detailed AptaVAE layers in between including all processing units inside the model. All models were trained on a 64 GB memory, RTX 4090 PC on an average of 72 hours of training time with a latent size of 256.

The AptaVAE comprises an encoder and decoder, where the encoder processes three input embeddings: 1D encoding, 3D encoding, and attention embeddings derived from DNABERT and CHEMBERT outputs. The attention embeddings were incorporated at a later fully connected layer. As the model's primary output is the sequence, we separated the 1D and 3D outputs while incorporating an artificial co-attention embedding component to support loss calculation.

## Figure S4 – S9 | AptaVAE Latent Dimension Comparison

**
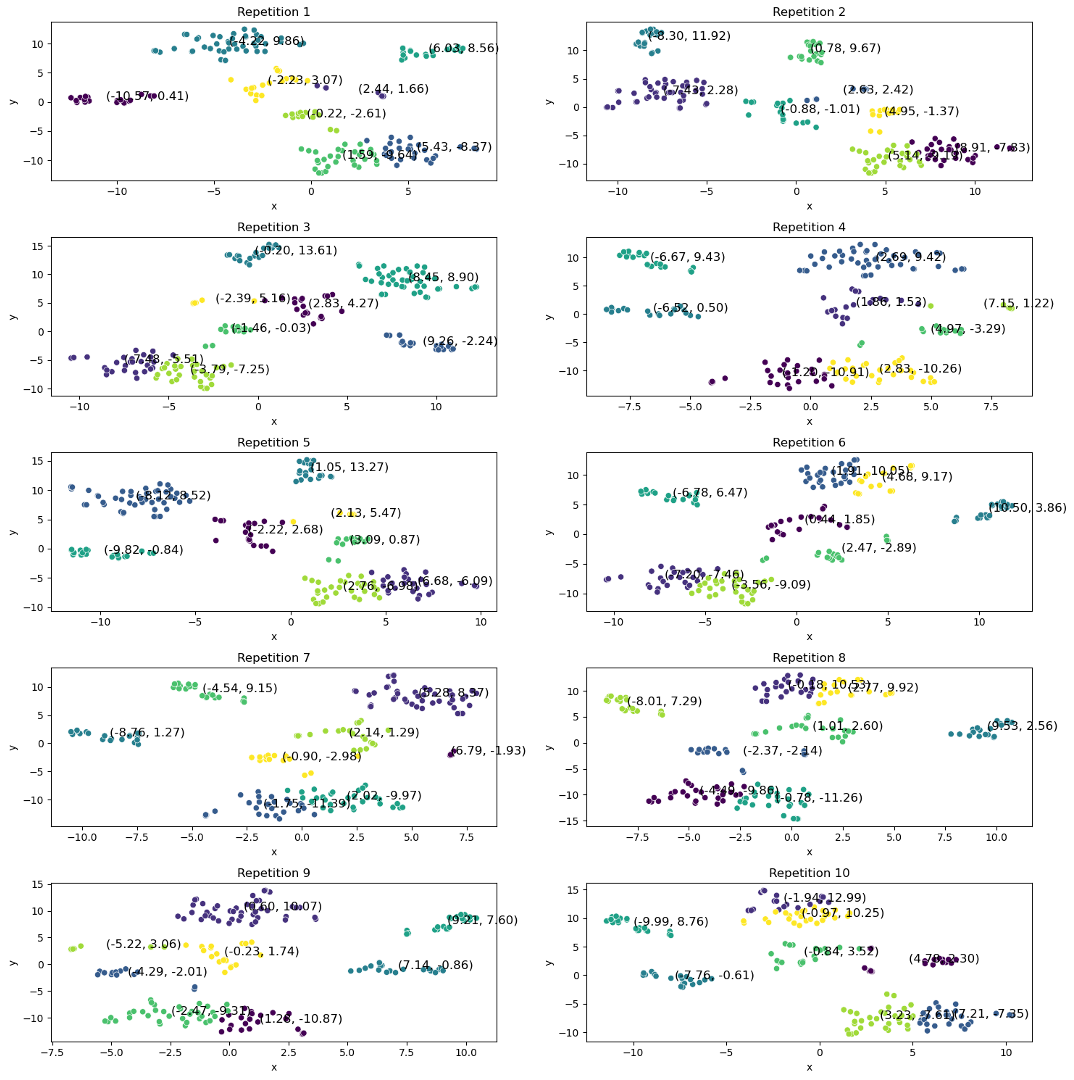
**

**Figure S4.** 10 repetitions of latent clustering of 8-dimensional space, which is the highest clustering score amongst all other dimensions (16, 32, and 64, 128 and 256)

**
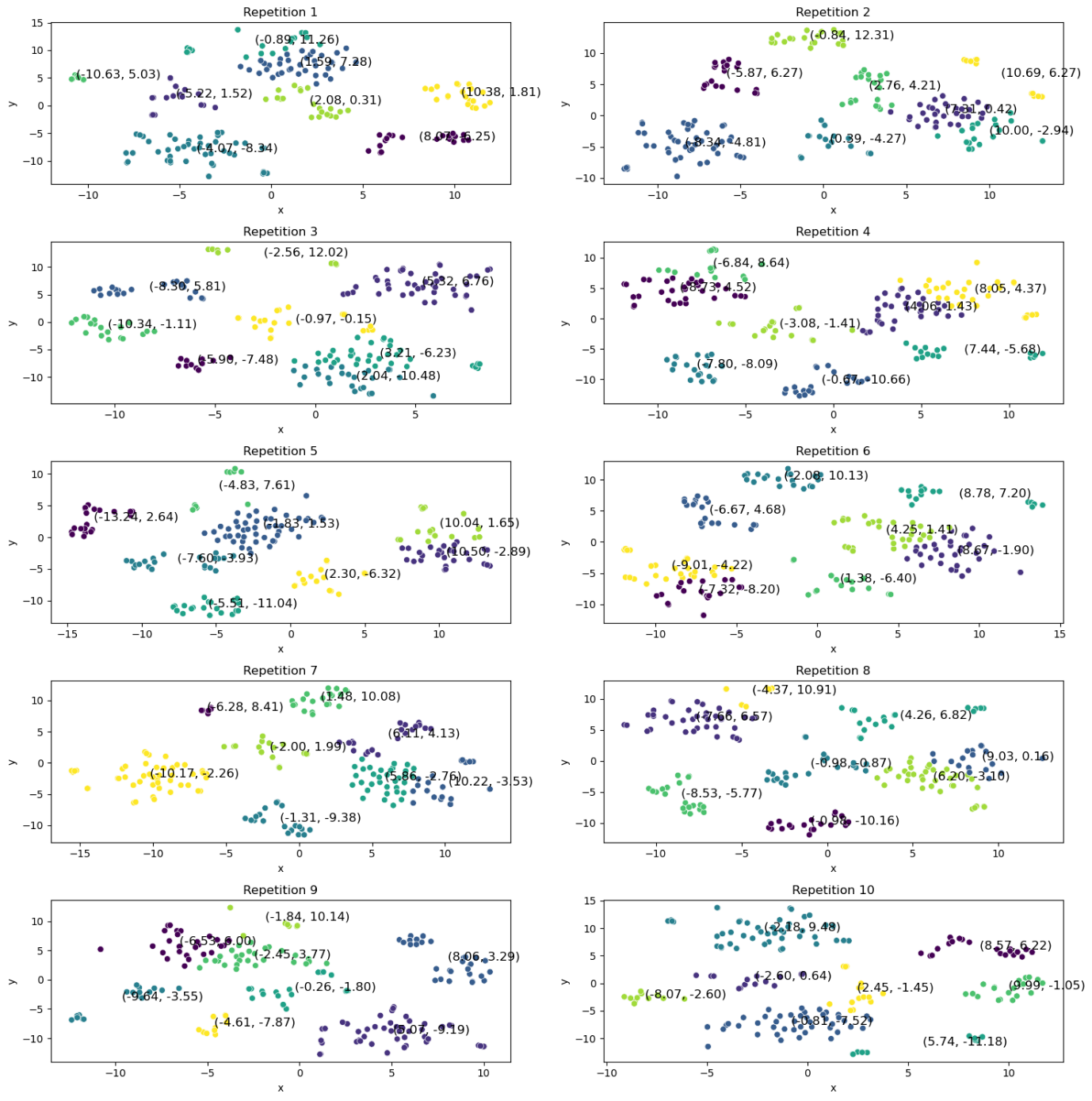
**

**Figure S5.** 10 repetitions of latent clustering of 16-dimensional space.

**
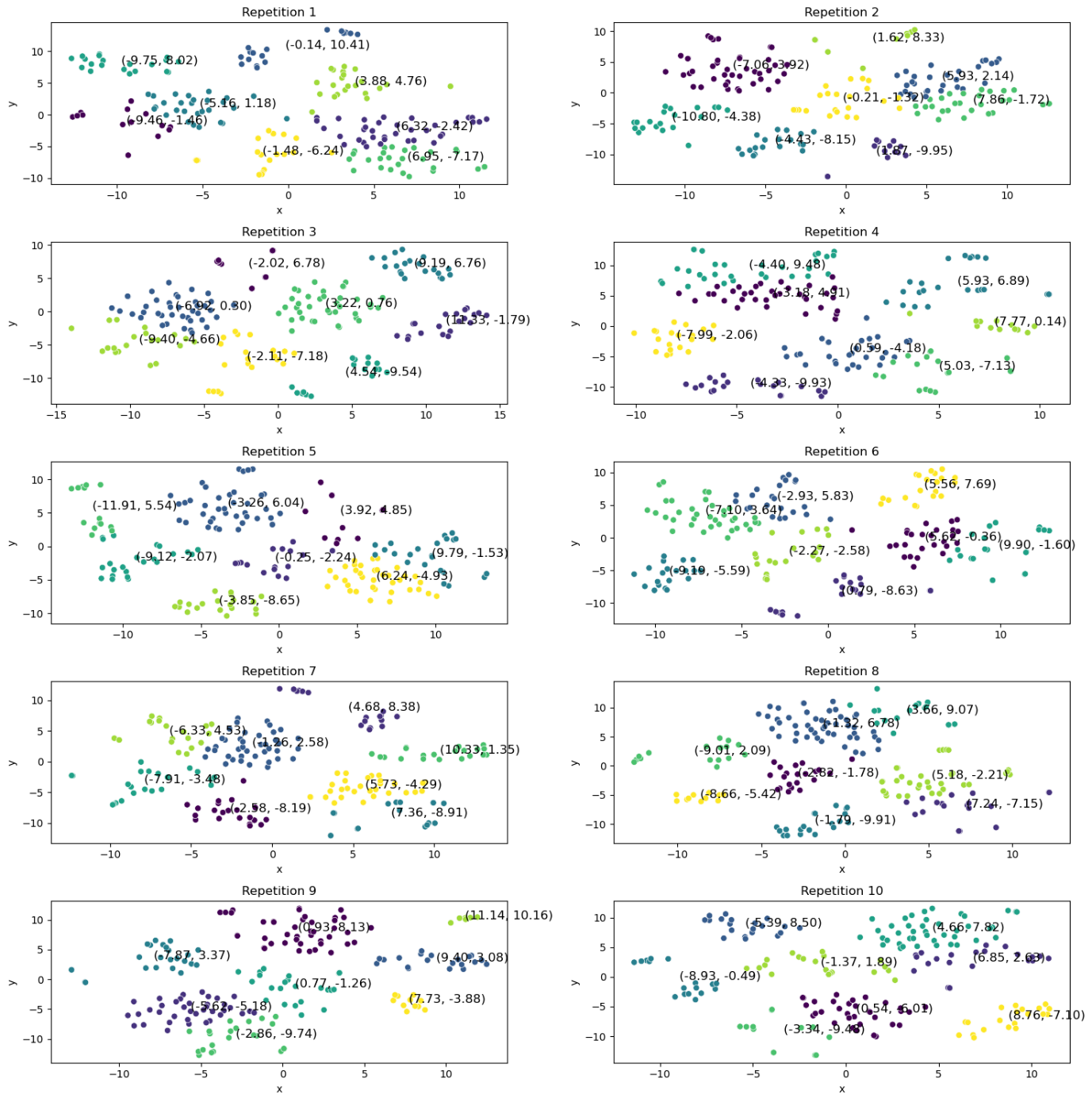
**

**Figure S6.** 10 repetitions of latent clustering of 32-dimensional space.

**
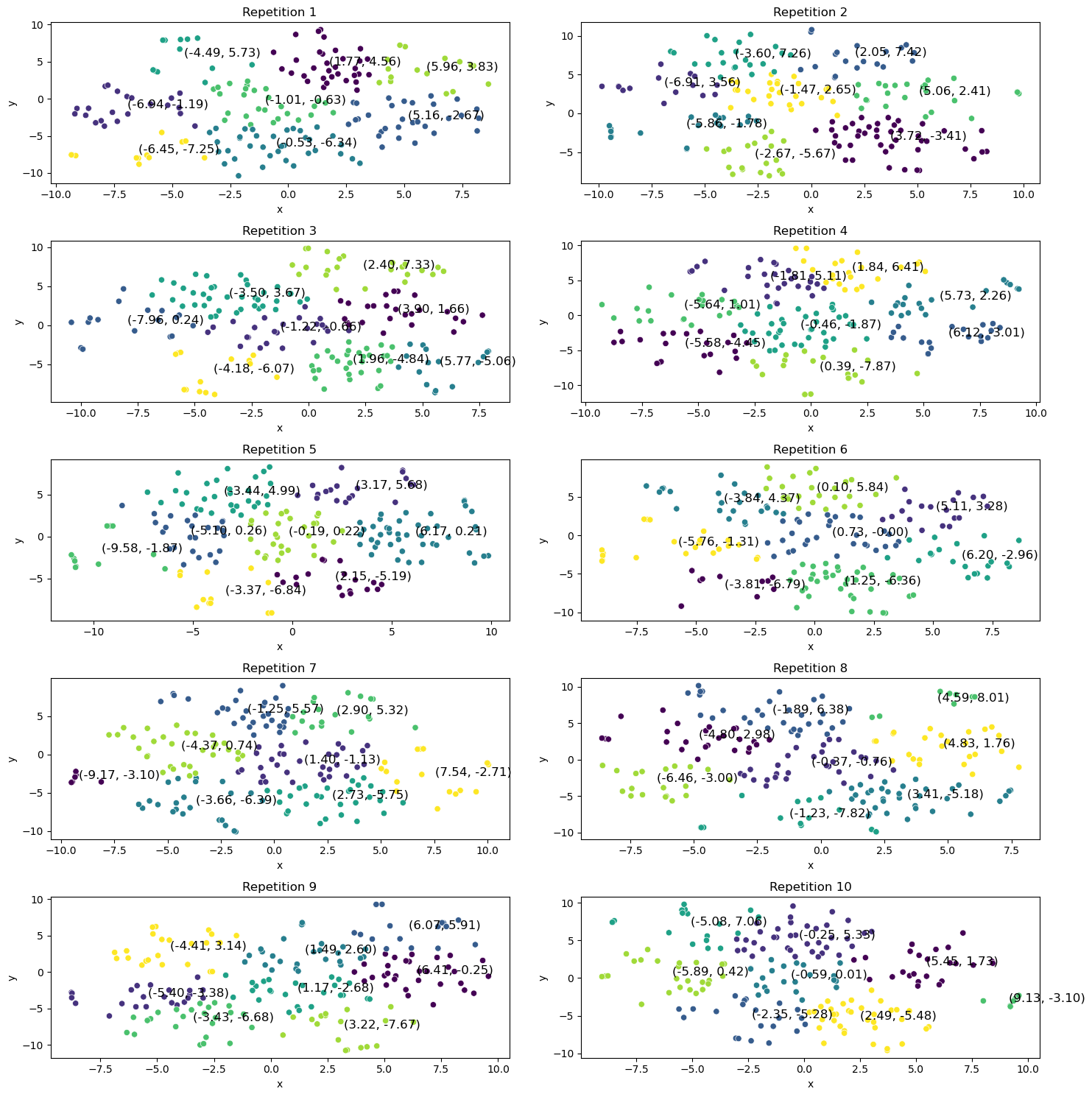
**

**Figure S7.** 10 repetitions of latent clustering of 64-dimensional space.

**
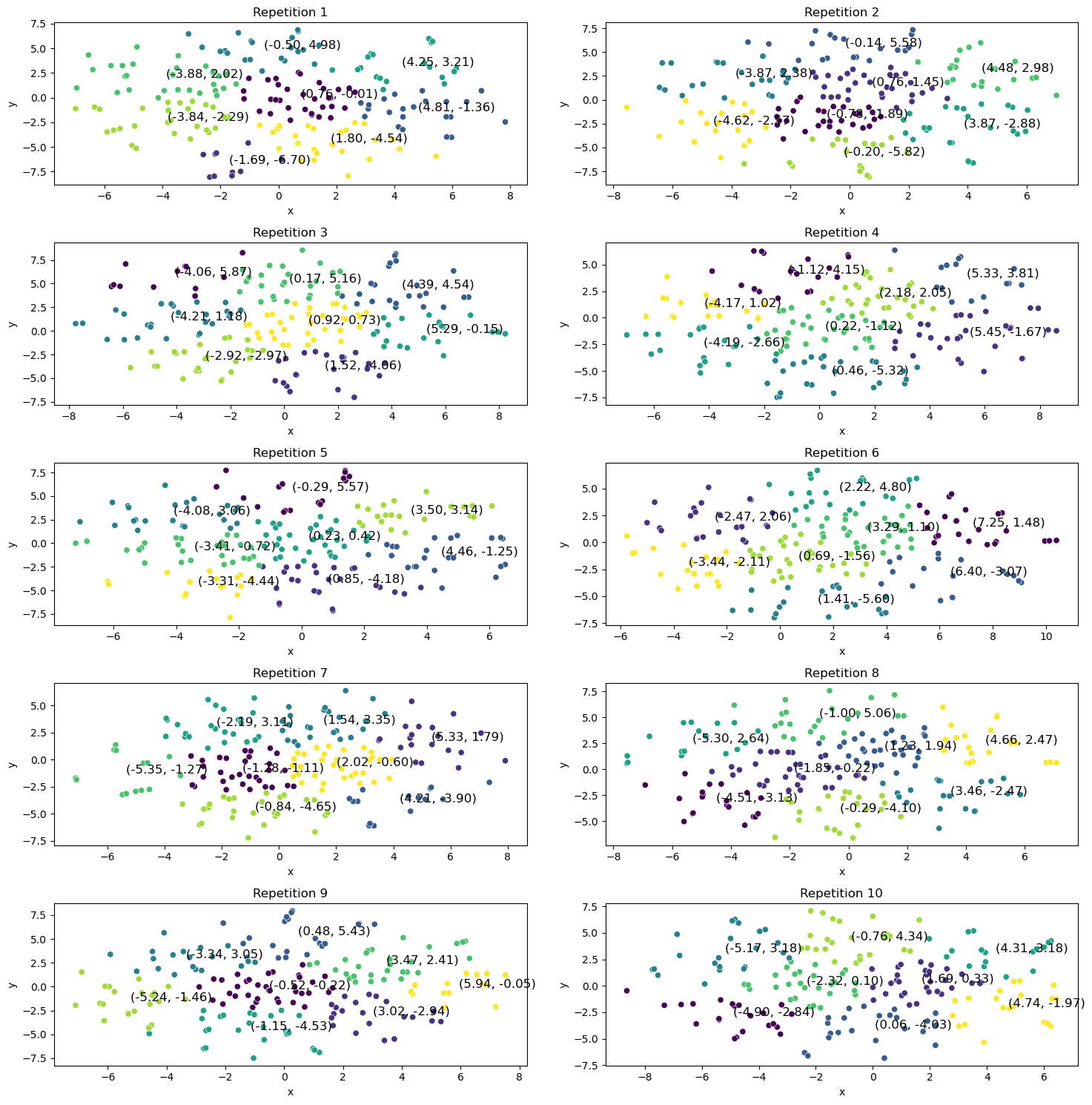
**

**Figure S8.** 10 repetitions of latent clustering of 128-dimensional space.

**
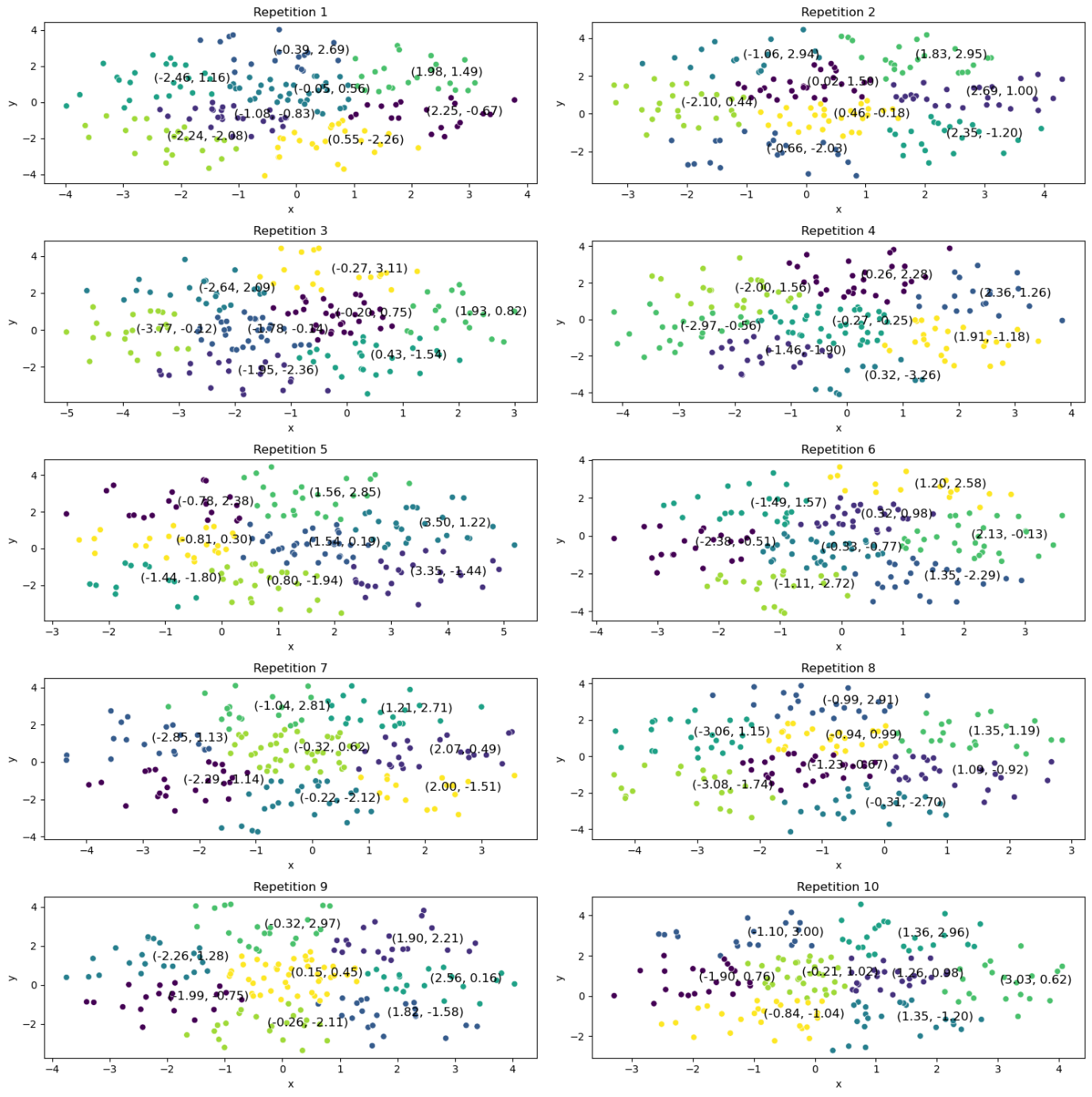
**

**Figure S9.** 10 repetitions of latent clustering of 256-dimensional space. Detailed analysis of the clustering results can be found in Table S2. Results indicated that increasing the latent dimension enhances the generation of higher-scoring sequences. In contrast, lower-dimensional latent spaces produced a smaller proportion of high-quality sequences. This observation aligns with the role of the VAE as a "regularizer" or information bottleneck. In summary, lower-dimensional latent spaces are more constrained, yielding fewer high-scoring sequences, whereas higher-dimensional spaces promote greater diversity. Based on these findings, we opted for high-dimensional latent spaces (256) for library generation (to maximize diversity) and low-dimensional latent spaces for tasks like Bayesian optimization in sequence generation (e.g., in AptaClux).

## Figure S10 | AptaVAE BGA Analysis

**
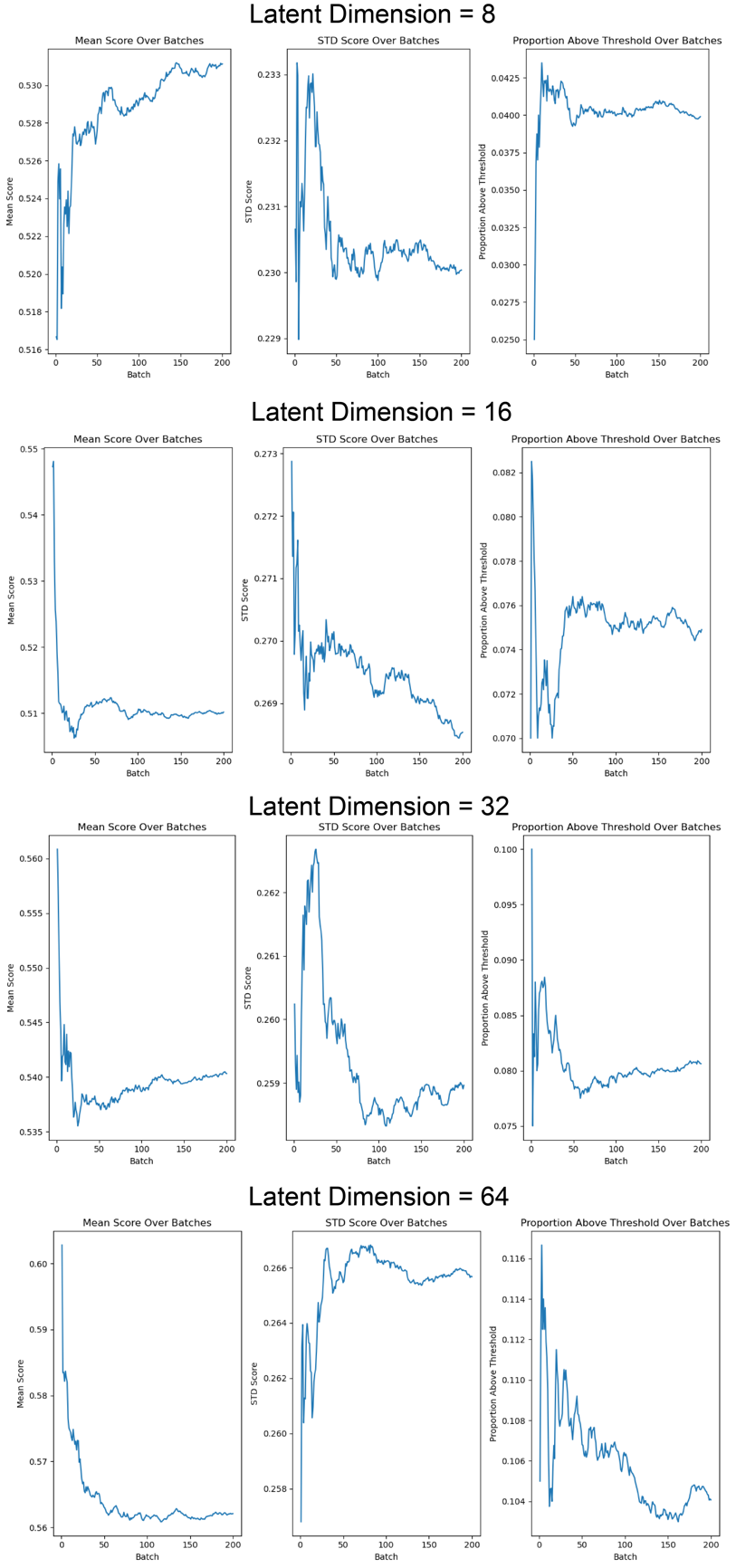
**

**Figure S10.** Batch generated analysis (BGA) of different latent space dimensions of 8, 16, 32, and 64. The actual sampling will occur at the plateau, where the model generative ability is more stable.

## Figure S11 | AptaVAE MSA Methods Comparison

**
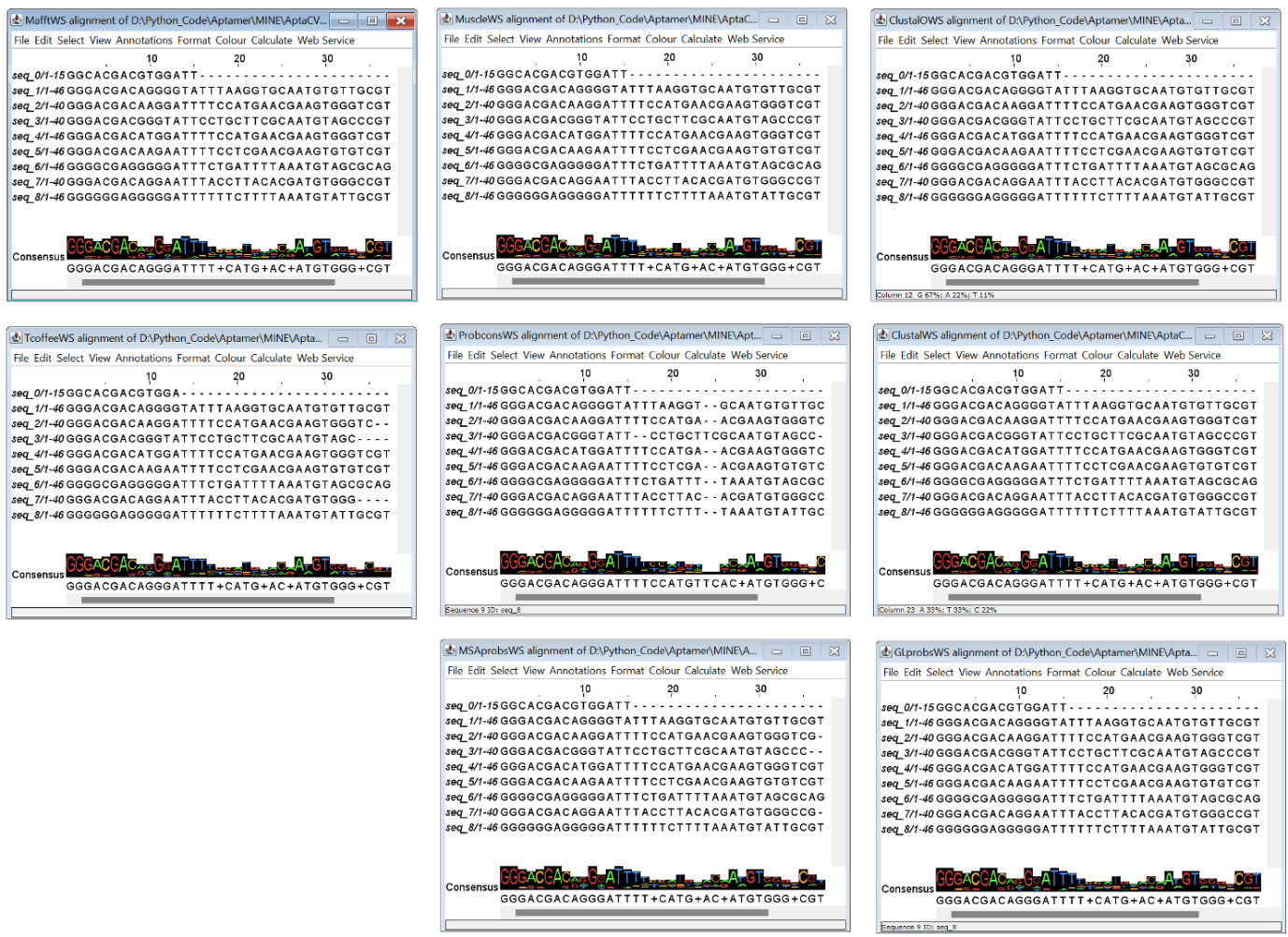
**

**Figure S11.** Different MSA methods (The method’s name is in the window top left) were performed using JalView (Version 2.11.4.0).

We observed that all methods returned similar results, with less than five base variations. This lack of significant difference highlights the challenge of ad hoc method selection, which can be suboptimal. Additionally, we noted that varying sequence lengths produced differing alignment outcomes.

In this study, we performed three repetitions using a 256-dimensional latent space, extracted the same region bases, and assigned "N" to ambiguous bases. For alignment, we selected MAFFT, which consistently produced the most common bases and was similar to results from other MSA methods. Mafft is widely used for short sequence alignments due to its enhanced accuracy, though at the expense of speed. This makes it a reliable choice for our alignment tasks. From the MSA results of the model-generated aptamers, it was observed that despite different libraries using varying primer sequences, refining primers that do not disrupt the global 2D structure consistently revealed a similar and conserved 5-prime end. This indicates that certain primer regions contribute to forming the stem of the binding loop, which is crucial for aptamer functionality. In contrast, the 3-prime end showed greater variability, with published 3-prime primers often failing to support 2D structural formation. These findings suggest that guided primer design may significantly increase the likelihood of generating high-affinity strands.

For each target, three independent repetitions were performed to validate that the generated library was consistently and reasonably derived from the latent space. MSA was conducted for each repetition, and conserved bases across all alignments were retained as fixed positions, while variable positions were designated as "N" to represent any nucleotide.

From the MSA results of the model-generated aptamers, it was observed that despite different libraries using varying primer sequences, refining primers that do not disrupt the global 2D structure consistently revealed a similar and conserved 5-prime end. This indicates that certain primer regions contribute to forming the stem of the binding loop, which is crucial for aptamer functionality. In contrast, the 3-prime end showed greater variability, with published 3-prime primers often failing to support 2D structural formation. These findings suggest that guided primer design may significantly increase the likelihood of generating high-affinity strands.

## Figure S12 | AptaClux Encodings and Model Architecture

**
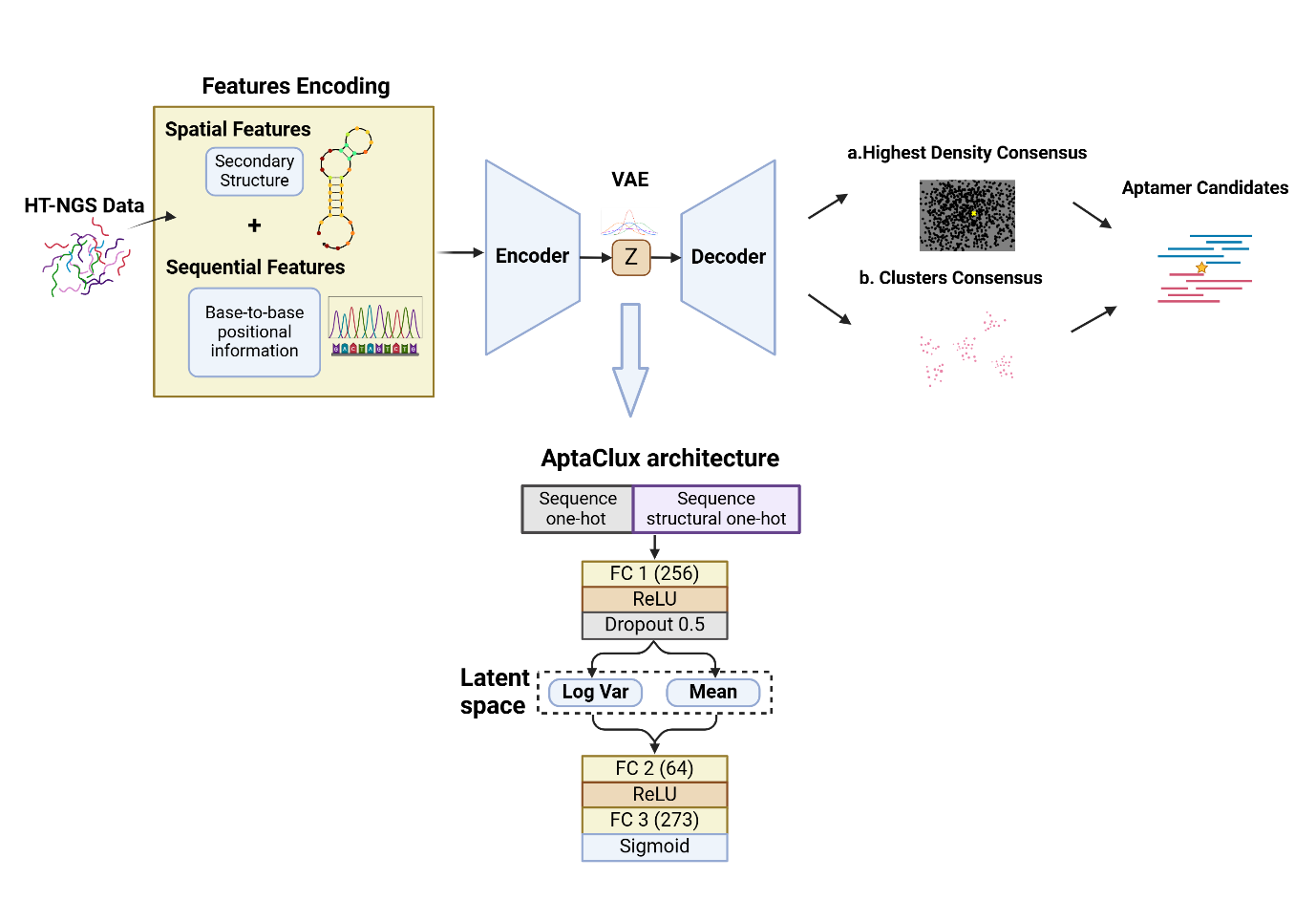
**

**Figure S12.** Encoding methods, including sequence information and secondary structural information of all High-throughput next-generation sequencing data (HT-NGS). The detailed modified AptaClux architecture is drawn below. ‘FC’ stands for fully connected layer, with a sigmoid at the end of the model output. The generated aptamer candidates from two clustering methods, highest density clustering, and 10 clusters consensus, were further used for ITC analysis. All models were trained on a 64 GB memory, RTX 4090 PC on an average of 24 hours of training time with a latent size of 256, the latent space clustering for ~200,000,000 NGS sequences takes about 72 hours on a 13^th^ Gen Intel® Core™ i9-13900K, 3.99GHz computer.

The latent space was trained on an average of 30 million sequences, enabling the generation of new aptamer candidates sampled from this space. The model utilized both sequence and secondary structure as dominant features, decoding the underlying shared attributes to produce aptamers with improved binding potential. The model loss function incorporated both sequence and structure losses, all calculated using binary cross-entropy.

## Figure S13-S14 | Evaluation of Frequency and Enrichment with FRET

**Figure S13. Top 3 Frequency** of CS aptamers from round 8 in the manually designed library SELEX NGS data.

These FRET experiments were designed to test the conventional method of using frequency and enrichment as the scoring function to evaluate an aptamer’s performance. However, in our experiment, no clear trend or relationship between frequency/enrichment and binding affinity was observed, suggesting these metrics should not be utilized as the sole scoring function.

**Figure S14. Top 3 Enrichment** of CS aptamers calculated from round 8 over round 7 in the manually designed library SELEX NGS data.

## Figure S15 | AptaClux MSA Logo Map for Two Clustering Methods-generated Aptamers

**
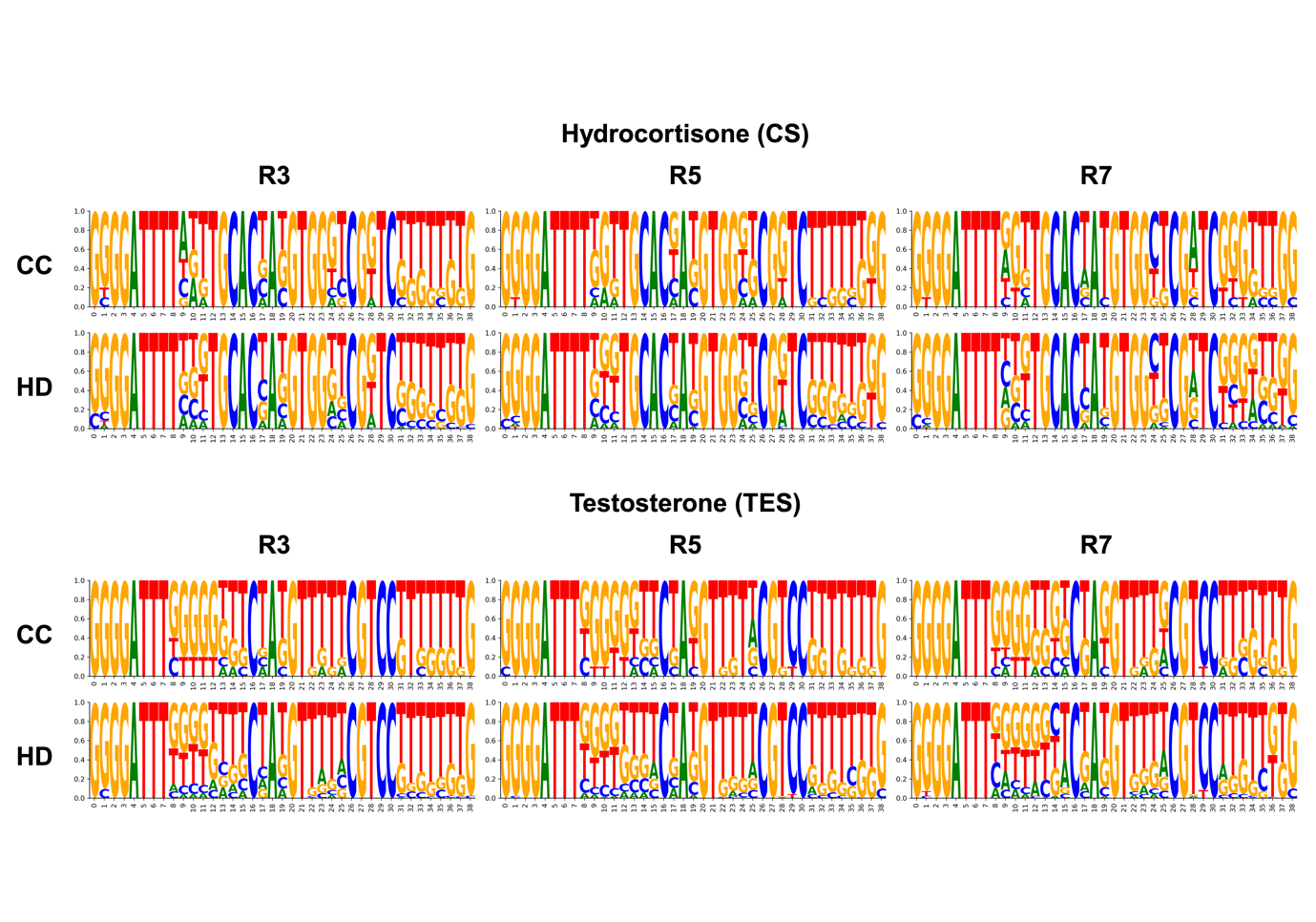
**

**Figure S15.** Sequence logo map created by custom Python script on CS and TES for rounds 3, 5, and 7. ‘CC’ stands for cluster consensus created from the k-mean clustering method, and ‘HD’ stands for high density created from Gaussian distribution clustering.

## Figure S16 | Molecular Dynamics and Docking Full Pipeline


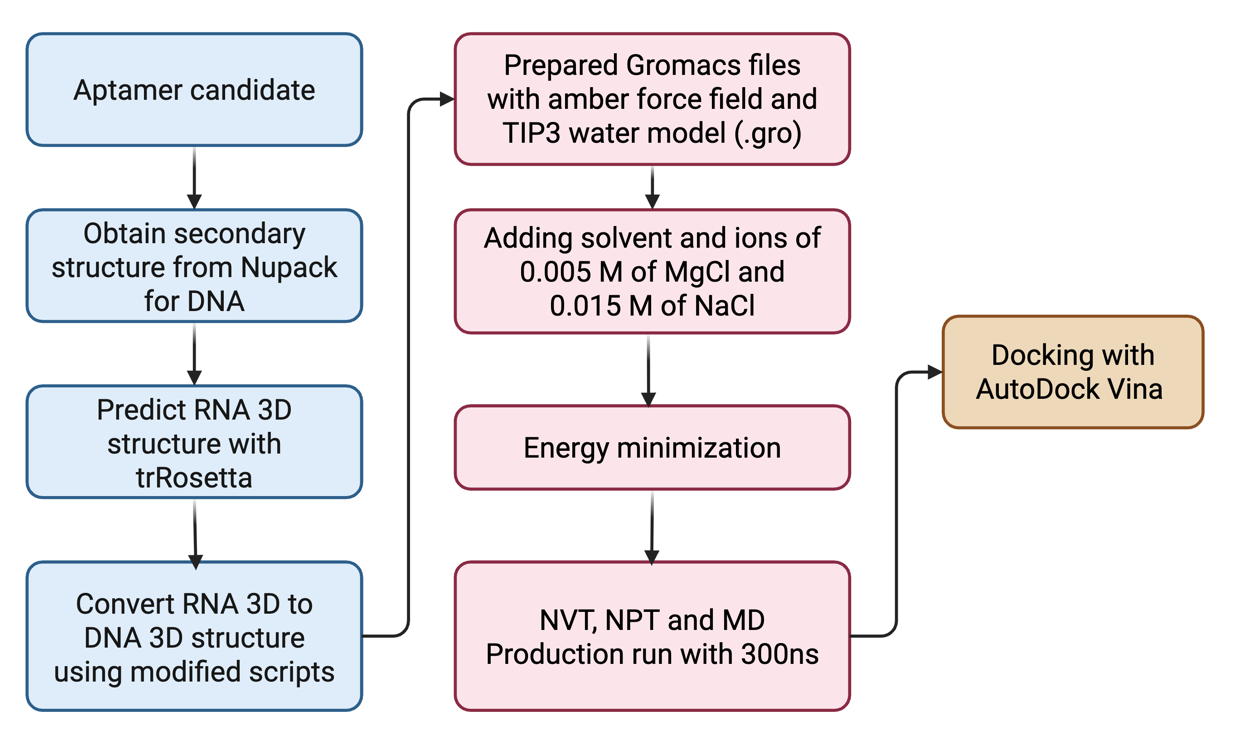


**Figure S16.** Molecular dynamics and docking pipeline in this work using GROMACS (version 2021.4) and AutoDock Vina with the HPC4 cluster at HKUST.

## Figure S17 | FRET Experiment Protocol

**
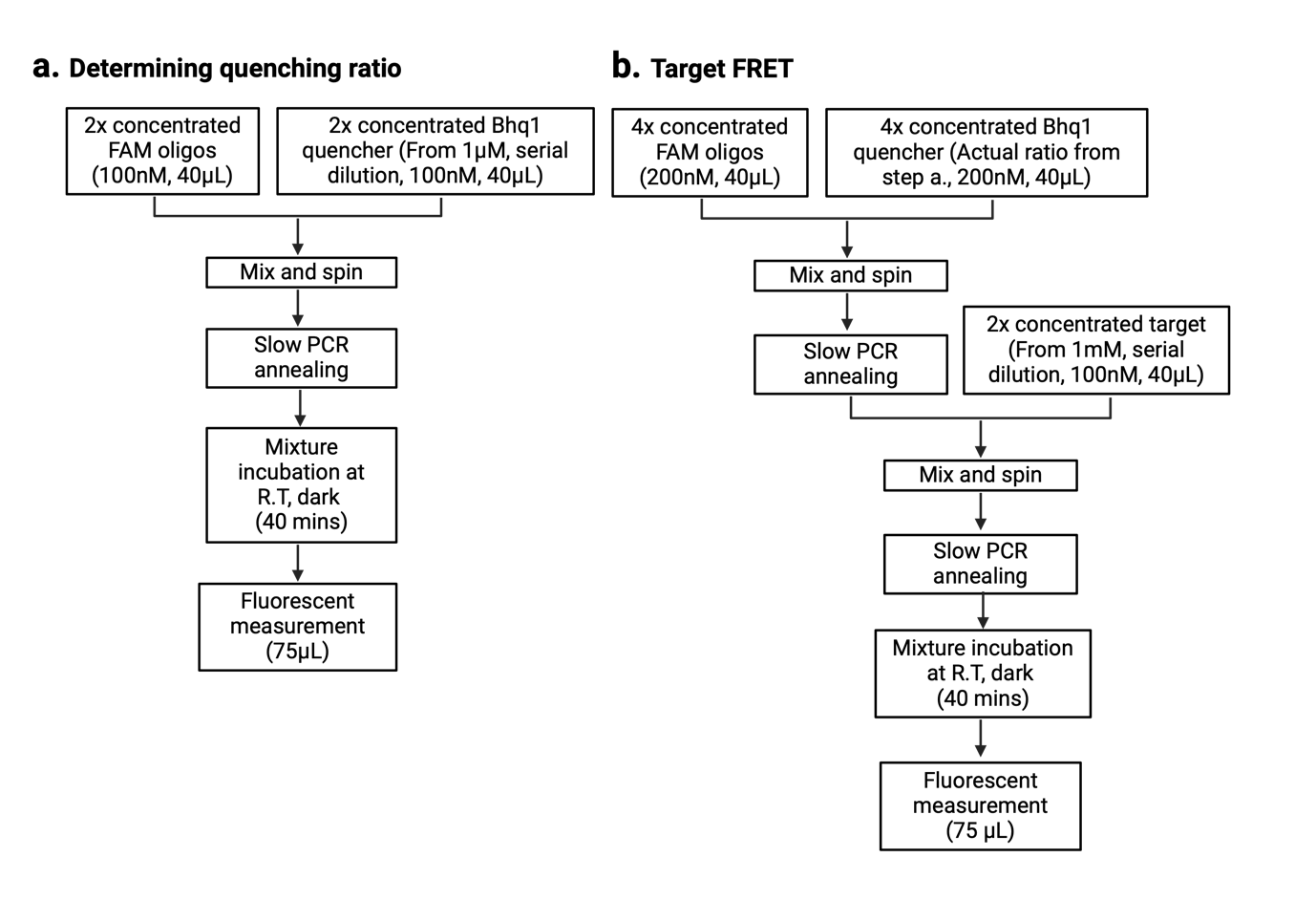
**

**Figure S17.** FRET experimental protocol, including quenching and target incubation steps. We recommend not over-incubating the target when adding it into the aptamer pool as this might first have a fluorescent reducing effect, and more importantly, the dissociation balance may be disrupted with prolonged ligand-aptamer incubation time.

## Figure S18 | FRET Results for Manual Design Library

**
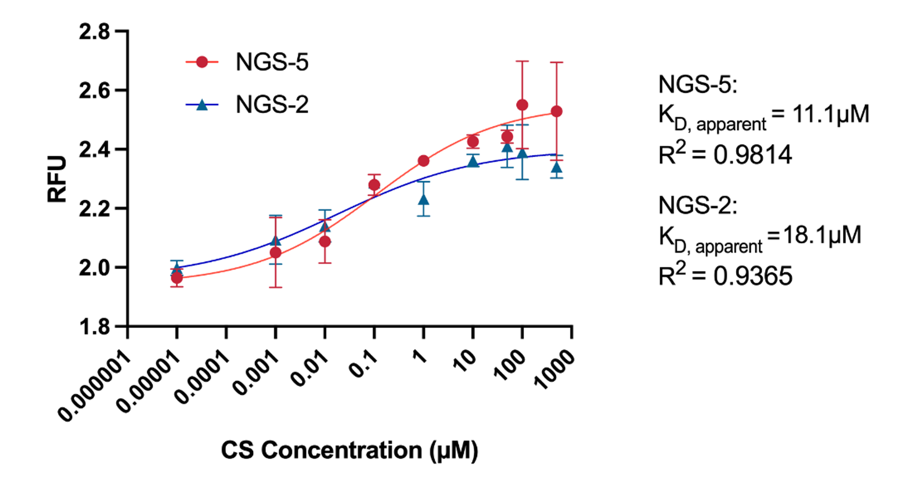
**

**Figure S18.** FRET results of the CS aptamers obtained from the manually design library SELEX experiment. Showing a comparable affinity range with the ITC results for NGS-2 (Manually designed library CS candidate obtained from round 8) and comparable affinity to the reported CS aptamer demonstrates the pre-defined library's usefulness and importance as a concept-proof experiment.

## Figure S19 | MSA of Collected Steroid Aptamers


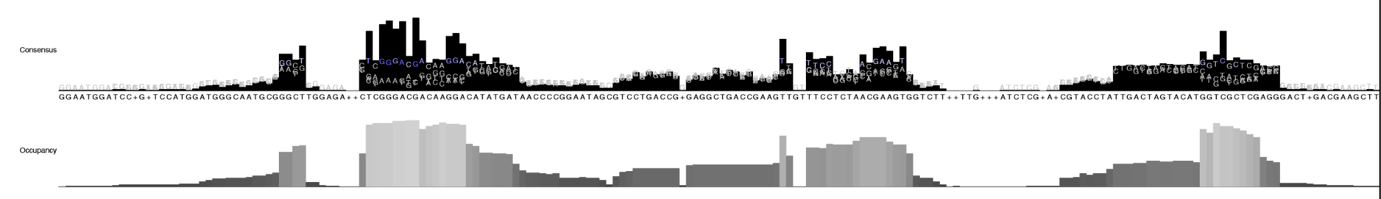


**Figure S19.** Multiple Sequence Alignment (MSA) snapshot of the 195 collected steroid aptamers. The full-quality image can be found as an attachment SI Extended Figure S19. The alignment reveals no distinct clusters, conserved motifs, or binding base patterns, indicating high variability among the sequences. This lack of identifiable structure underscores the complexity of designing a pre-defined aptamer library capable of targeting the diverse range of steroid molecules. Such variability necessitates computational approaches for efficient aptamer selection tailored to specific steroid targets.

## Figure S20 | MD comparison of truncated aptamer versus non-truncated aptamer


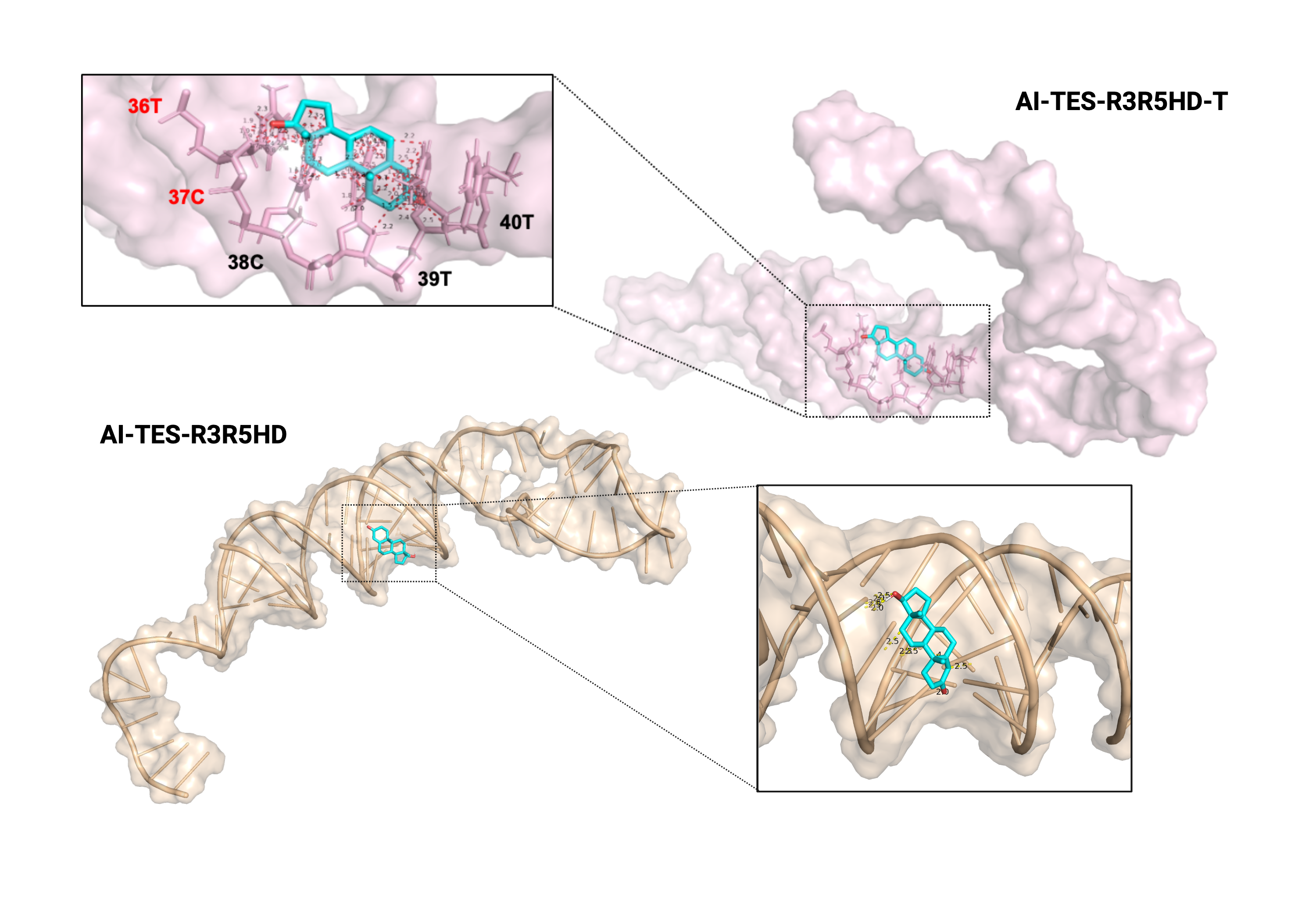


**Figure S20.**  Weaker interaction was found in the full-length aptamer (AI-TES-R3R5HD) compared to the truncated one (AI-TES-R3R5HD-T) indicated by the number of hydrogen bonds. The number of hydrogen bonds identified from the MD simulation in the truncated sequence was far more than in the full-length sequence.

## Figure S21 – S65 | ITC Results for DL-SELEX-generated Aptamers

**CS-R7MP (Binding against CS)**

**
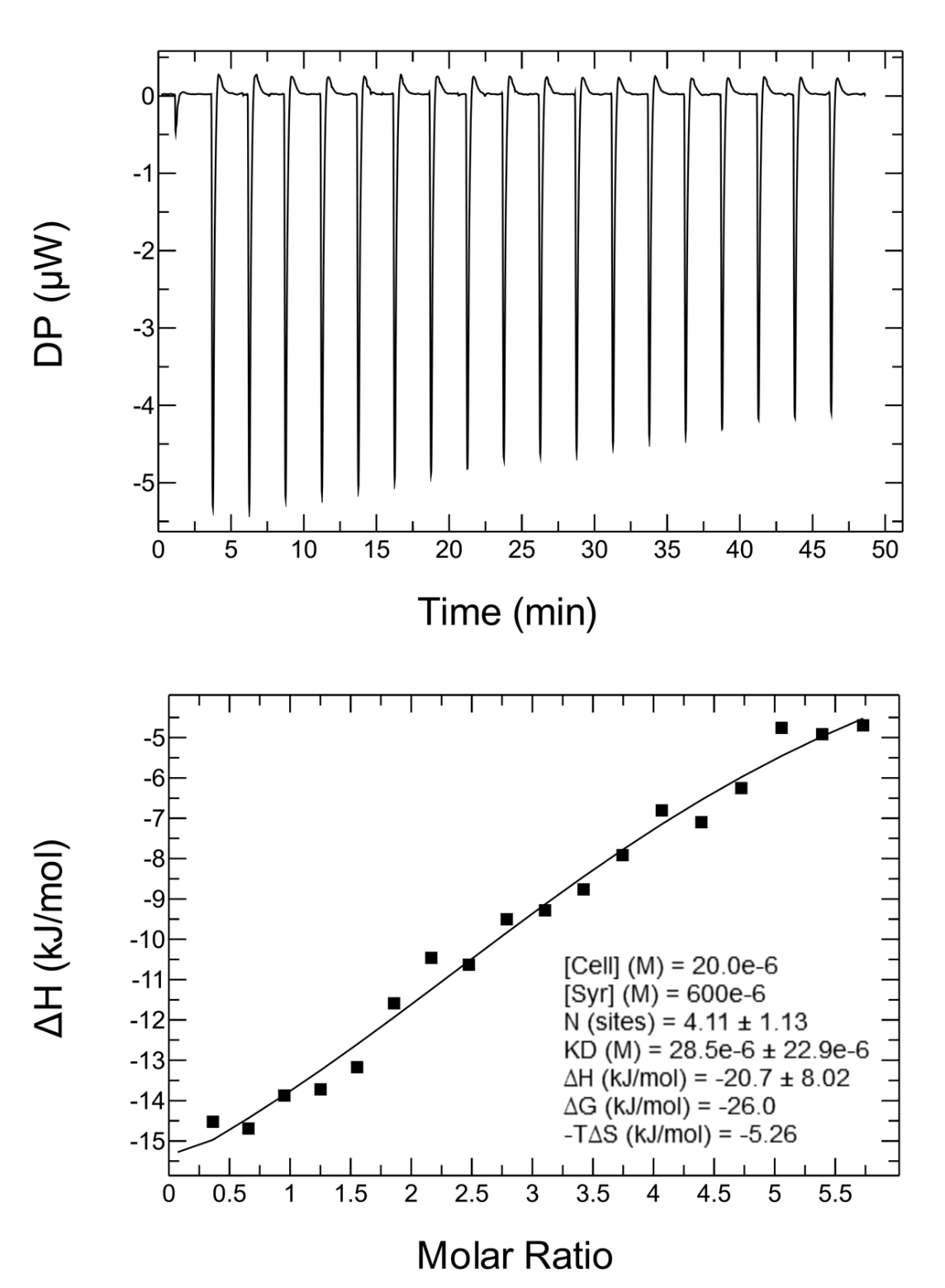
**

**Figure S21.** The affinity of CS-R7MP against CS at 25 °C. Top: raw thermogram (differential power, DP vs time) showing all injections. Bottom: integrated heats vs molar ratio with one-site fit from MicroCal PEAQ-ITC Analysis Software. Reported parameters (N, K_D, ΔH) include 95% confidence intervals from the software’s one-site fitting routine. Conditions: cell = [20µM] [aptamer], syringe = [600µM] [target], 19 injections, Heat of dilution controls (buffer→buffer, titrant→buffer, buffer→sample, vehicle→buffer/→sample) were acquired and subtracted as appropriate (detailed see Note S1). Raw traces and integrated heats for all runs are provided in CS/TES_raw_inj_heat.csv; per-run parameters are summarized in CS/TES_ITC_summary.csv.

**CS-R7MP-T (Binding against CS)**

**
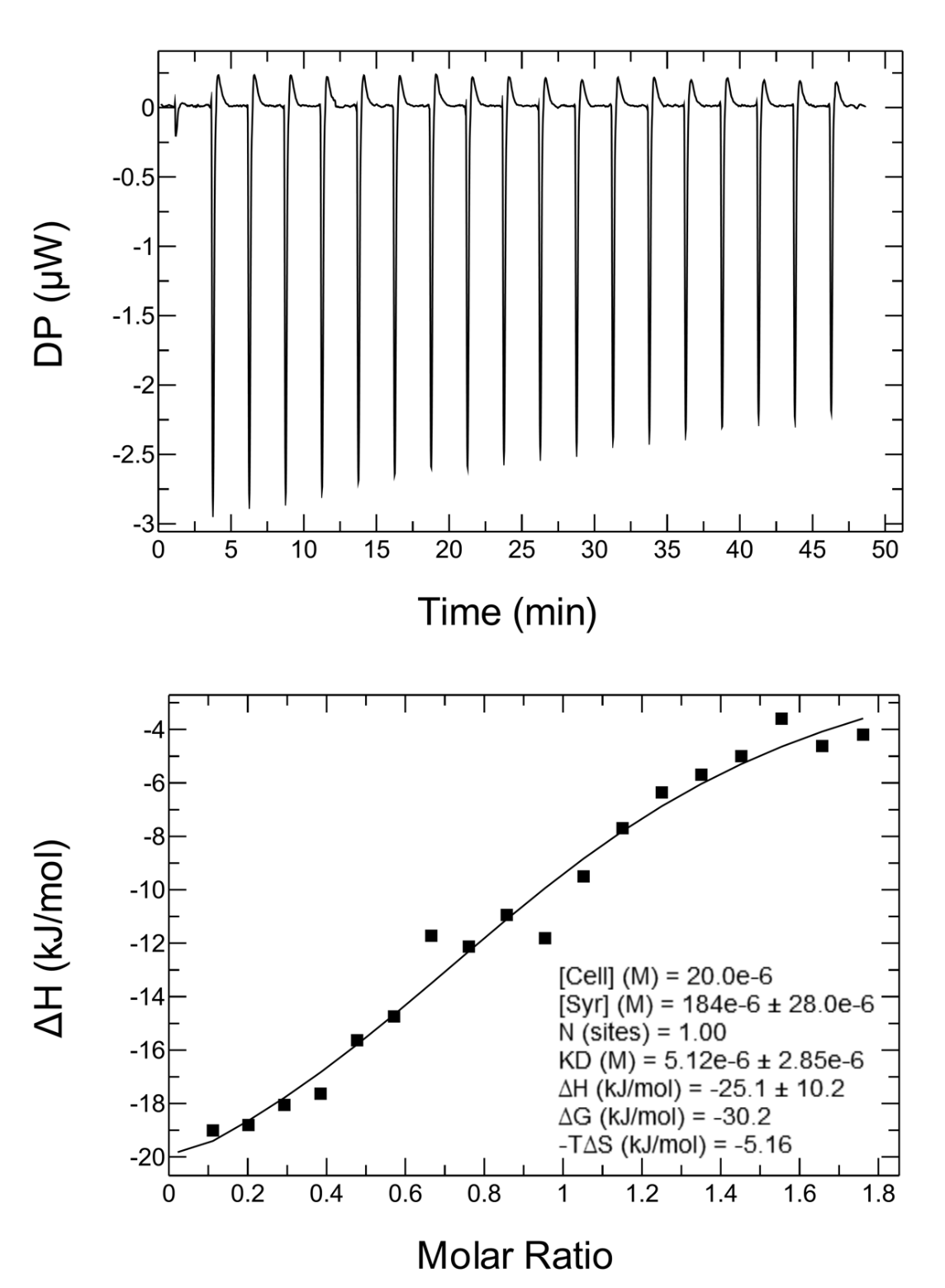
**

**Figure S22.** The affinity of CS-R7MP-T against CS from ITC experiment.

**Specificity: CS-R7MP-T (Binding against TES)**

**
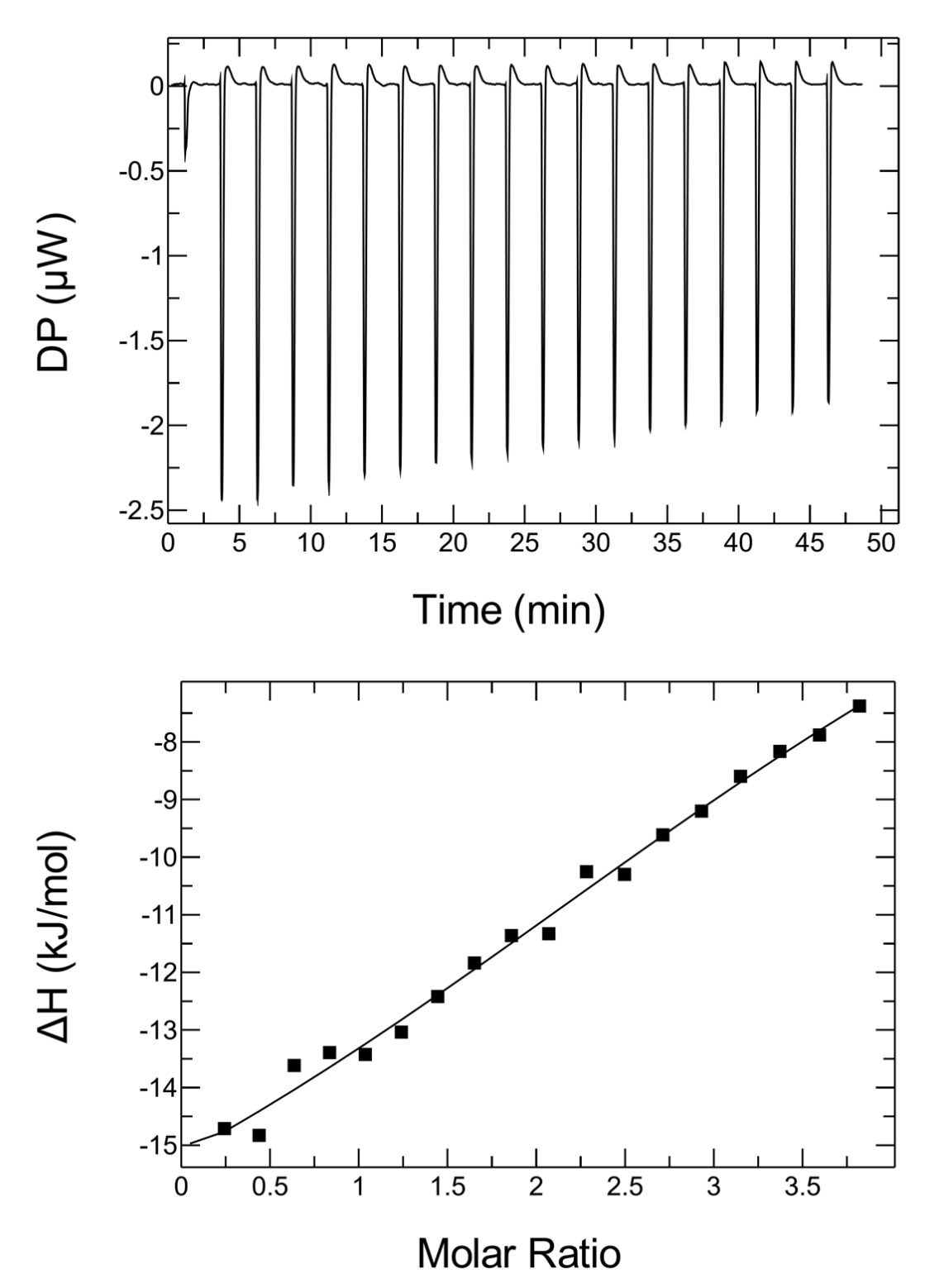
**

**Figure S23.** Specificity analysis of CS-R7MP-T against TES from ITC experiment.

**Specificity: CS-R7MP-T (Binding against BE)**

**
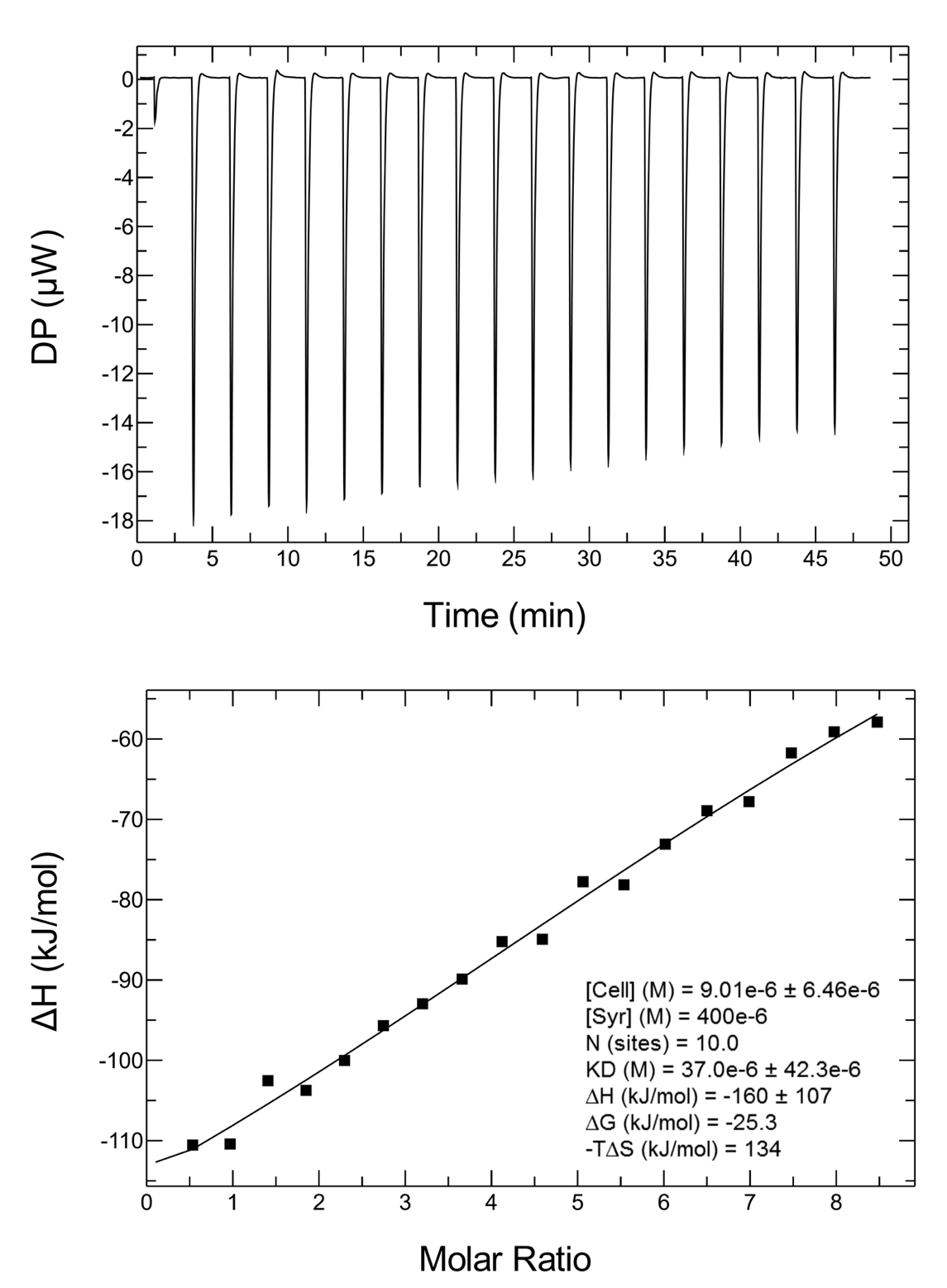
**

**Figure S24.** Specificity analysis of CS-R7MP-T against BE from ITC experiment.

**Specificity: CS-R7MP-T (Binding against DHEA)**

**
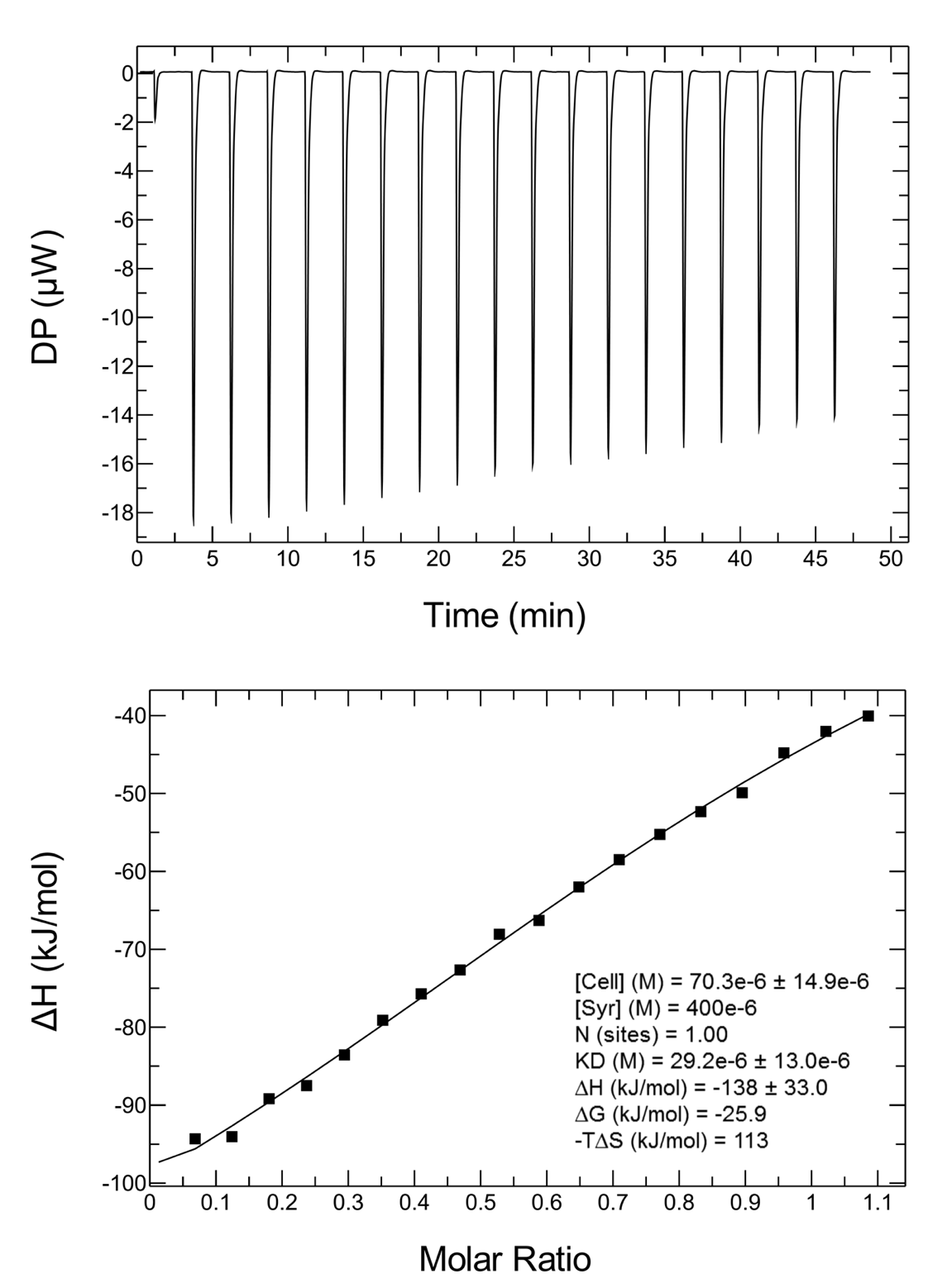
**

**Figure S25.** Specificity analysis of CS-R7MP-T against DHEA from ITC experiment.

**Specificity: CS-R7MP-T (Binding against CHO)**

**
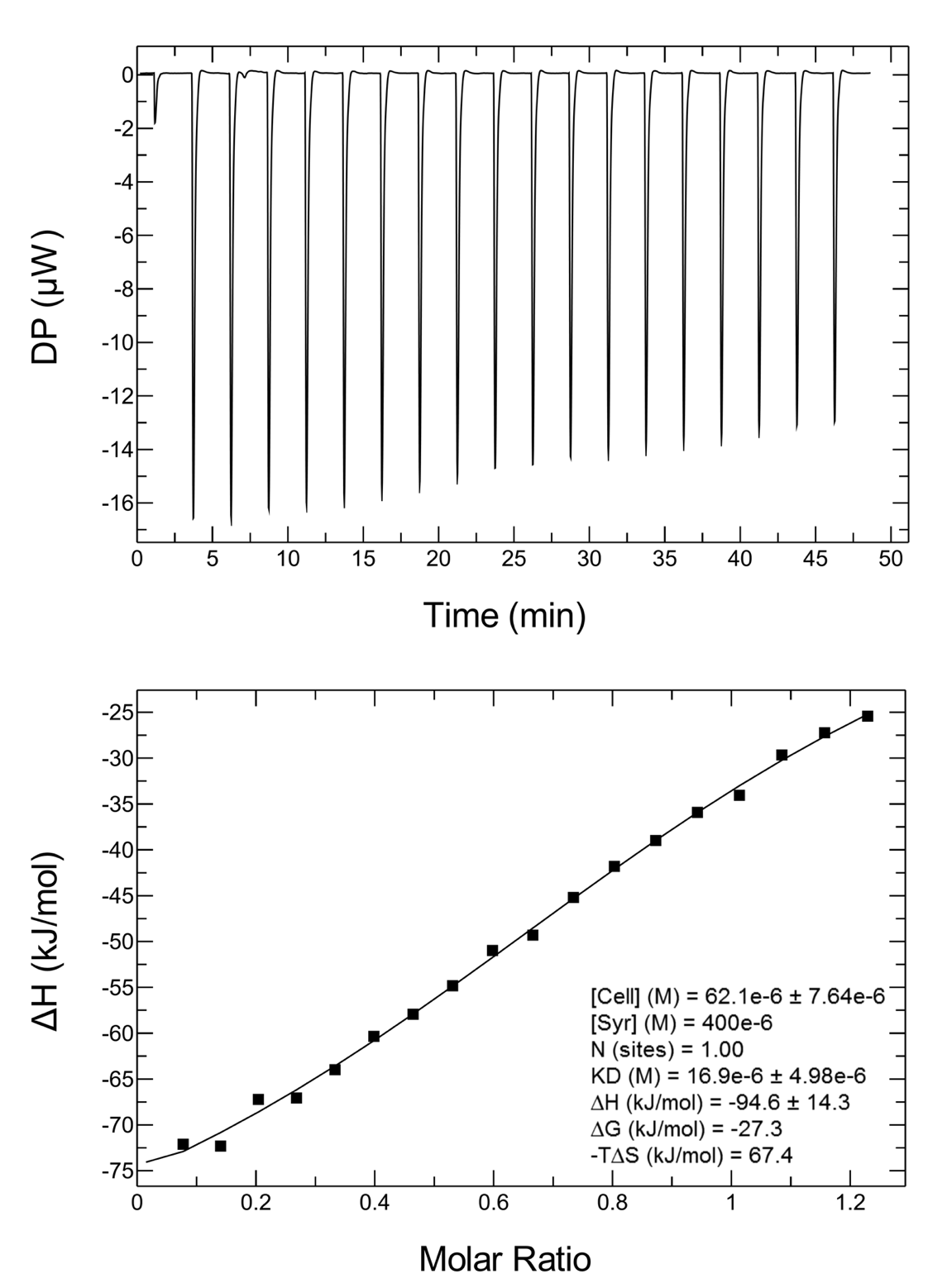
**

**Figure S26.** Specificity analysis of CS-R7MP-T against CHO from ITC experiment.

**Specificity: CS-R7MP-T (Binding against PRO)**

**
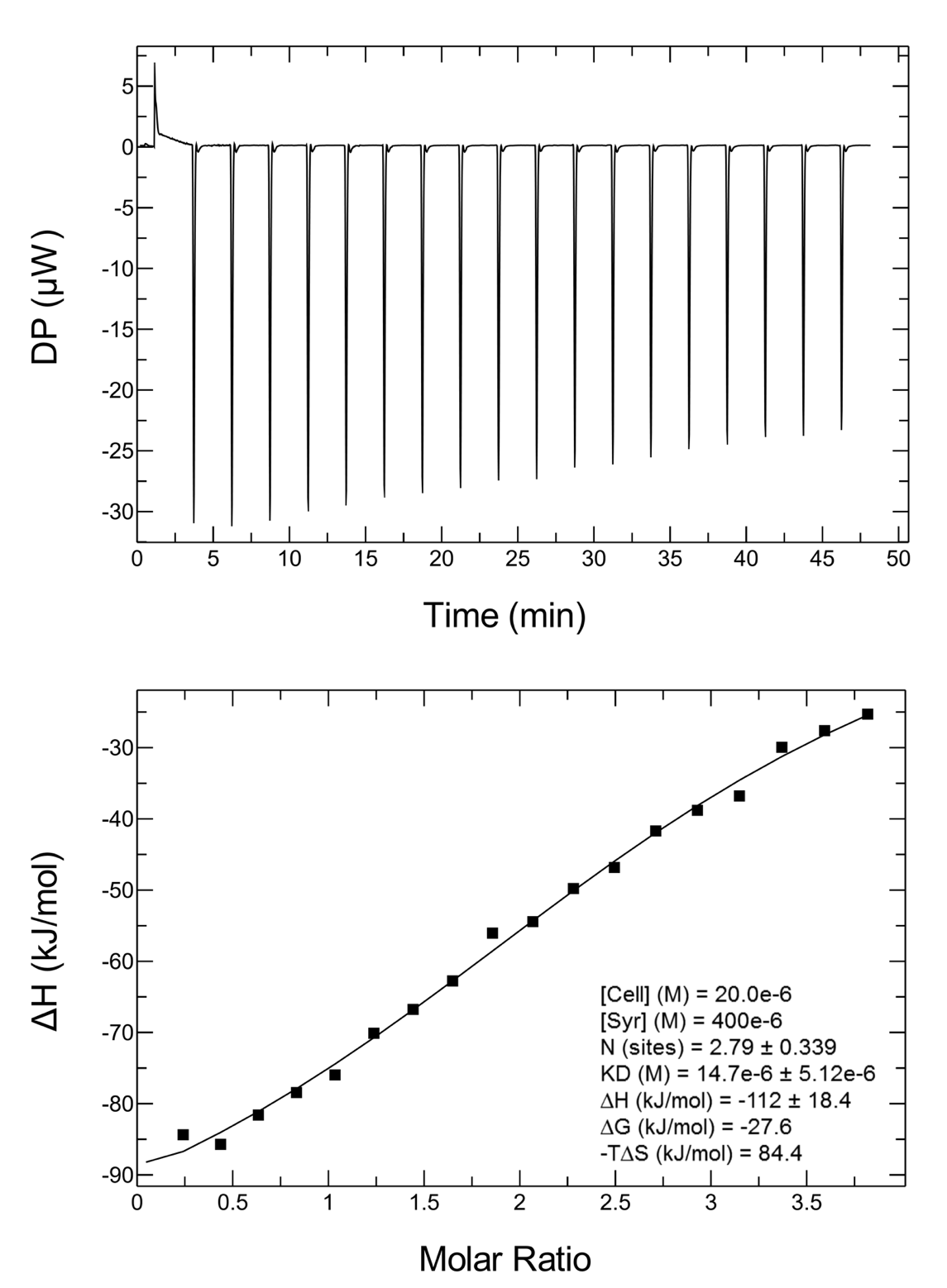
**

**Figure S27.** Specificity analysis of CS-R7MP-T against PRO from ITC experiment.

**CS-R5MP (Binding against CS)**

**
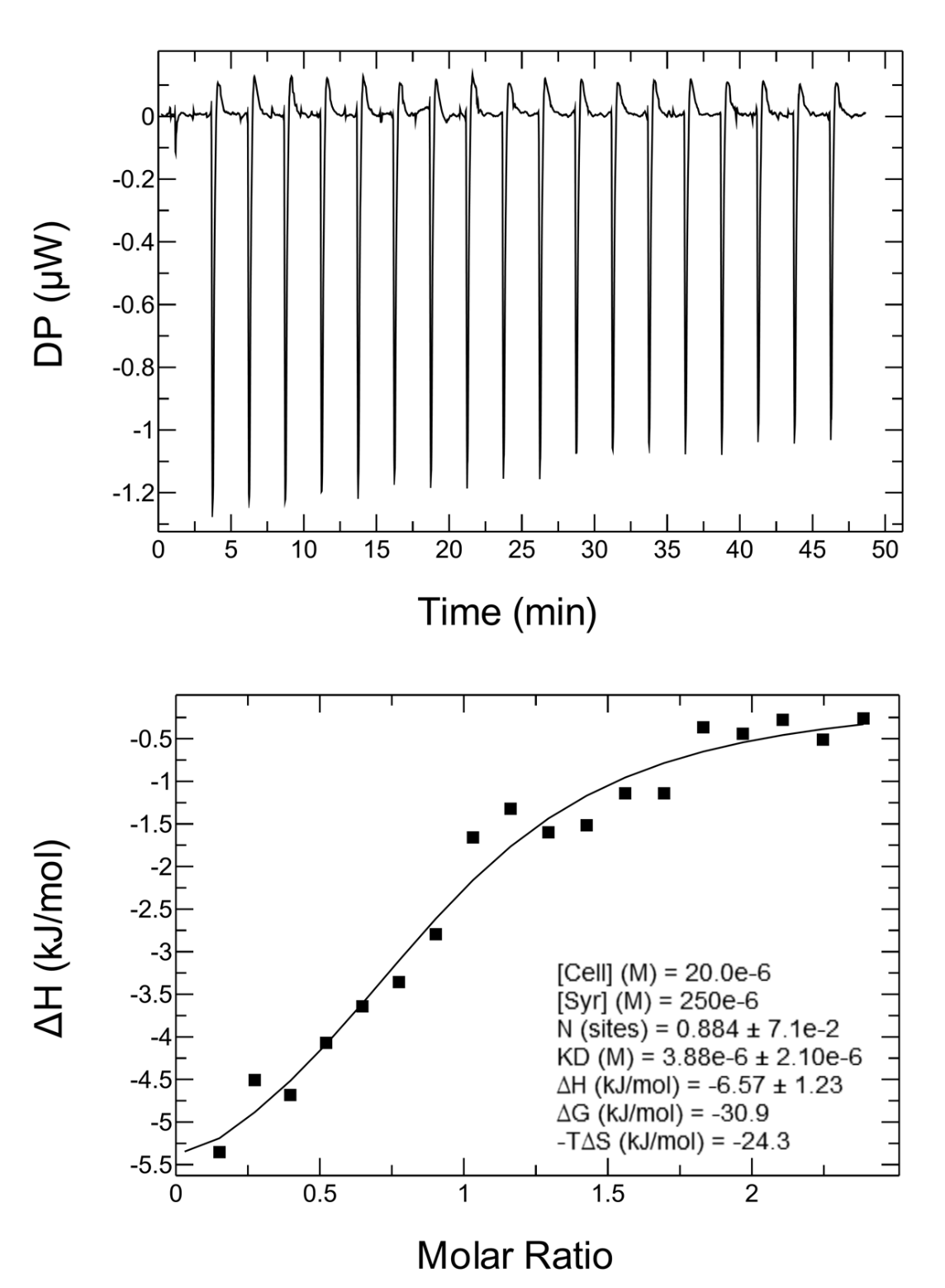
**

**Figure S28.** The affinity of CS-R5MP against CS from ITC experiment.

**CS-R5MP-T (Binding against CS)**

**
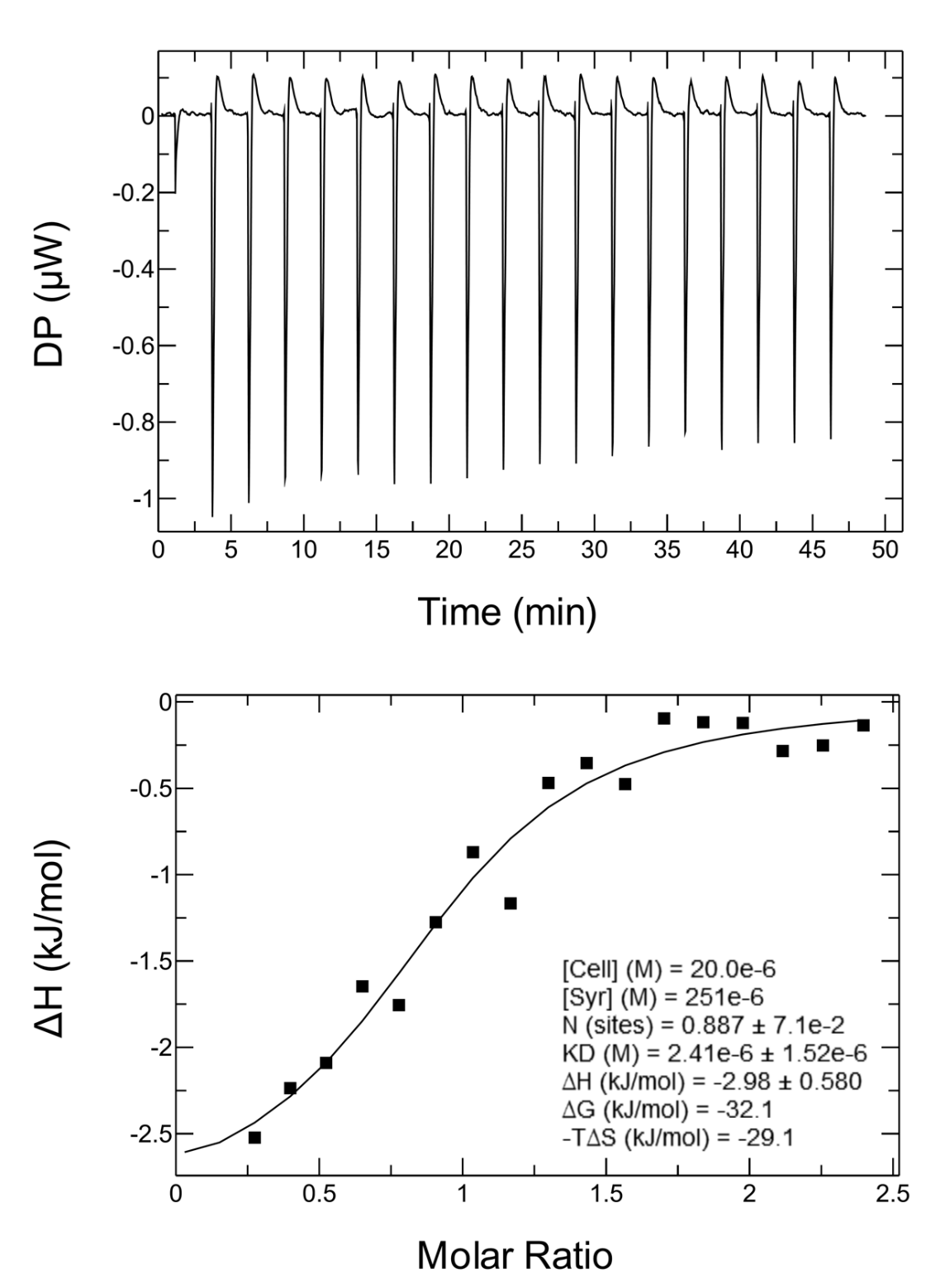
**

**Figure S29.** The affinity of CS-R5MP-T against CS from ITC experiment.

**Specificity: CS-R5MP-T (Binding against TES)**

**
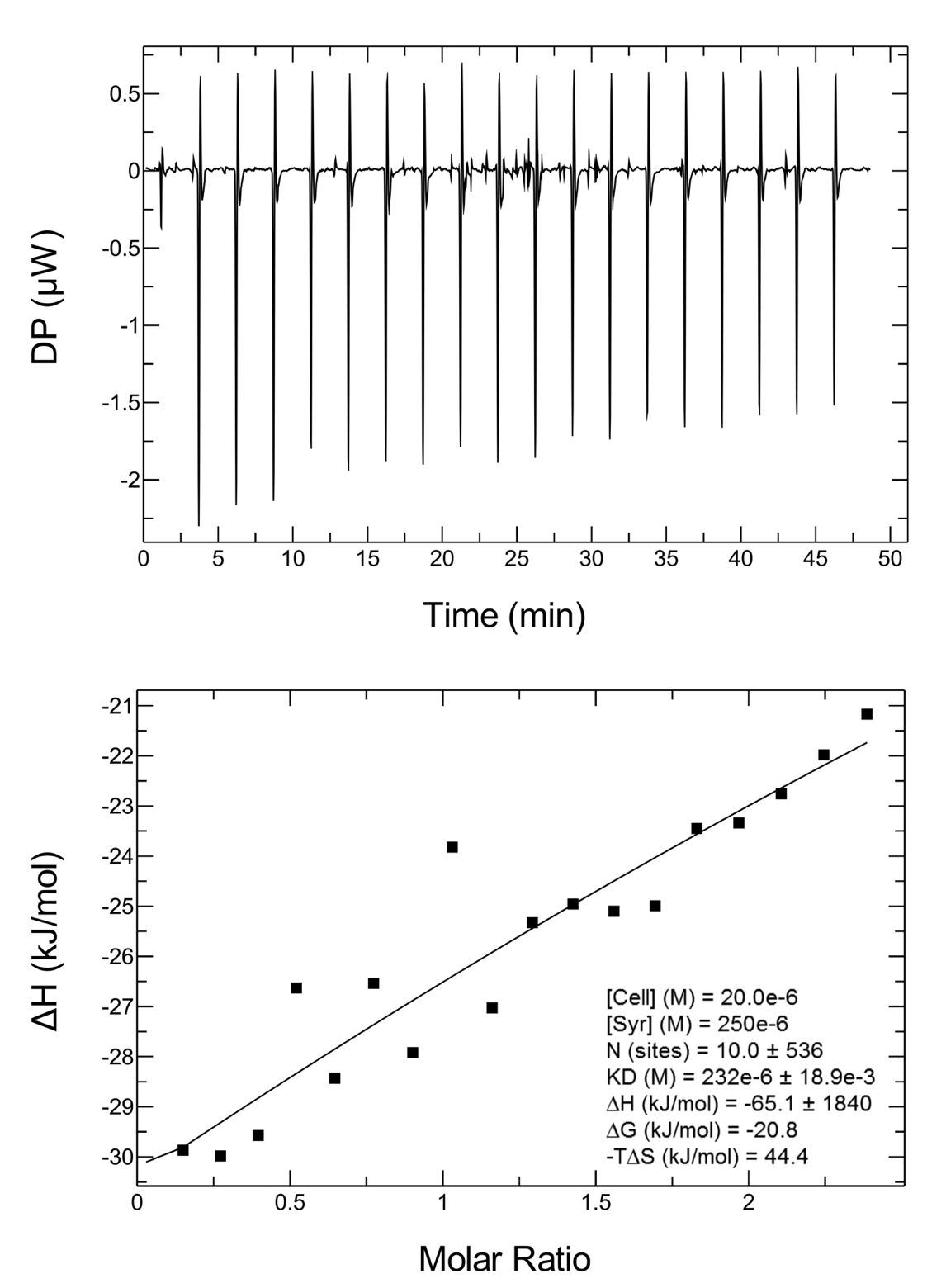
**

**Figure S30.** Specificity analysis of CS-R5MP-T against TES from ITC experiment.

**Specificity: CS-R5MP-T (Binding against BE)**

**
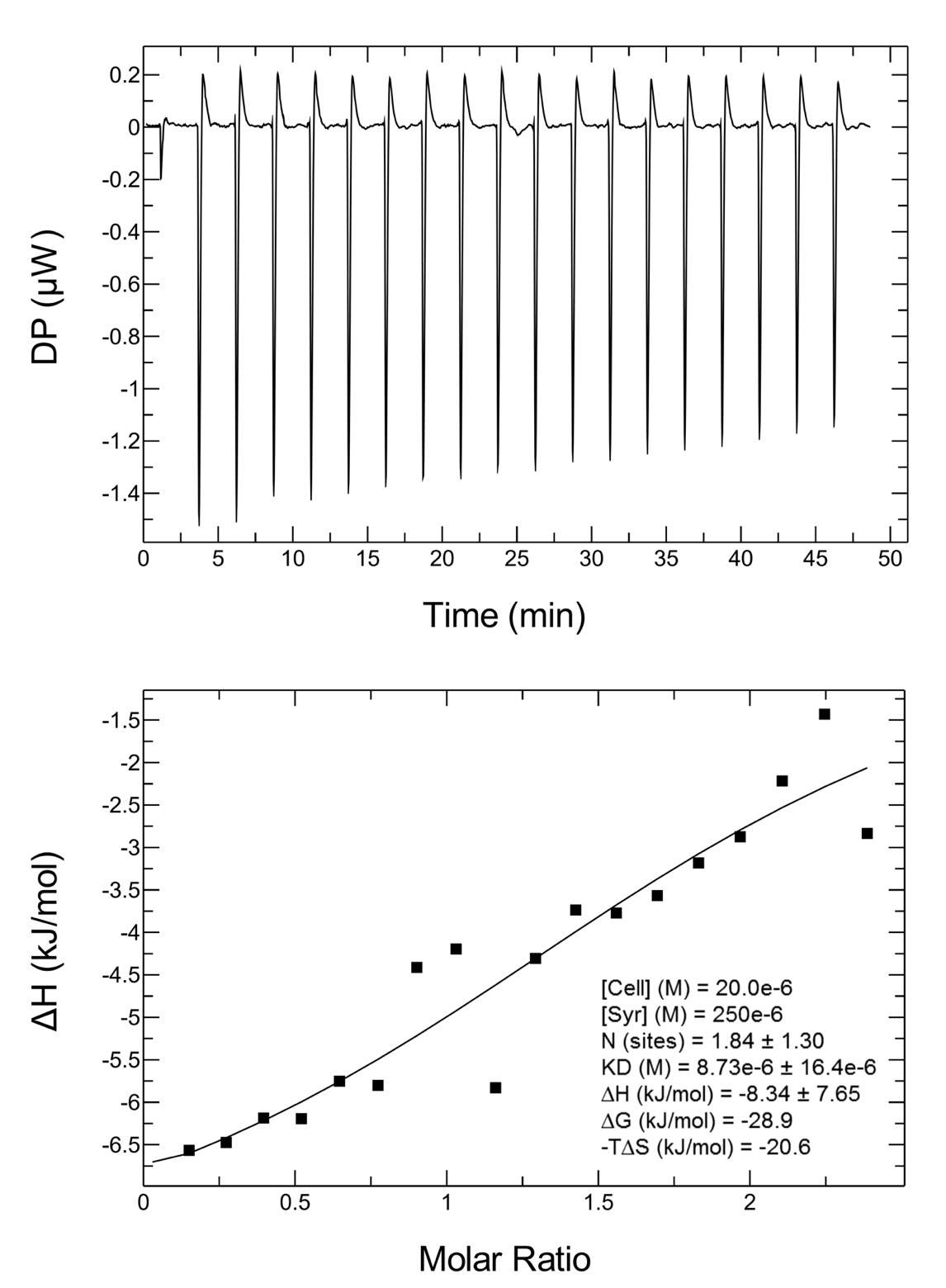
**

**Figure S31.** Specificity analysis of CS-R5MP-T against BE from ITC experiment.

**Specificity: CS-R5MP-T (Binding against DHEA)**

**
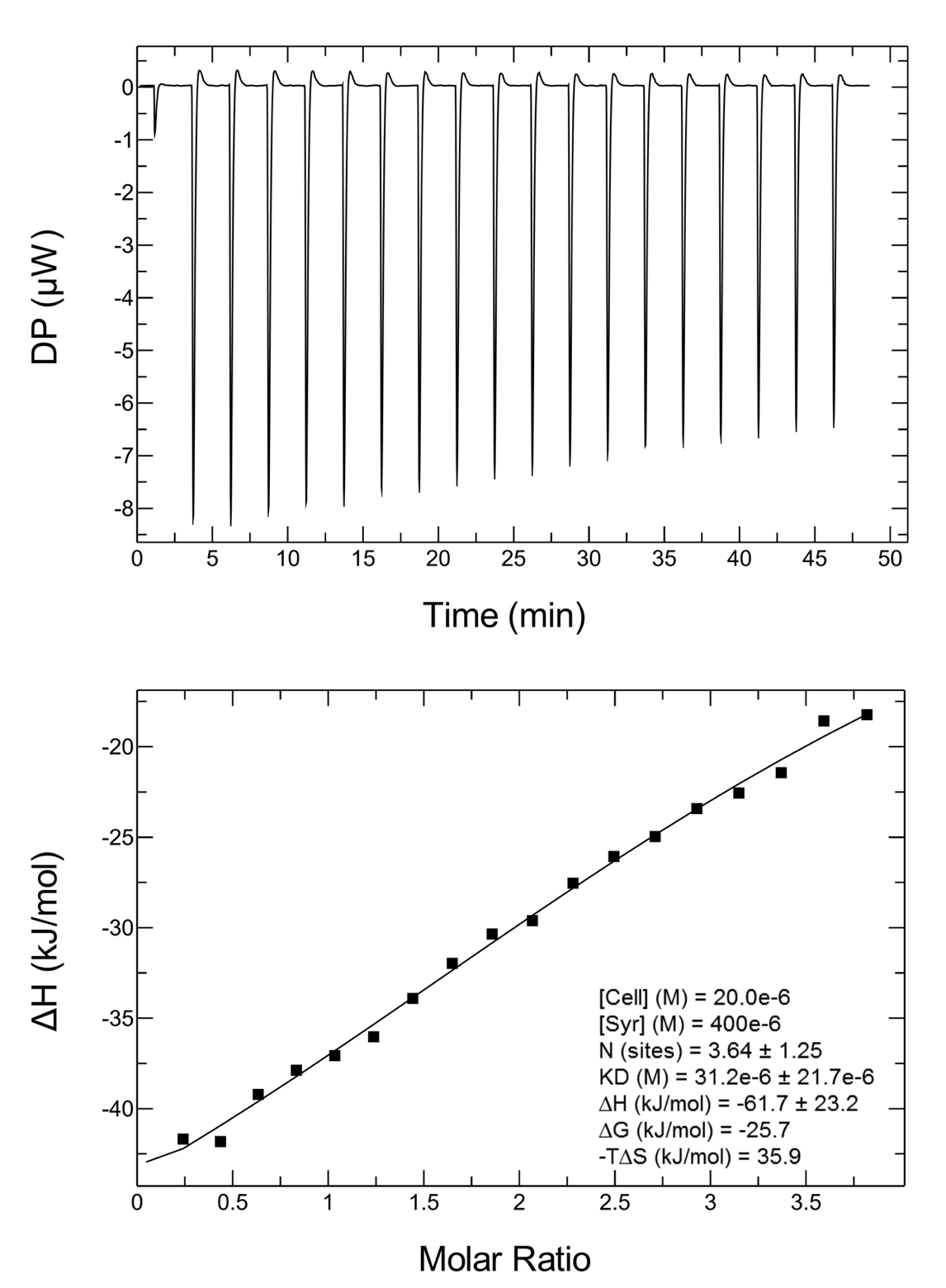
**

**Figure S32.** Specificity analysis of CS-R5MP-T against DHEA from ITC experiment.

**Specificity: CS-R5MP-T (Binding against CHO)**

**
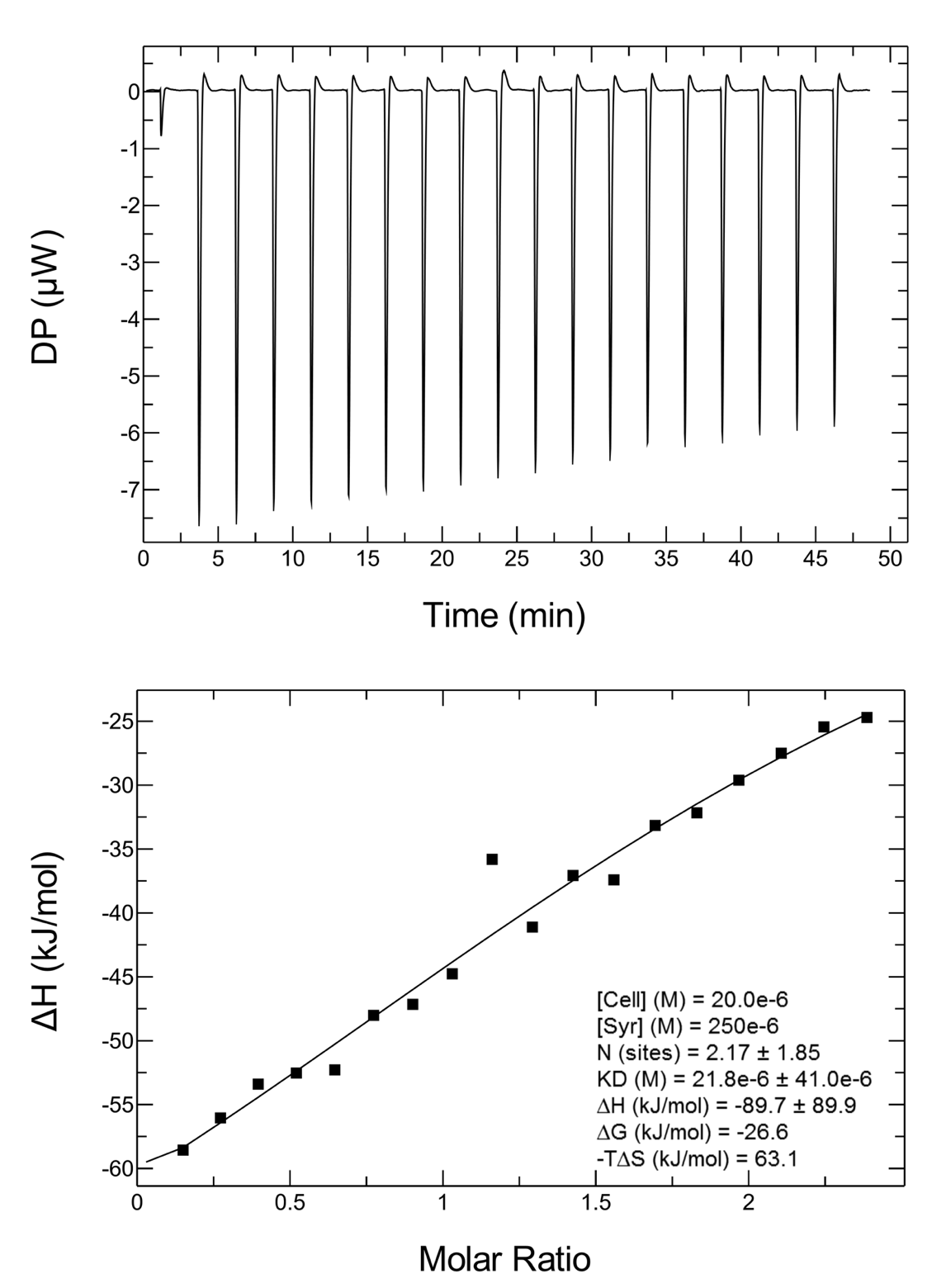
**

**Figure S33.** Specificity analysis of CS-R5MP-T against CHO from ITC experiment.

**Specificity: CS-R5MP-T (Binding against PRO)**

**
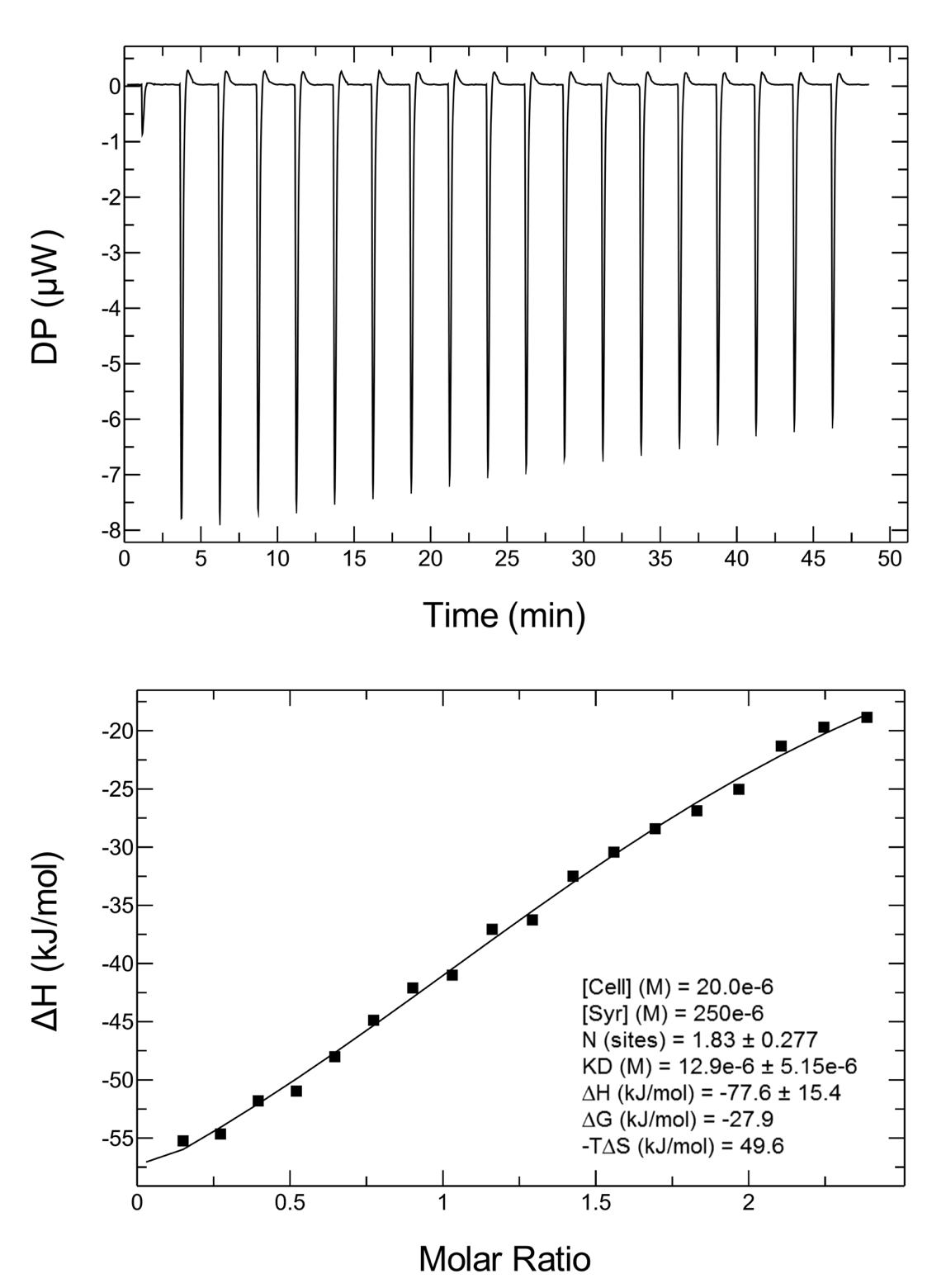
**

**Figure S34.** Specificity analysis of CS-R5MP-T against PRO from ITC experiment.

**AI-CS-R5CC (Binding against CS)**

**
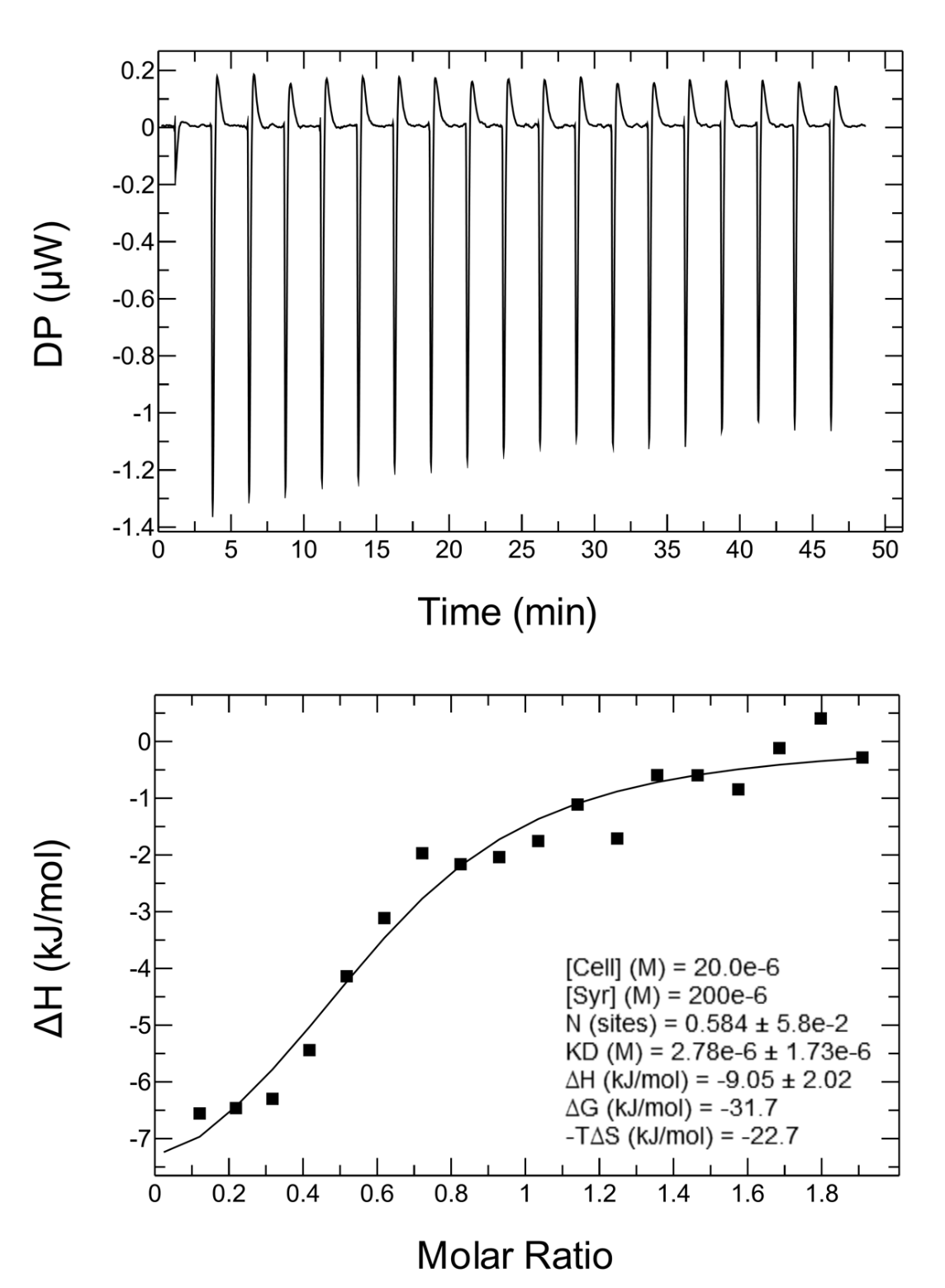
**

**Figure S35.** The affinity of AI-CS-R5CC against CS from ITC experiment.

**AI-CS-R5CC-T (Binding against CS)**

**
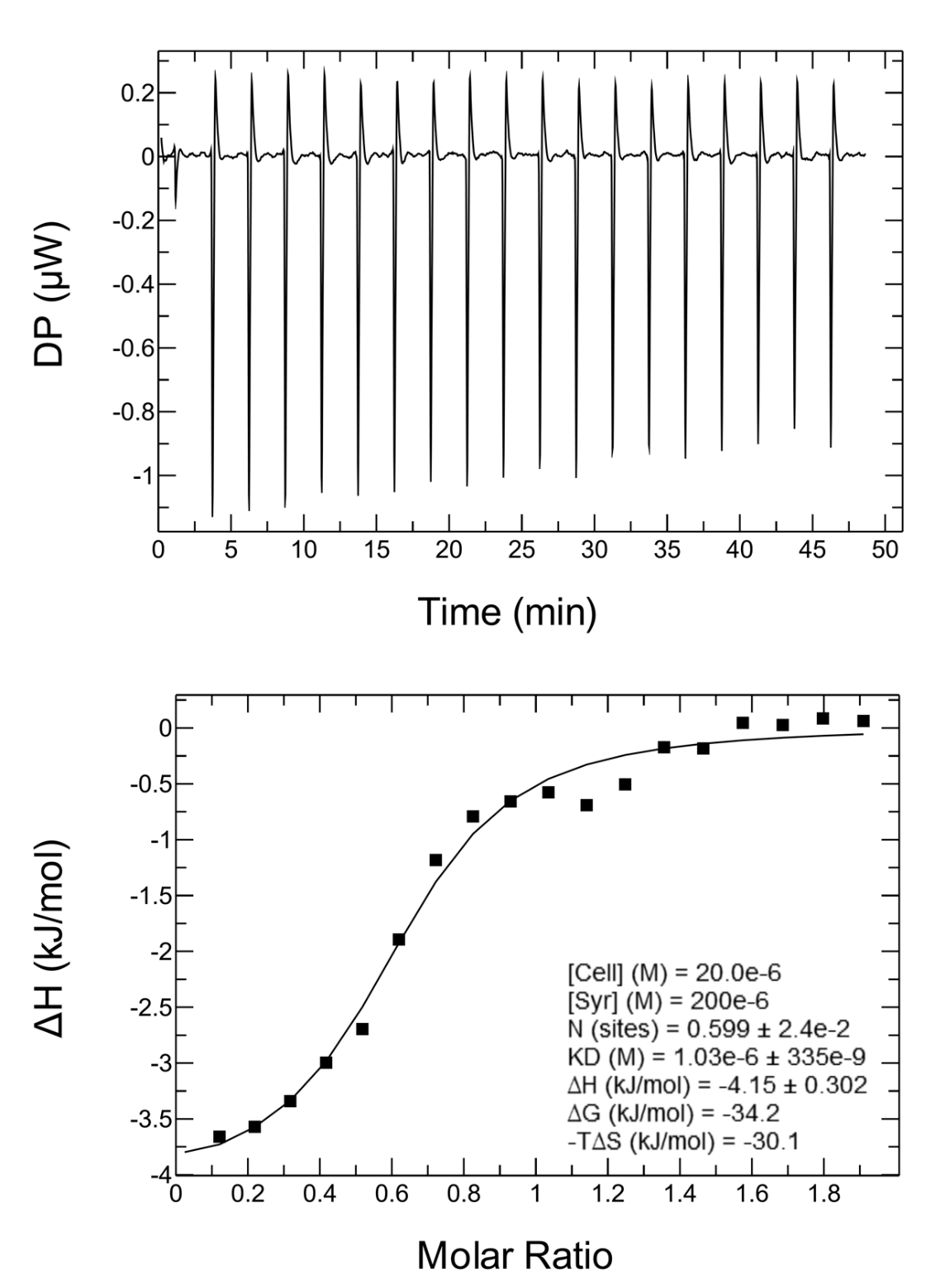
**

**Figure S36.** The affinity of AI-CS-R5CC-T against CS from ITC experiment.

**Specificity: AI-CS-R5CC-T (Binding against TES)**

**
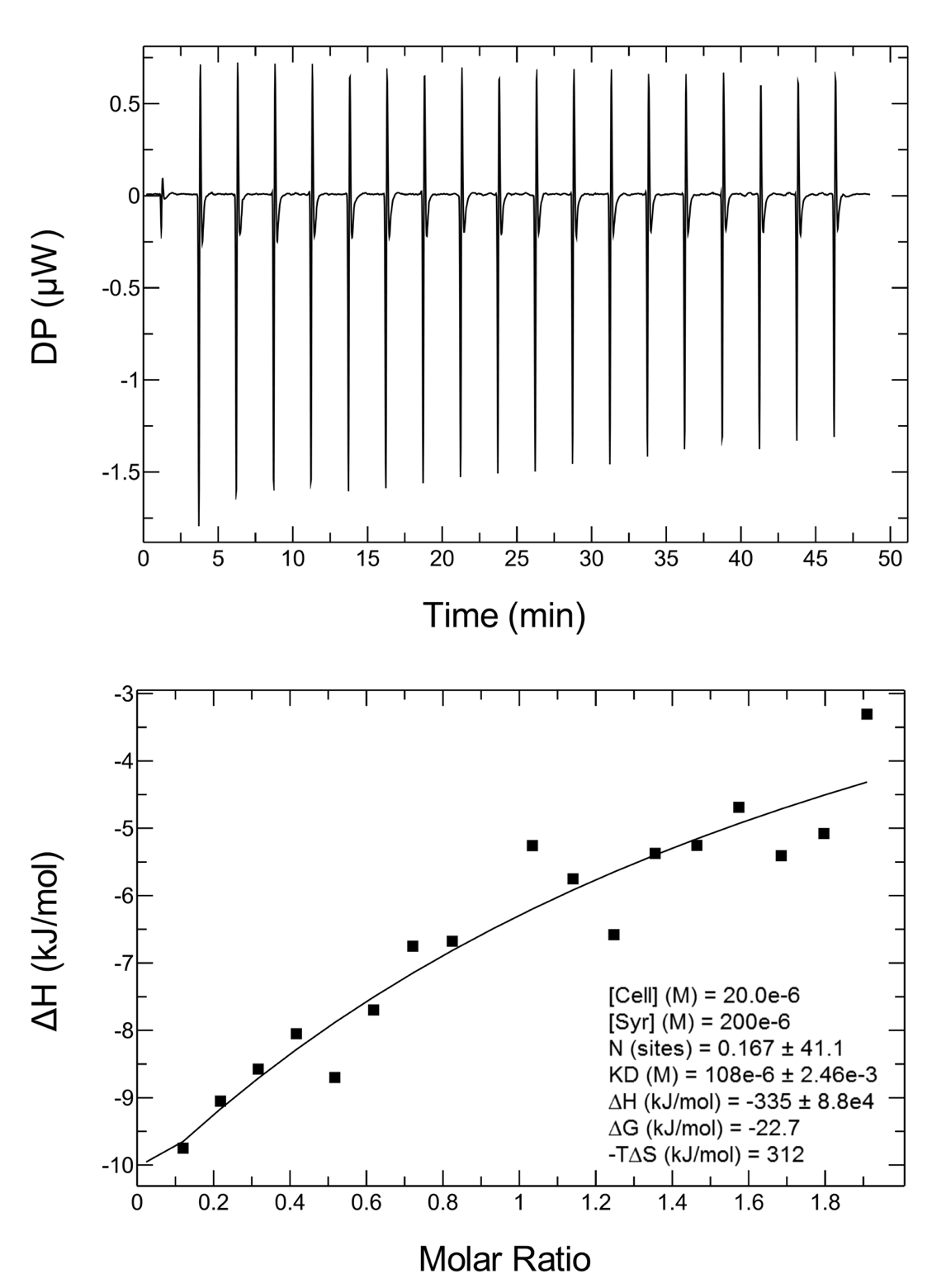
**

**Figure S37.** Specificity analysis of AI-CS-R5CC-T against TES from ITC experiment.

**Specificity: AI-CS-R5CC-T (Binding against BE)**

**
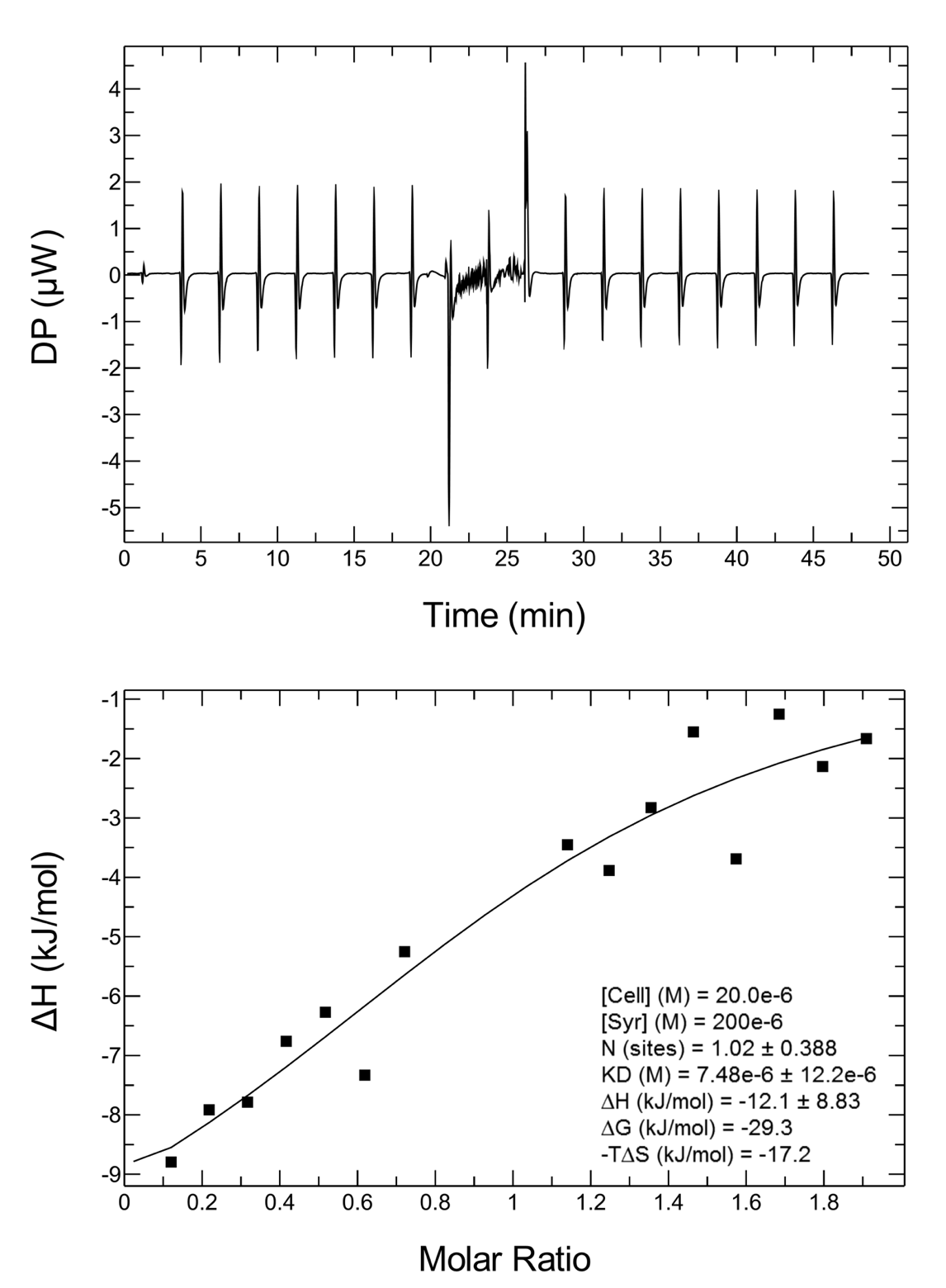
**

**Figure S38.** Specificity analysis of AI-CS-R5CC-T against BE from ITC experiment.

**Specificity: AI-CS-R5CC-T (Binding against DHEA)**

**
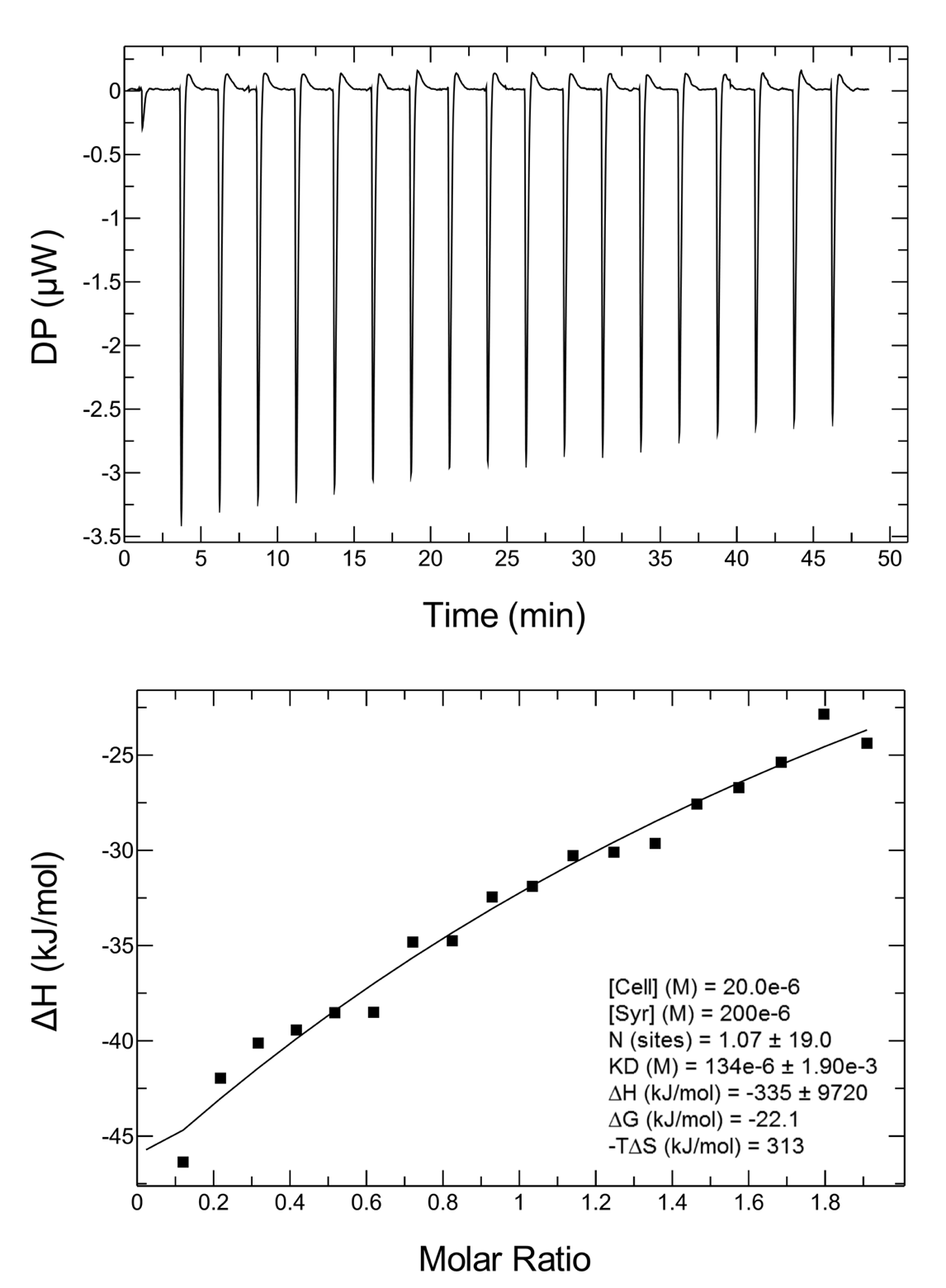
**

**Figure S39.** Specificity analysis of AI-CS-R5CC-T against DHEA from ITC experiment.

**Specificity: AI-CS-R5CC-T (Binding against CHO)**

**
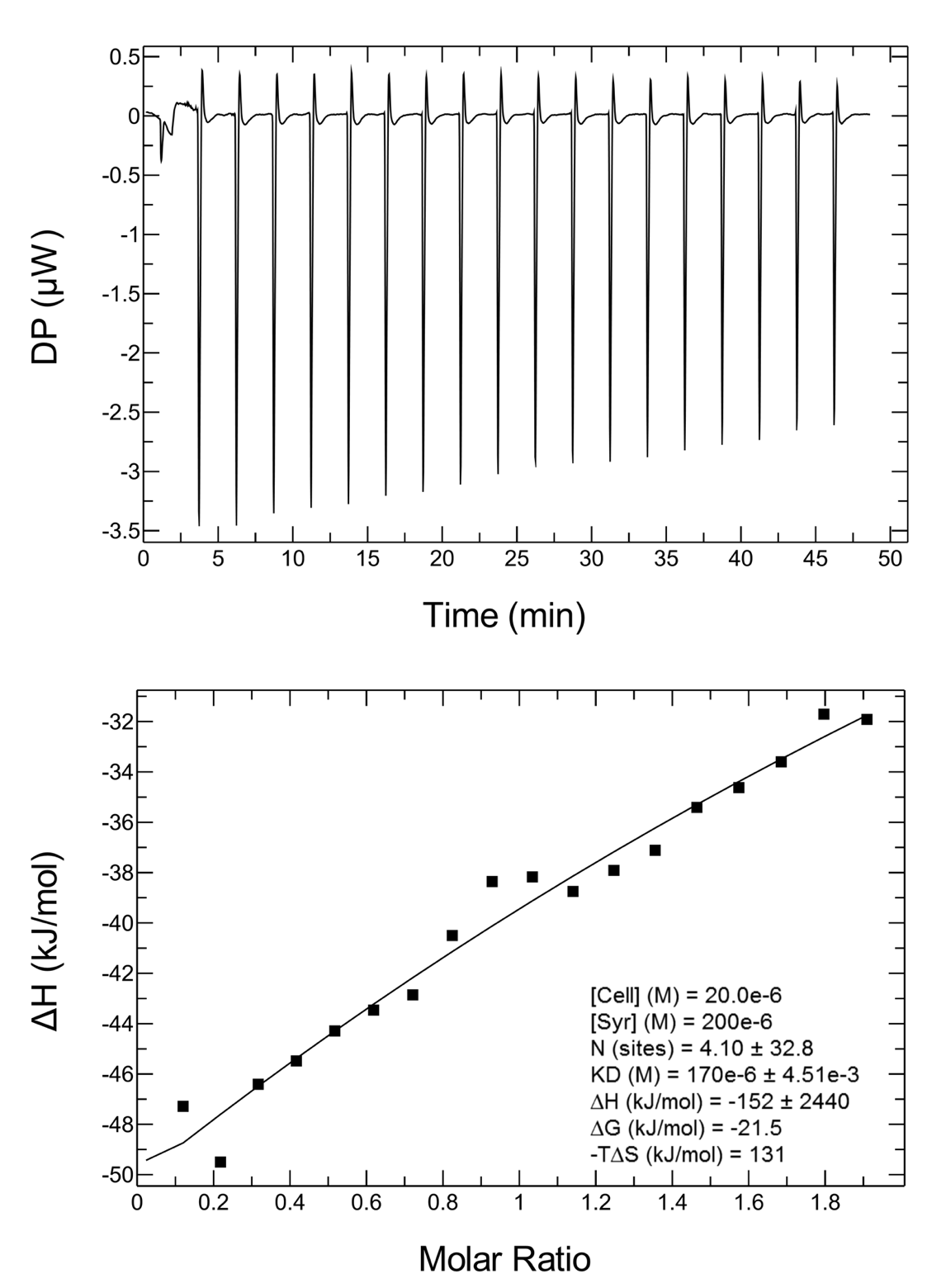
**

**Figure S40.** Specificity analysis of AI-CS-R5CC-T against CHO from ITC experiment.

**Specificity: AI-CS-R5CC-T (Binding against PRO)**

**
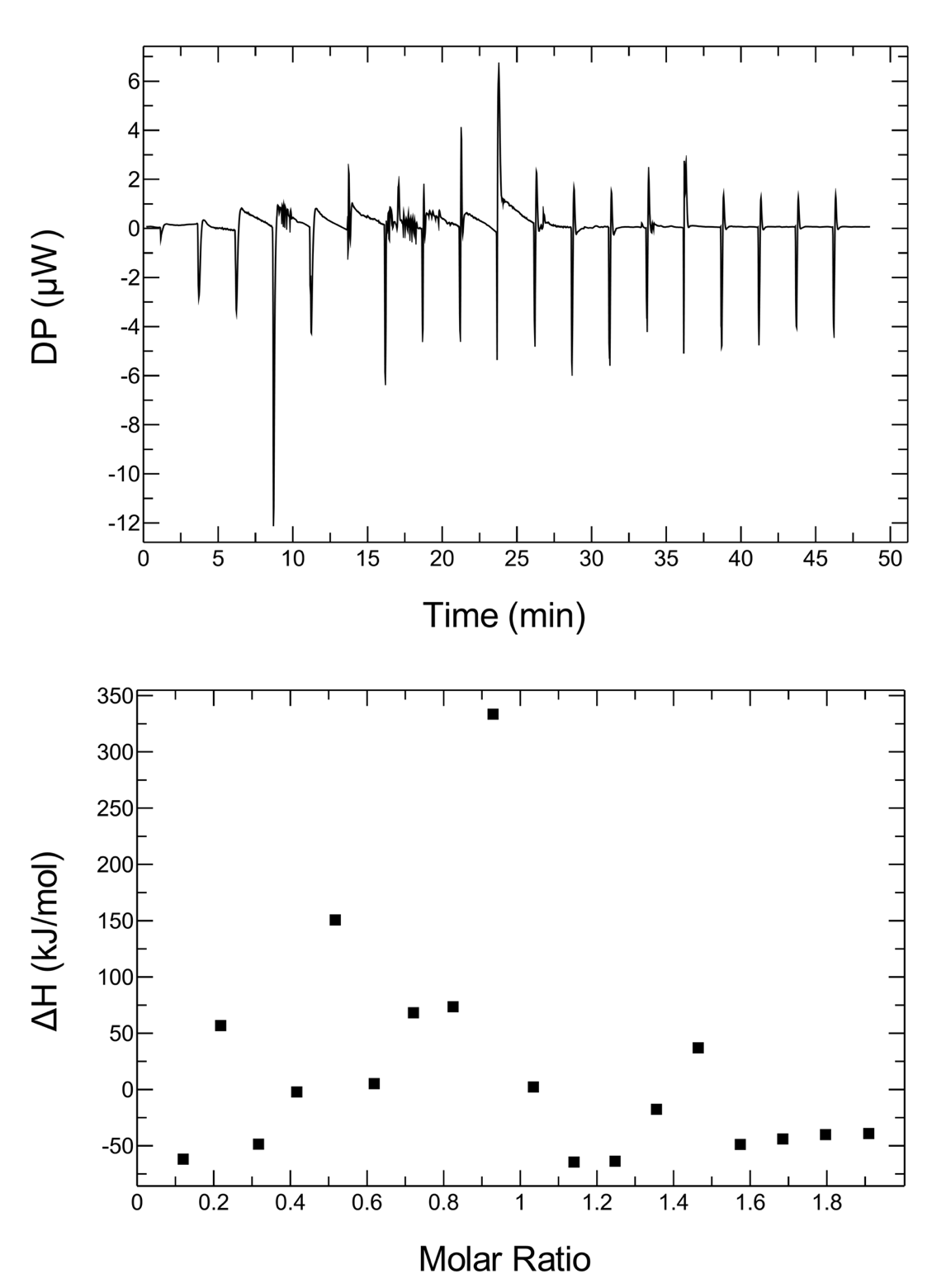
**

**Figure S41.** Specificity analysis of AI-CS-R5CC-T against PRO from ITC experiment.

**AI-CS-R3CC (Binding against CS)**

**
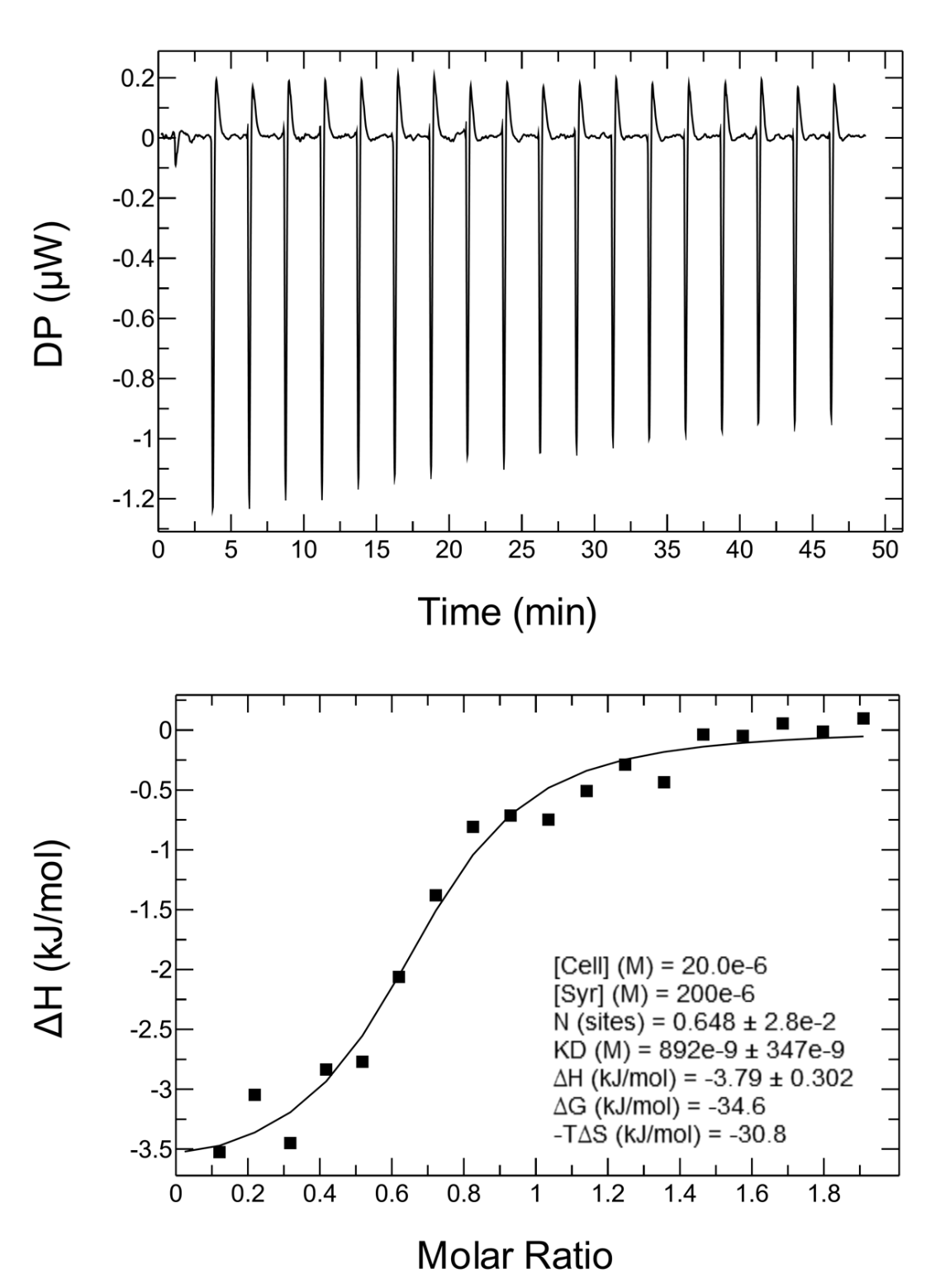
**

**Figure S42.** The affinity of AI-CS-R3CC against CS from ITC experiment.

**AI-CS-R3CC-T (Binding against CS)**

**
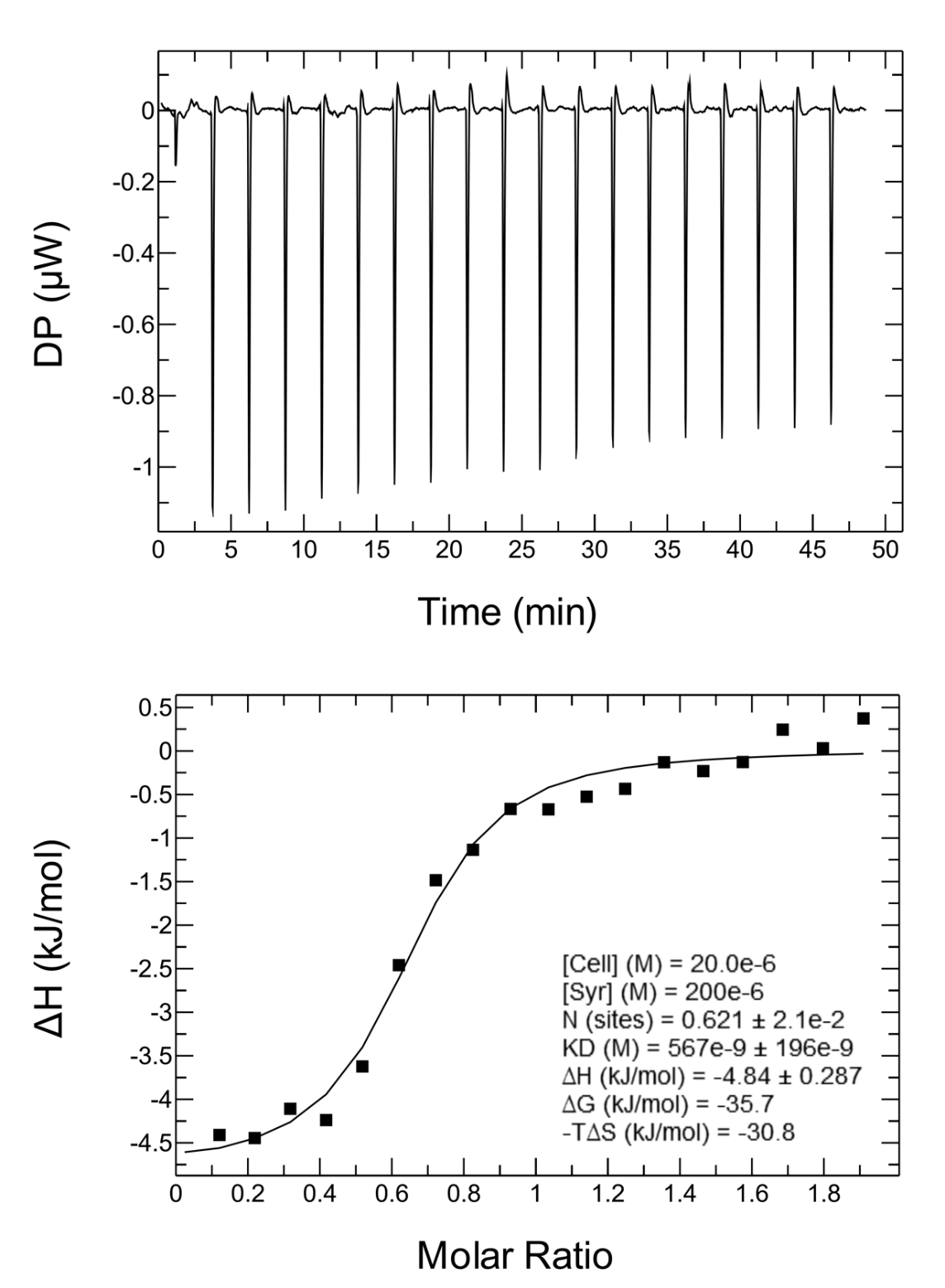
**

**Figure S43.** The affinity of AI-CS-R3CC-T against CS from ITC experiment.

**Specificity: AI-CS-R3CC-T (Binding against TES)**

**
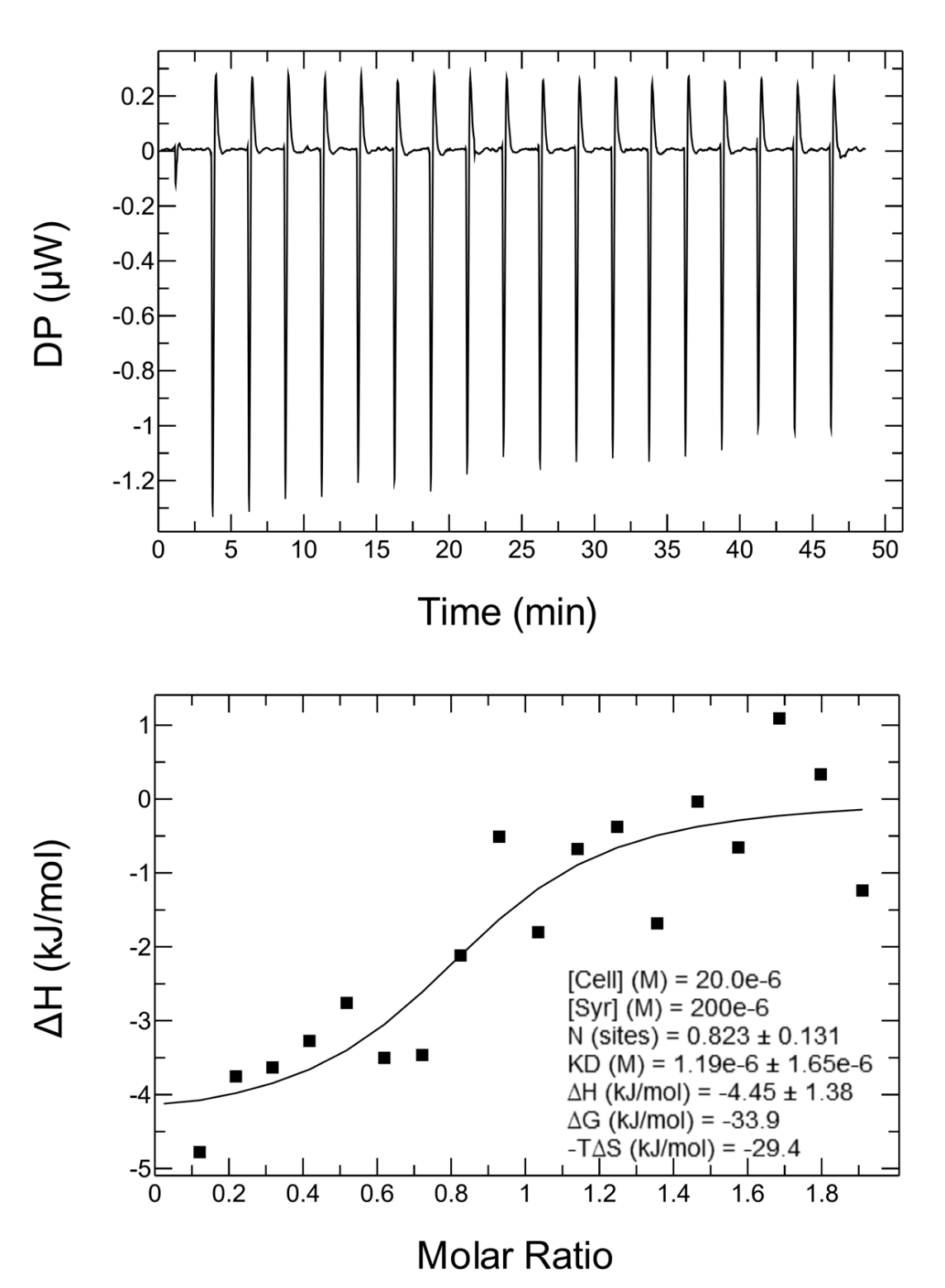
**

**Figure S44.** Specificity analysis of AI-CS-R3CC-T against TES from ITC experiment.

**Specificity: AI-CS-R3CC-T (Binding against BE)**

**
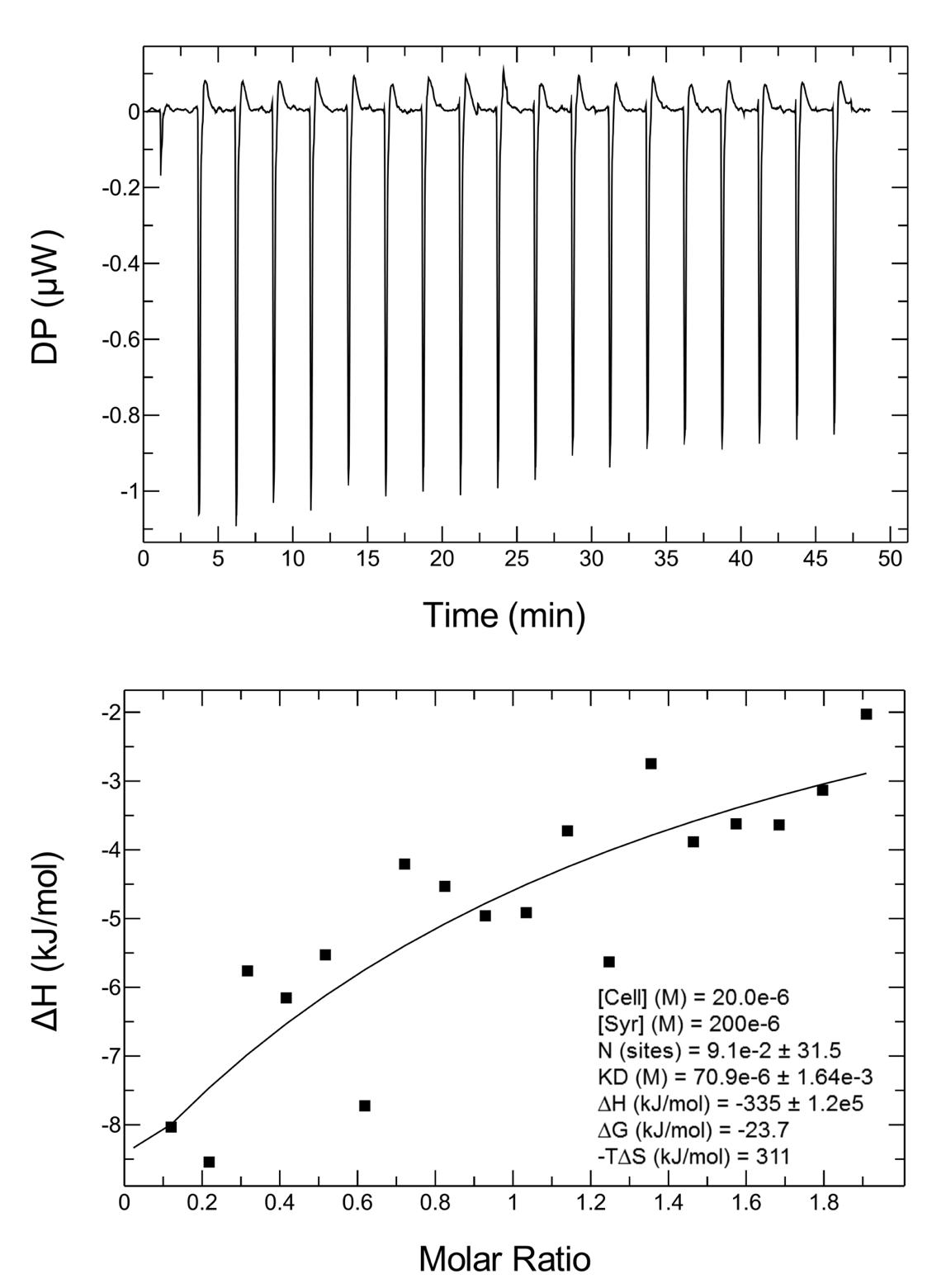
**

**Figure S45.** Specificity analysis of AI-CS-R3CC-T against BE from ITC experiment.

**Specificity: AI-CS-R3CC-T (Binding against DHEA)**

**
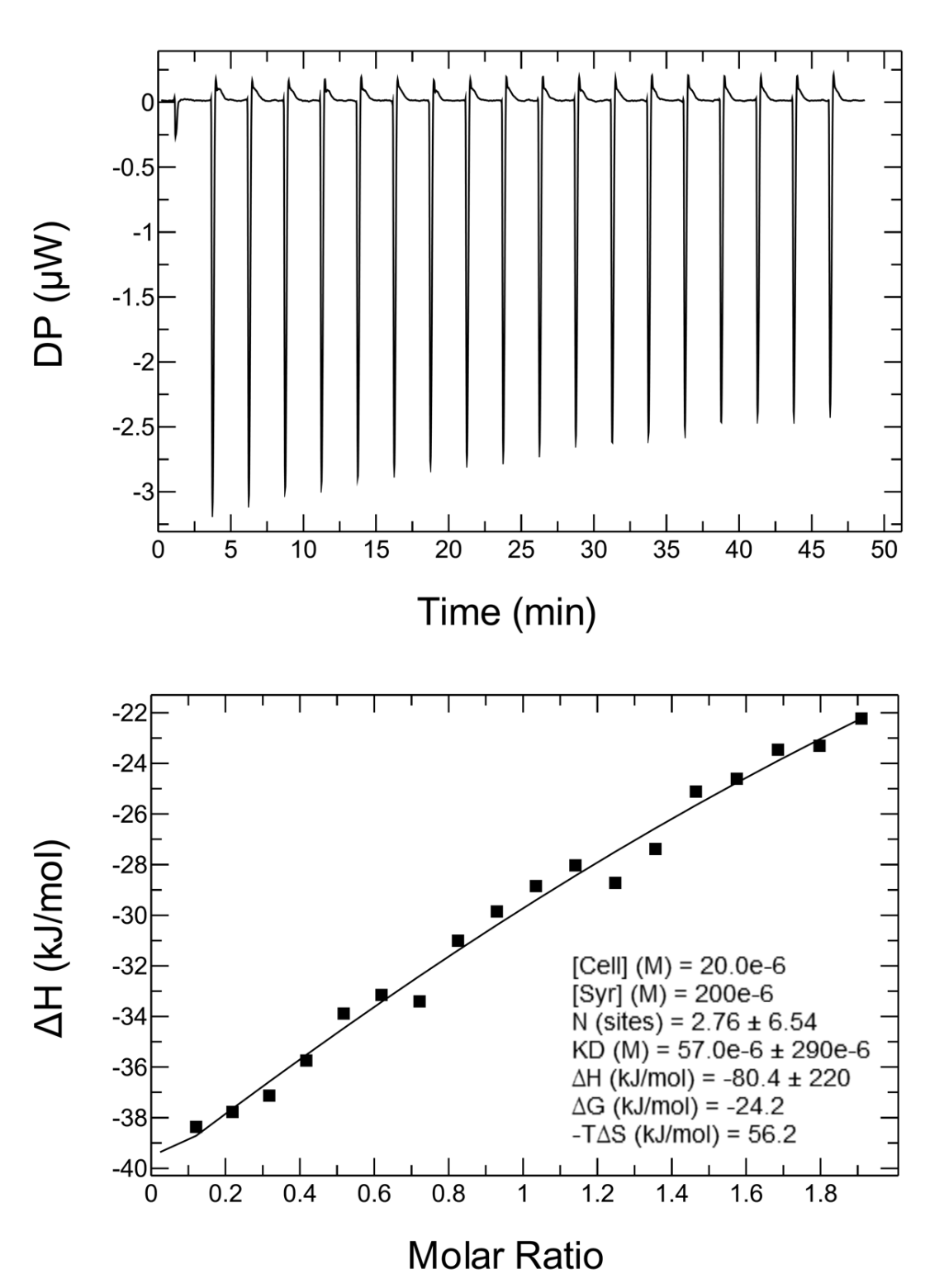
**

**Figure S46.** Specificity analysis of AI-CS-R3CC-T against DHEA from ITC experiment.

**Specificity: AI-CS-R3CC-T (Binding against CHO)**

**
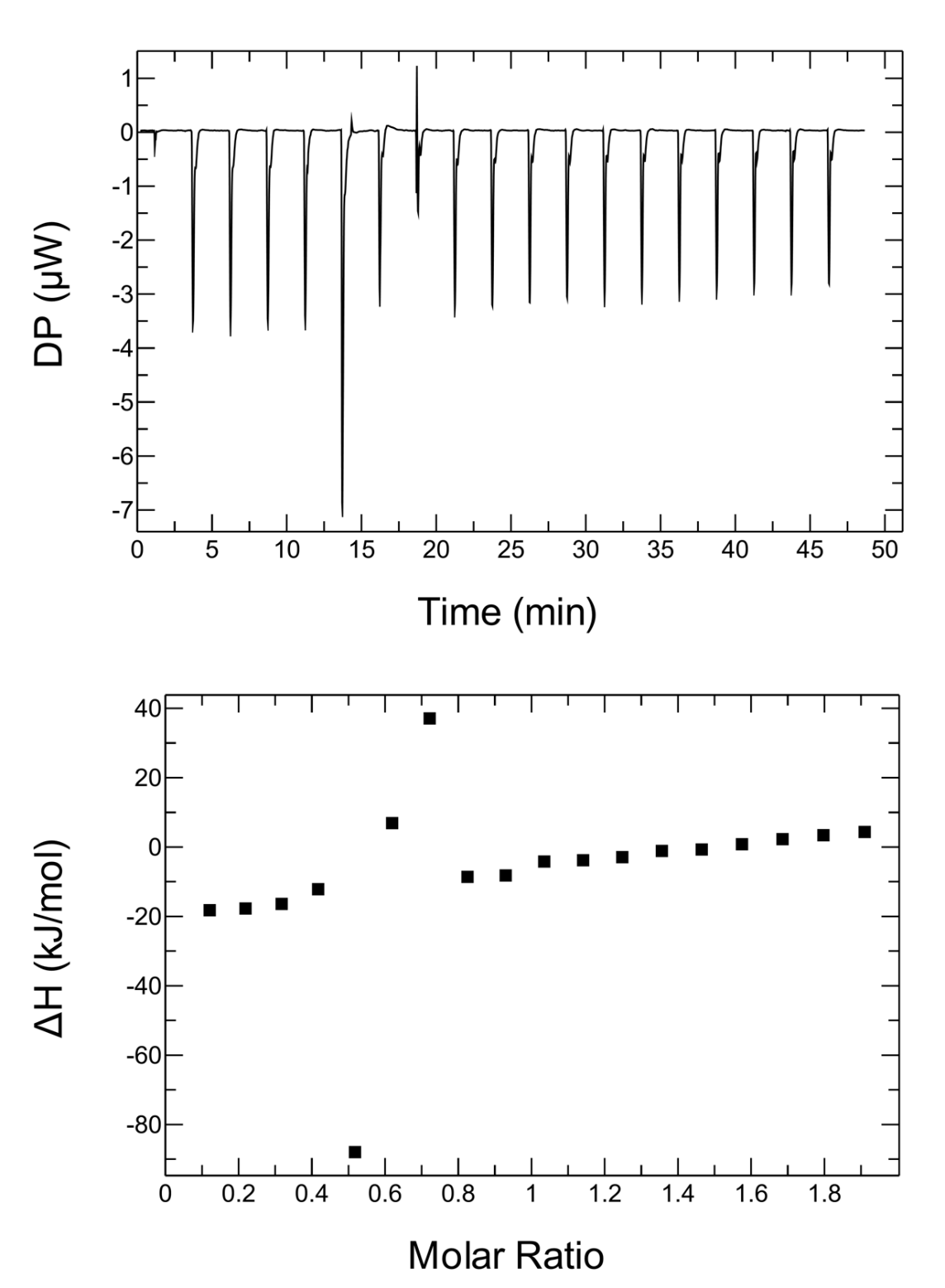
**

**Figure S47.** Specificity analysis of AI-CS-R3CC-T against CHO from ITC experiment.

**Specificity: AI-CS-R3CC-T (Binding against PRO)**

**
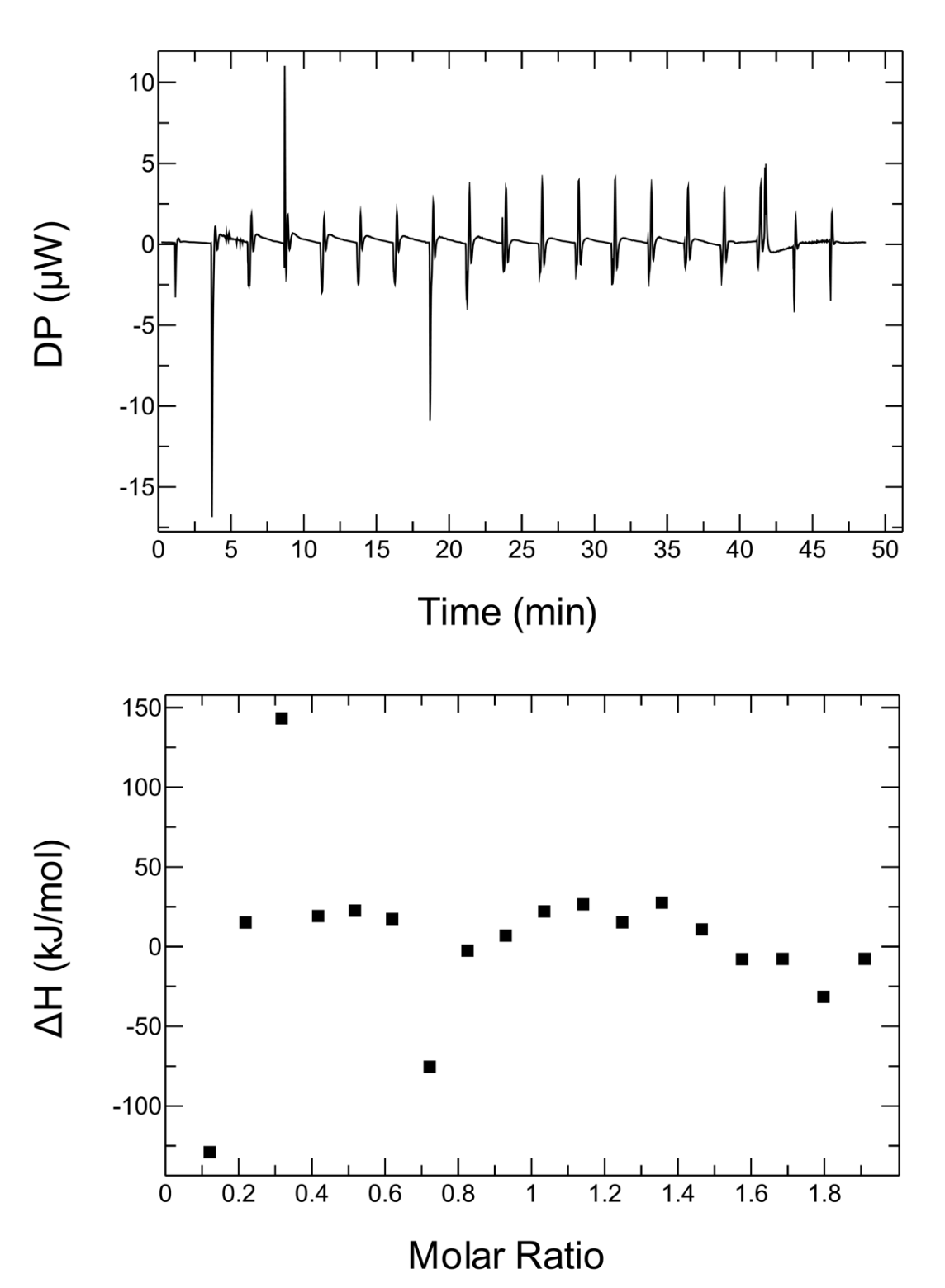
**

**Figure S48.** Specificity analysis of AI-CS-R3CC-T against PRO from ITC experiment.

**CS-Reported (Binding against CS)**

**
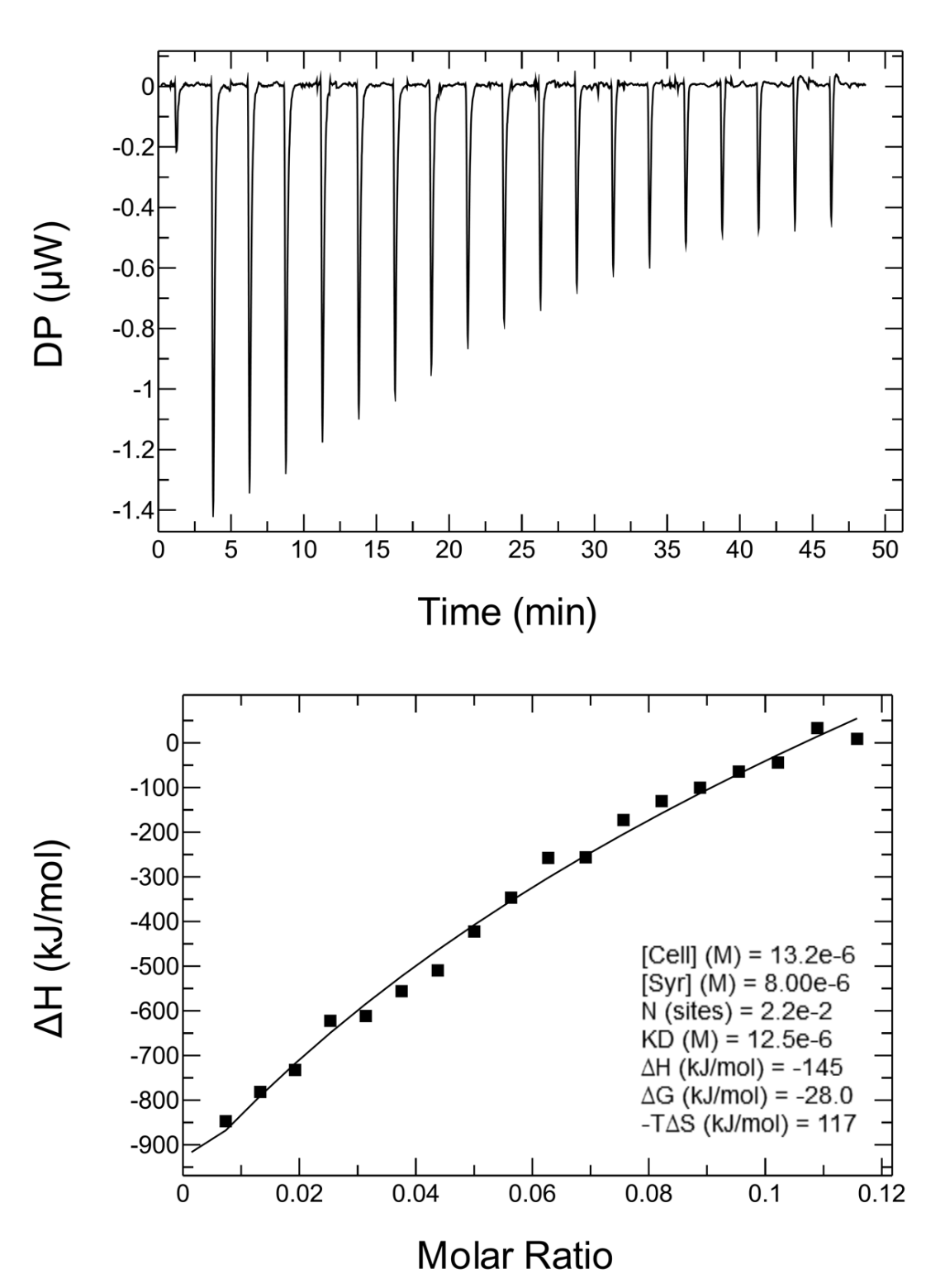
**

**Figure S49.** The affinity of CS-Reported (4) against CS from ITC experiment.

**NGS-2 (Manual Design Library Aptamer) (Binding against CS)**

**
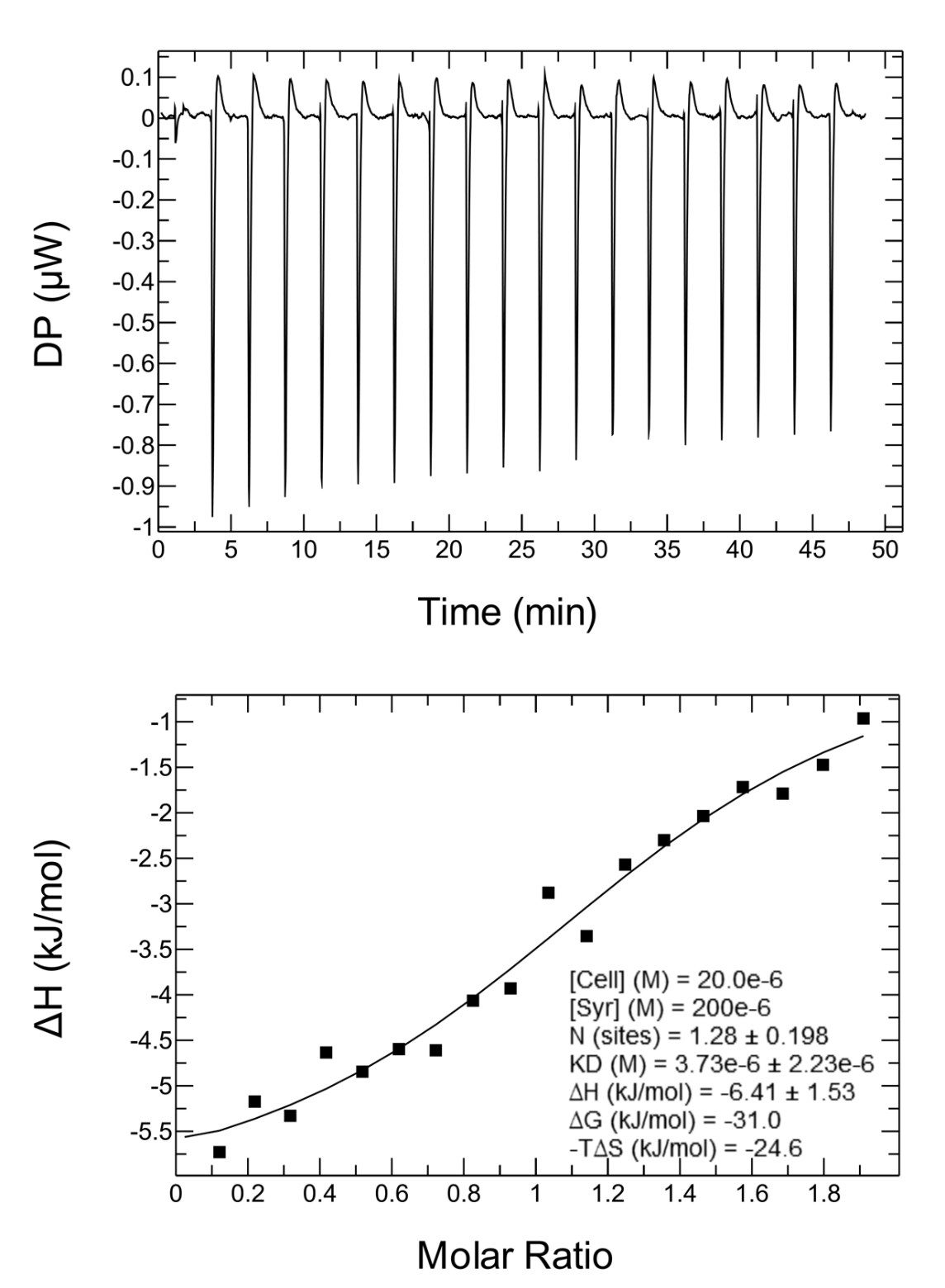
**

**Figure S50.** The affinity of NGS-2 against CS from ITC experiment.

**TES-R7MP (Binding against TES)**

**
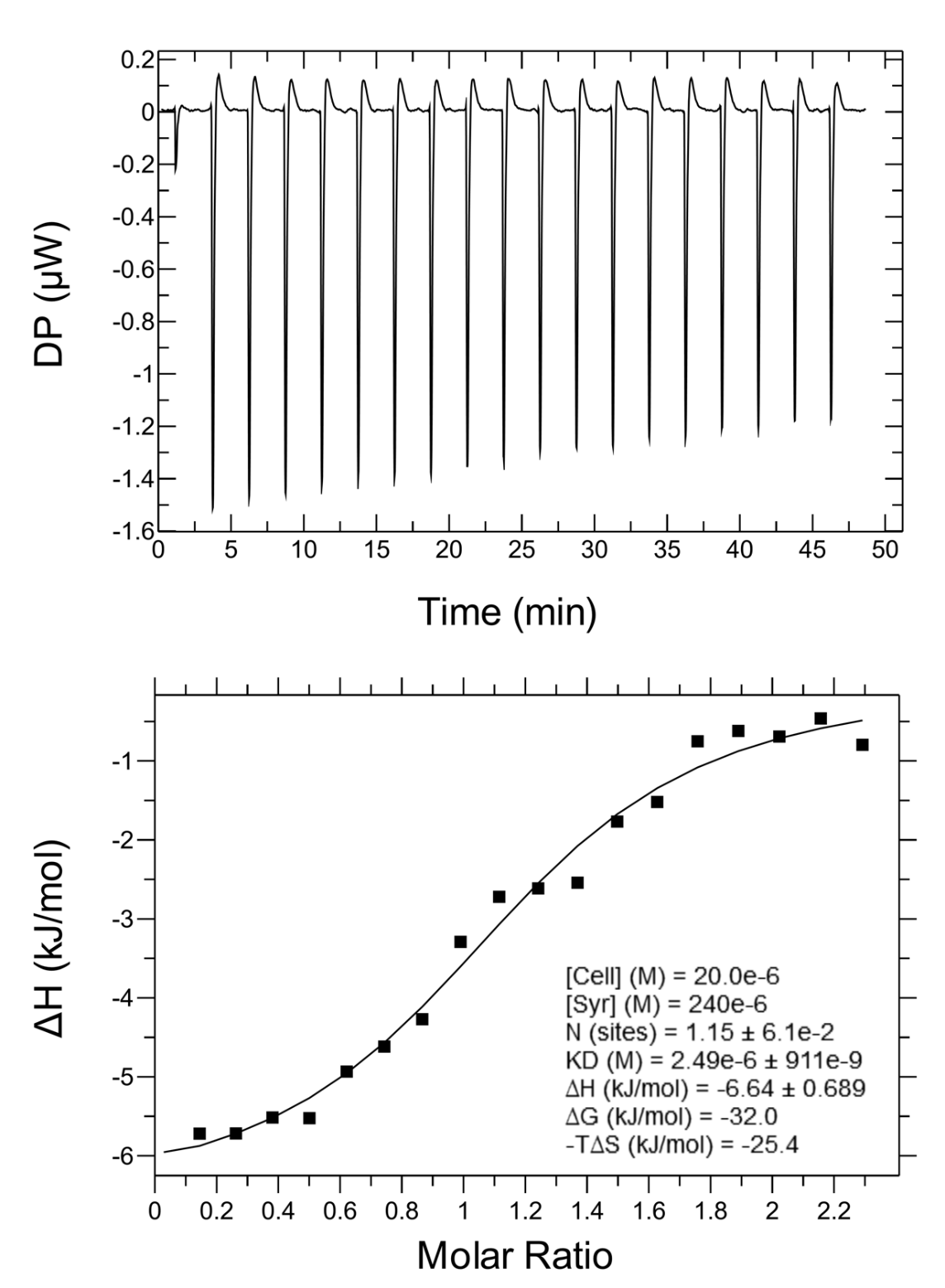
**

**Figure S51.** The affinity of TES-R7MP against TES from ITC experiment.

**TES-R7MP-T (Binding against TES)**

**
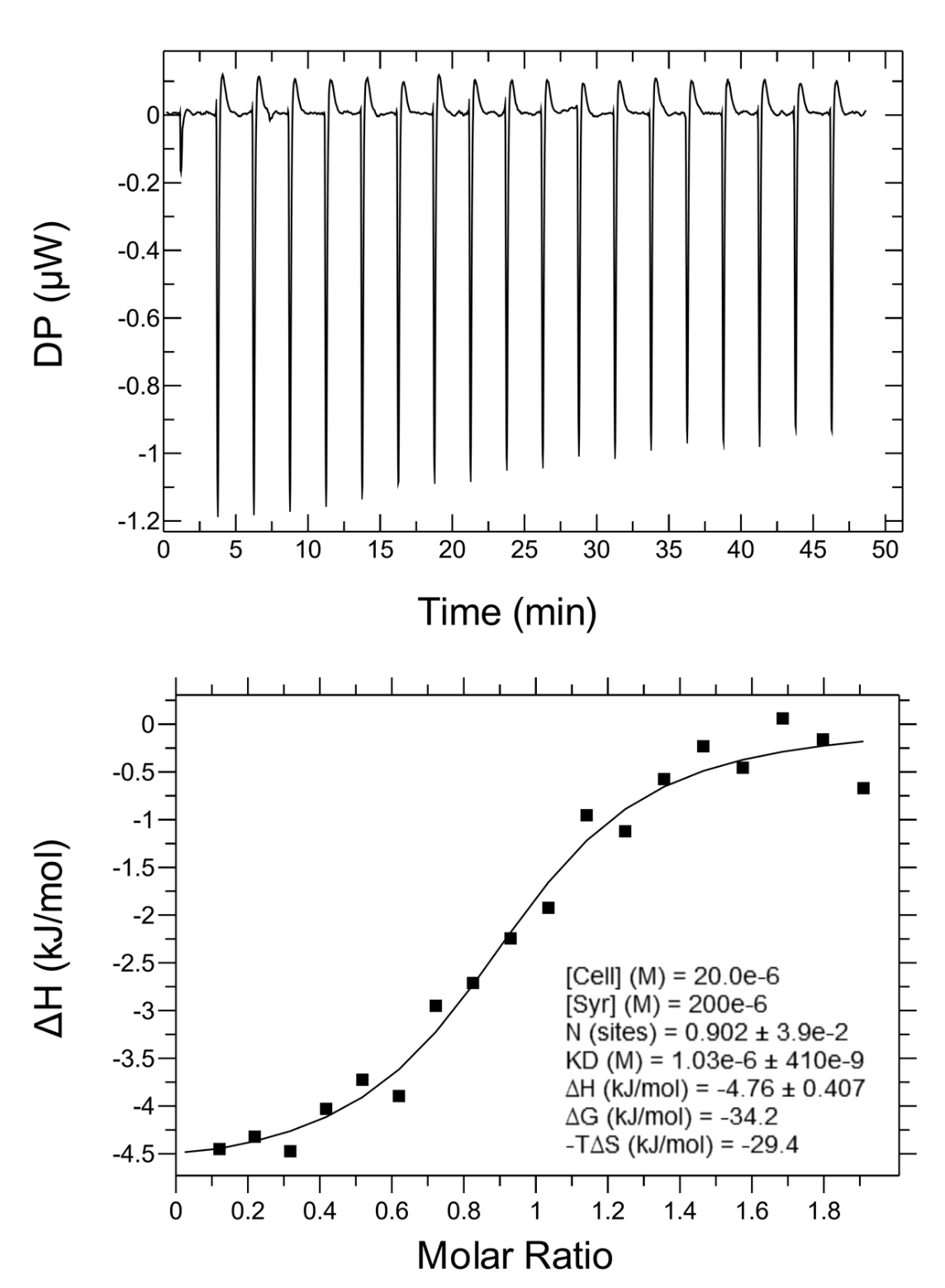
**

**Figure S52.** The affinity of TES-R7MP-T against TES from ITC experiment.

**Specificity: TES-R7MP-T (Binding against CS)**

**
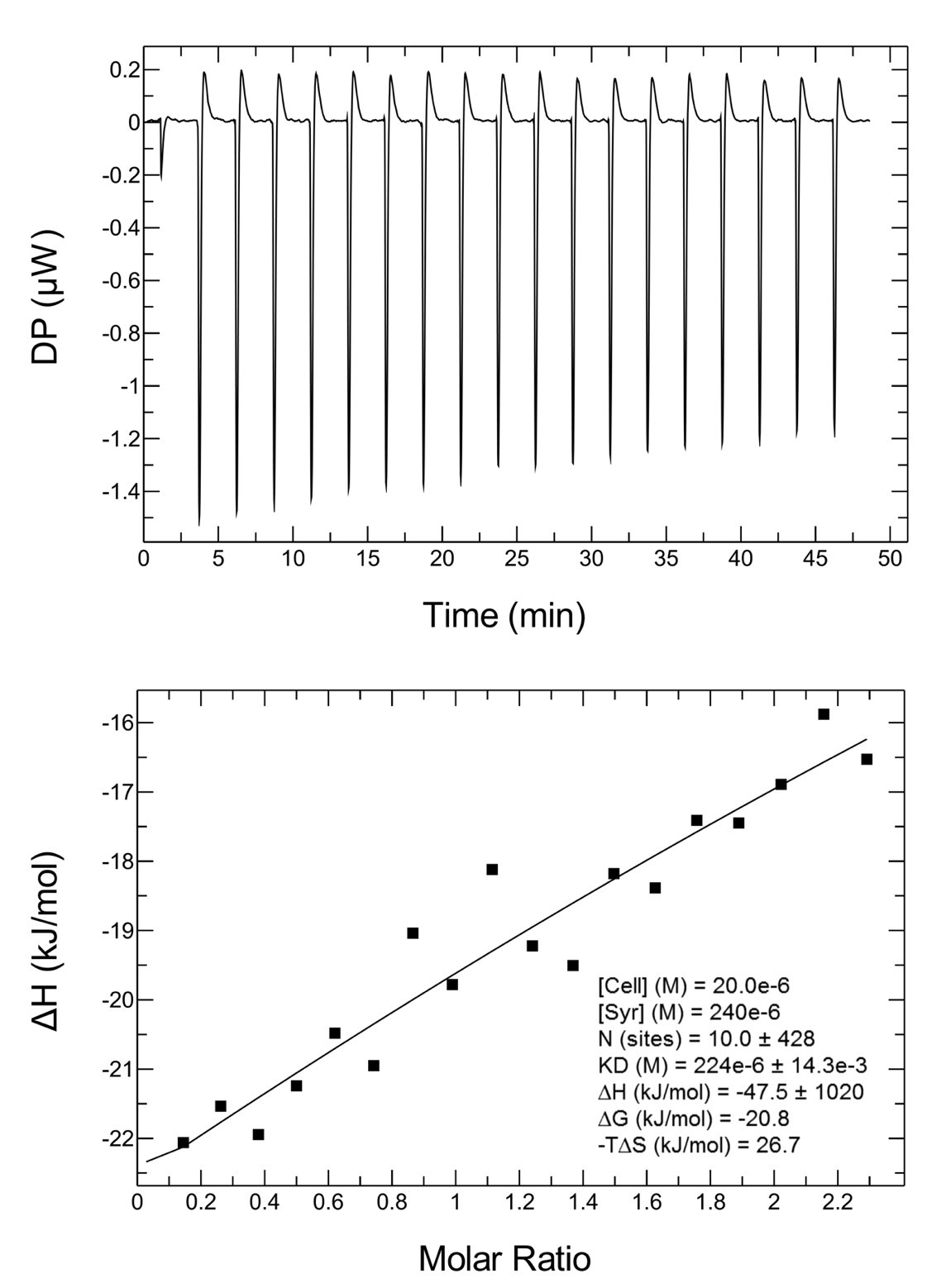
**

**Figure S53.** Specificity analysis of TES-R7MP-T against CS from ITC experiment.

**Specificity: TES-R7MP-T (Binding against BE)**

**
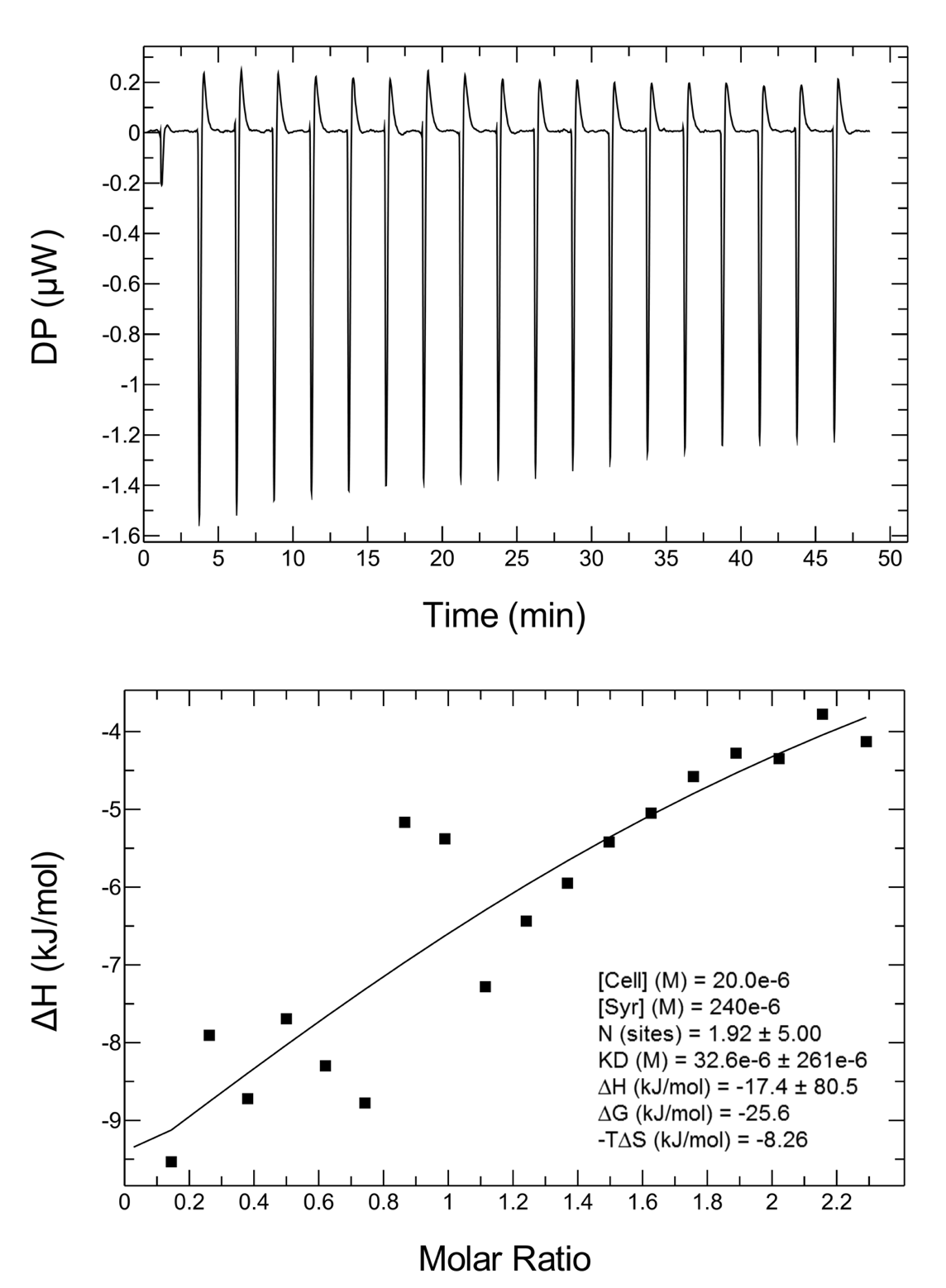
**

**Figure S54.** Specificity analysis of TES-R7MP-T against BE from ITC experiment.

**Specificity: TES-R7MP-T (Binding against DHEA)**

**
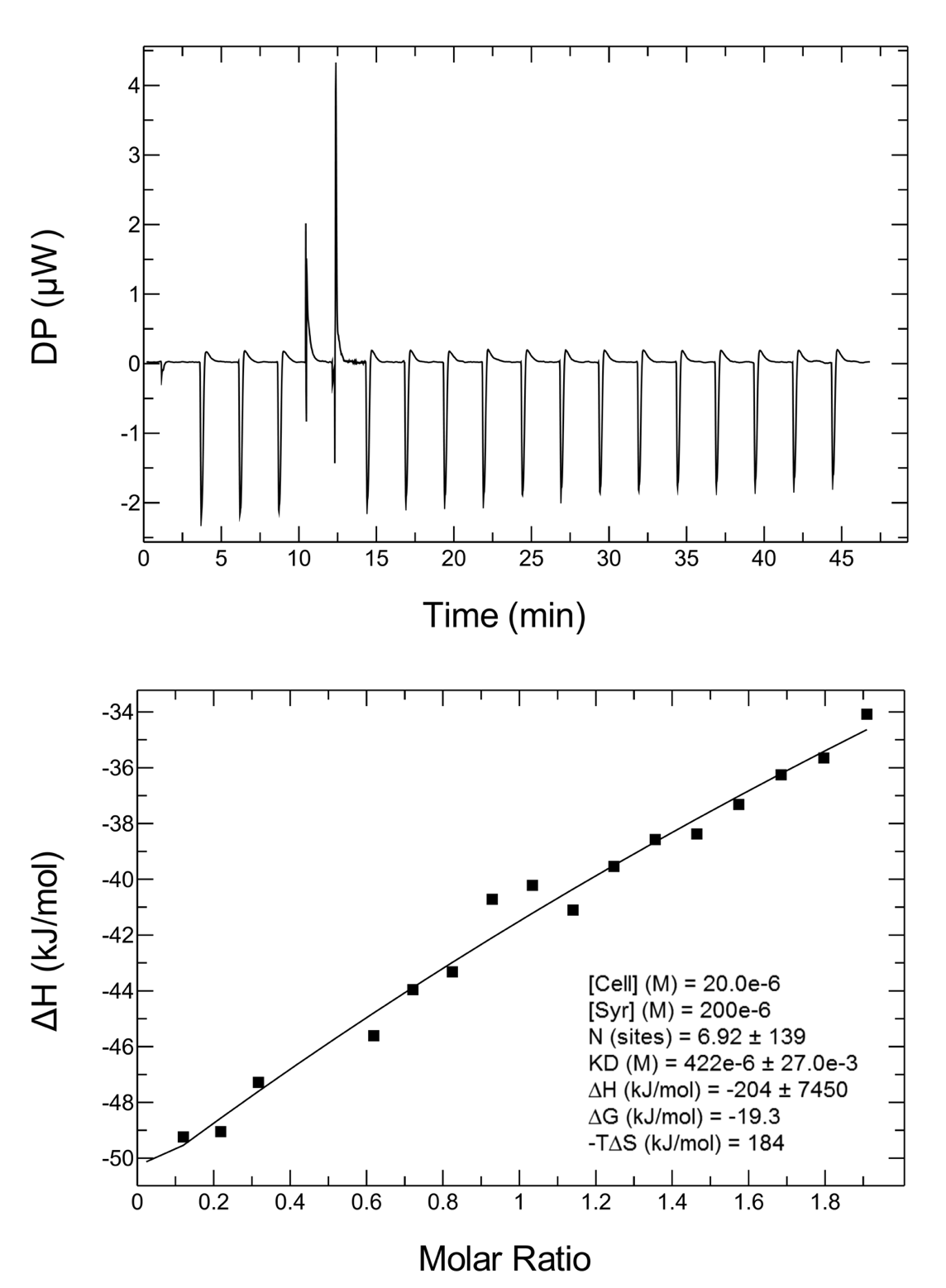
**

**Figure S55.** Specificity analysis of TES-R7MP-T against DHEA from ITC experiment.

**Specificity: TES-R7MP-T (Binding against CHO)**

**
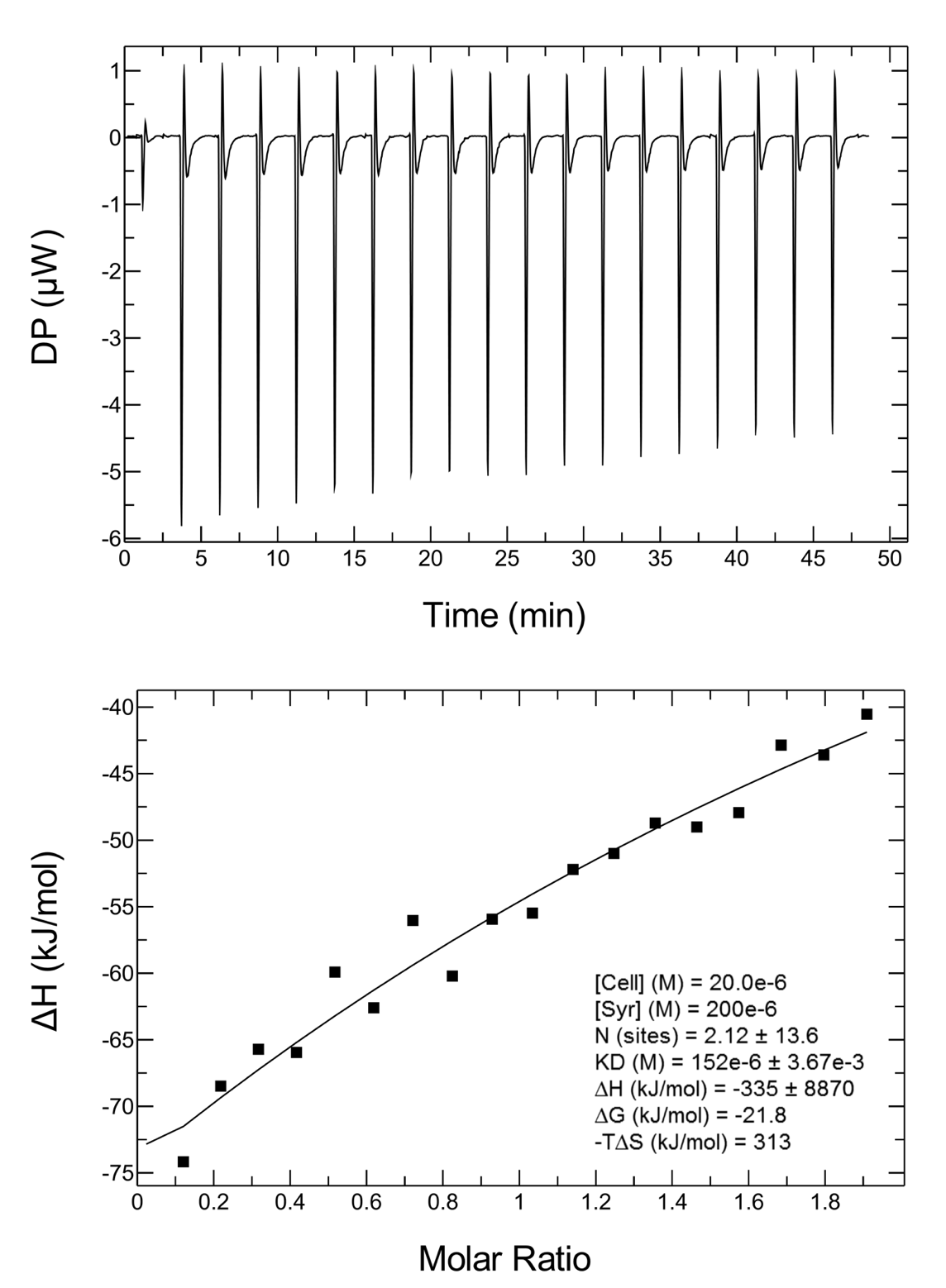
**

**Figure S56.** Specificity analysis of TES-R7MP-T against CHO from ITC experiment.

**Specificity: TES-R7MP-T (Binding against PRO)**

**
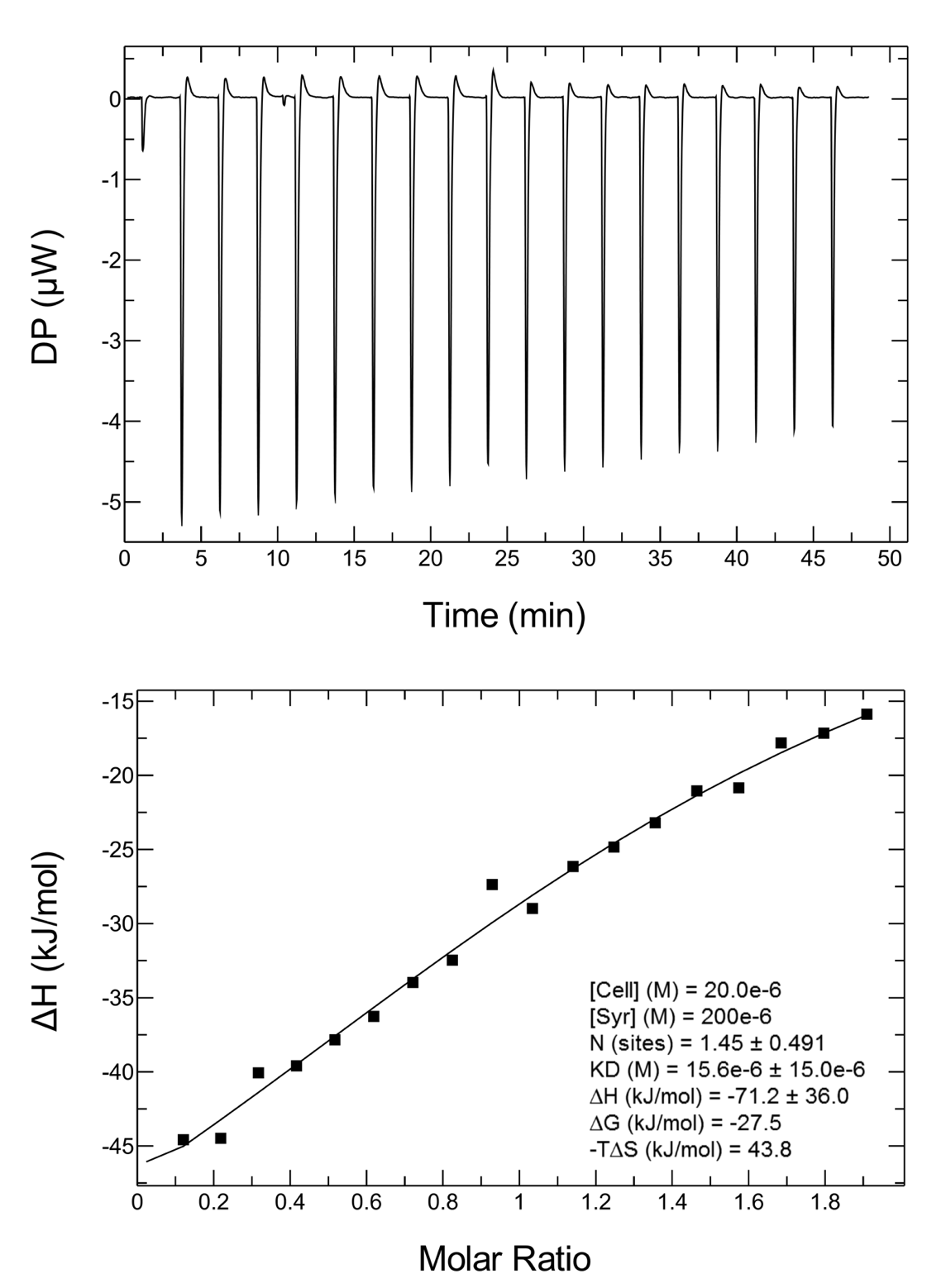
**

**Figure S57.** Specificity analysis of TES-R7MP-T against PRO from ITC experiment.

**AI-TES-R3R5HD (Binding against TES)**

**
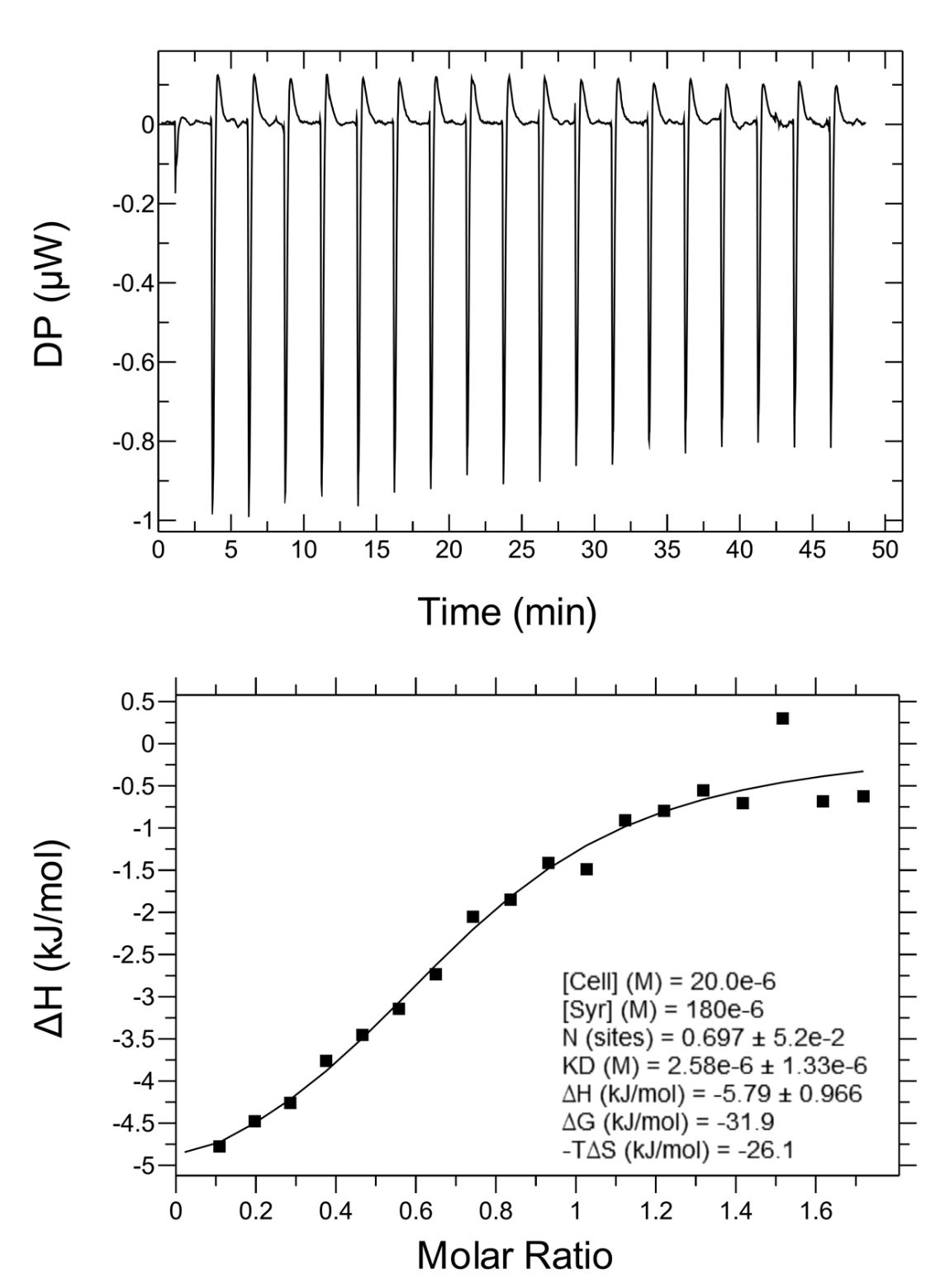
**

**Figure S58.** The affinity of AI-TES-R3R5HD against TES from ITC experiment.

**AI-TES-R3R5HD-T (Binding against TES)**

**
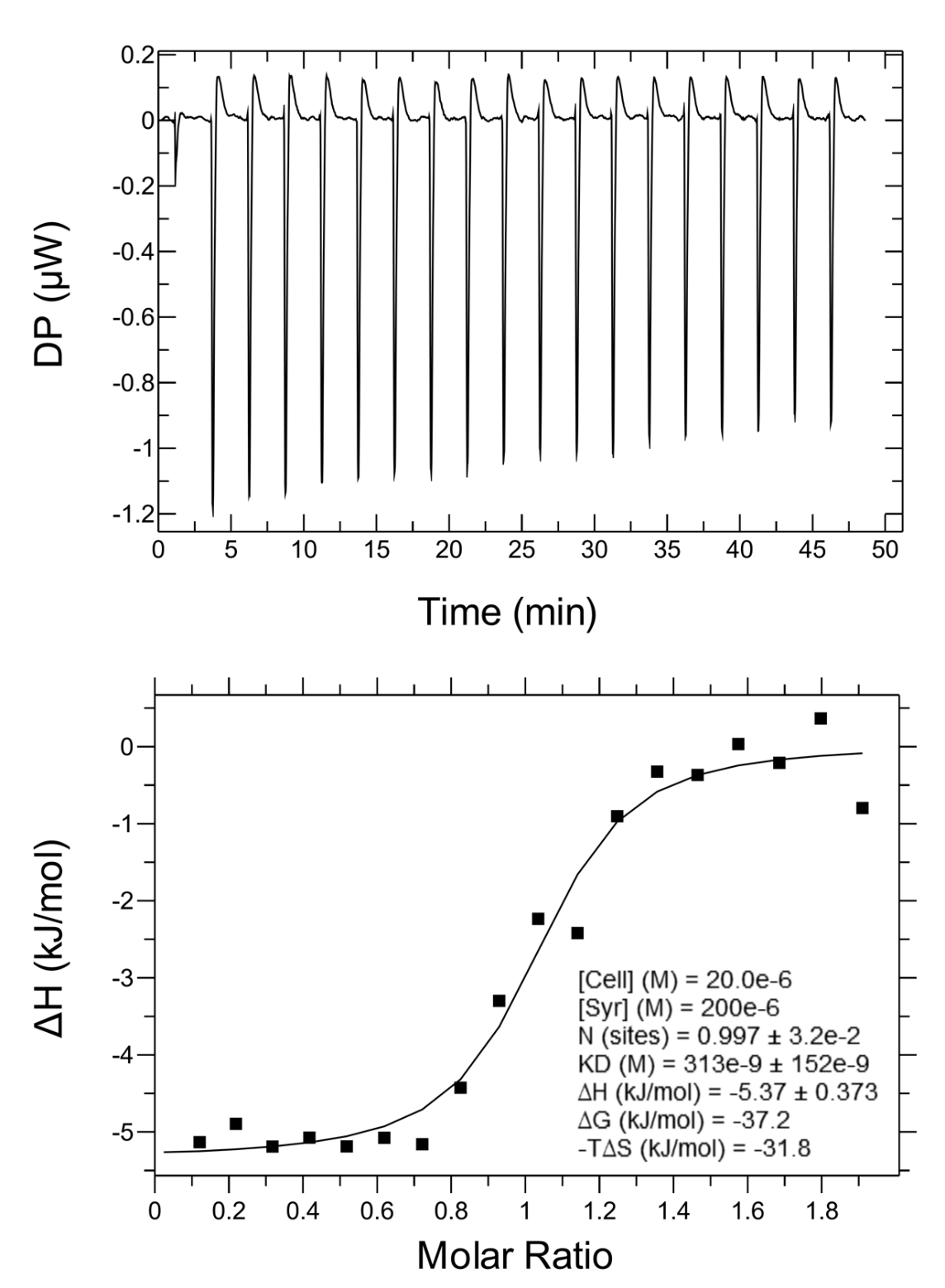
**

**Figure S59.** The affinity of AI-TES-R3R5HD-T against TES from ITC experiment.

**Specificity: AI-TES-R3R5HD-T (Binding against CS)**

**
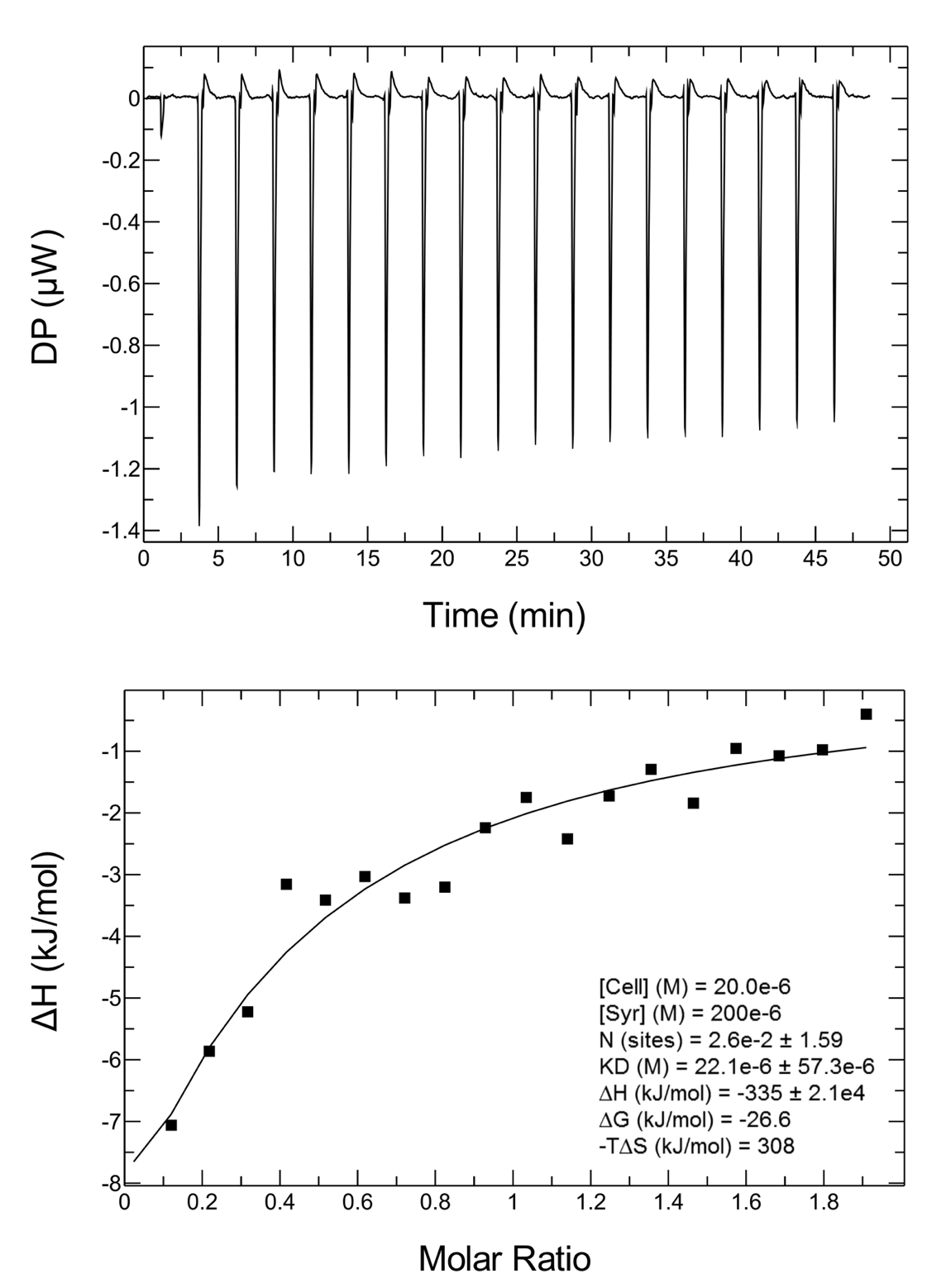
**

**Figure S60.** Specificity analysis of AI-TES-R3R5HD-T against CS from ITC experiment.

**Specificity: AI-TES-R3R5HD-T (Binding against BE)**

**
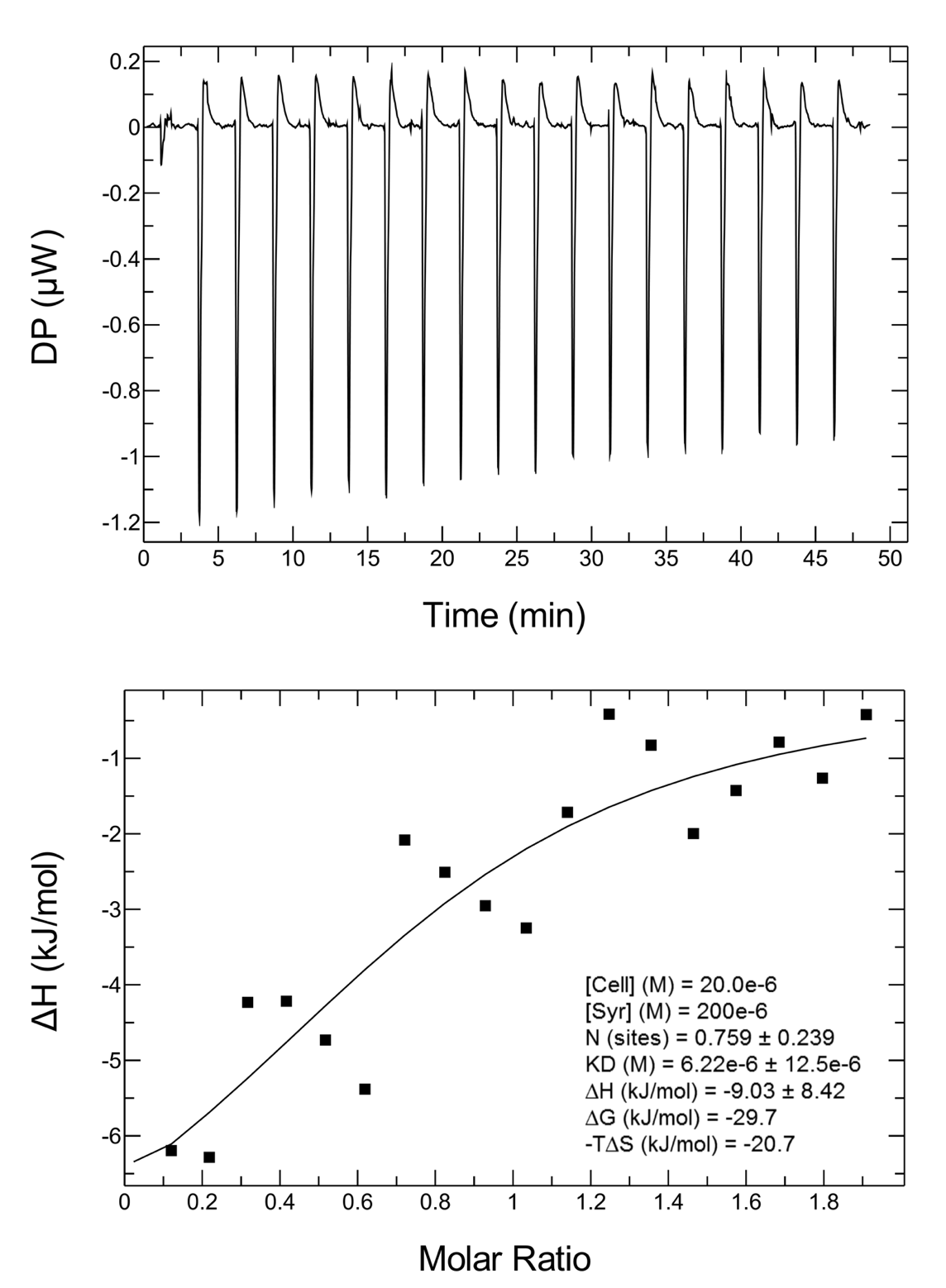
**

**Figure S61.** Specificity analysis of AI-TES-R3R5HD-T against BE from ITC experiment.

**Specificity: AI-TES-R3R5HD-T (Binding against DHEA)**

**
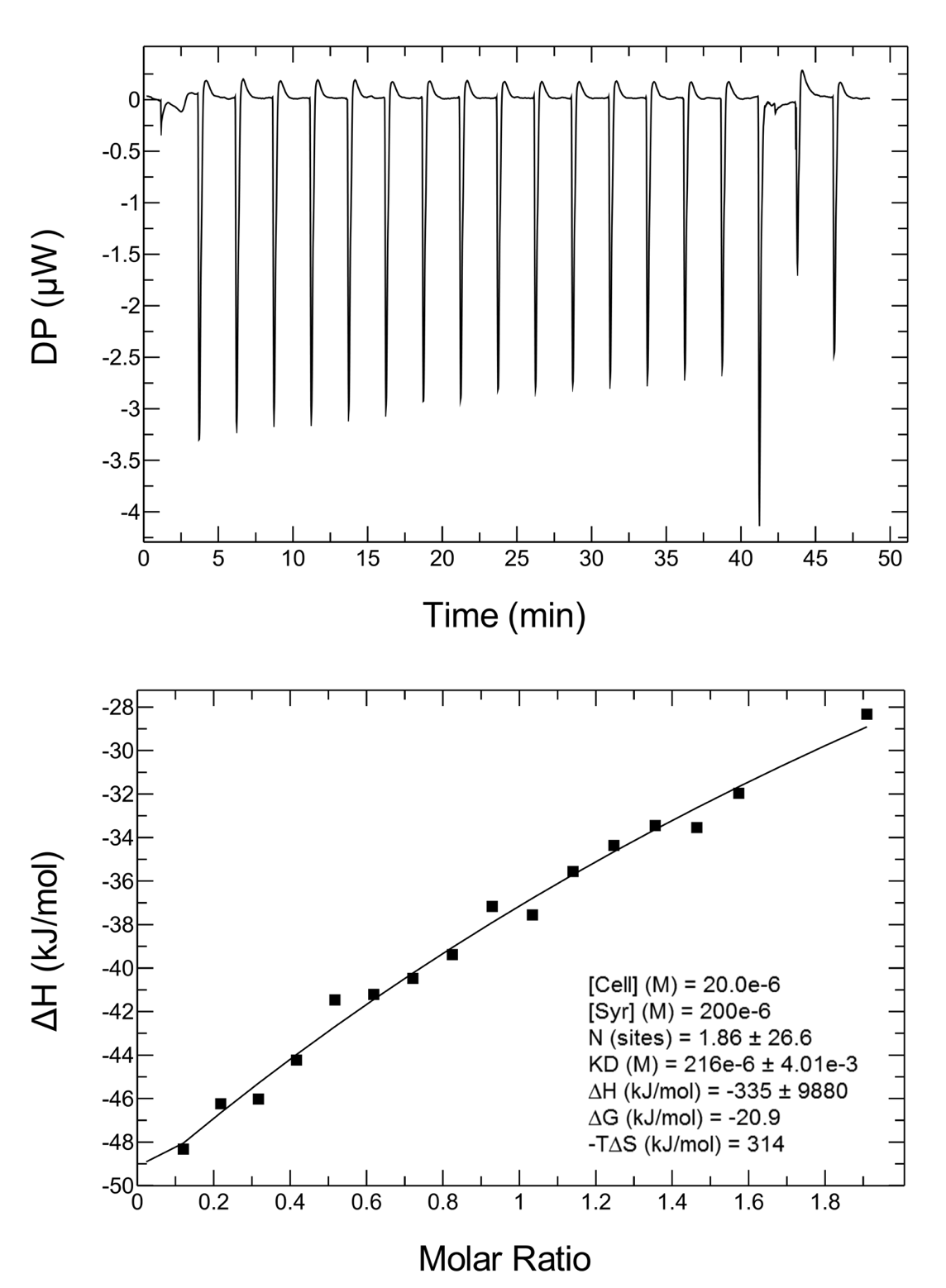
**

**Figure S62.** Specificity analysis of AI-TES-R3R5HD-T against DHEA from ITC experiment.

**Specificity: AI-TES-R3R5HD-T (Binding against CHO)**

**
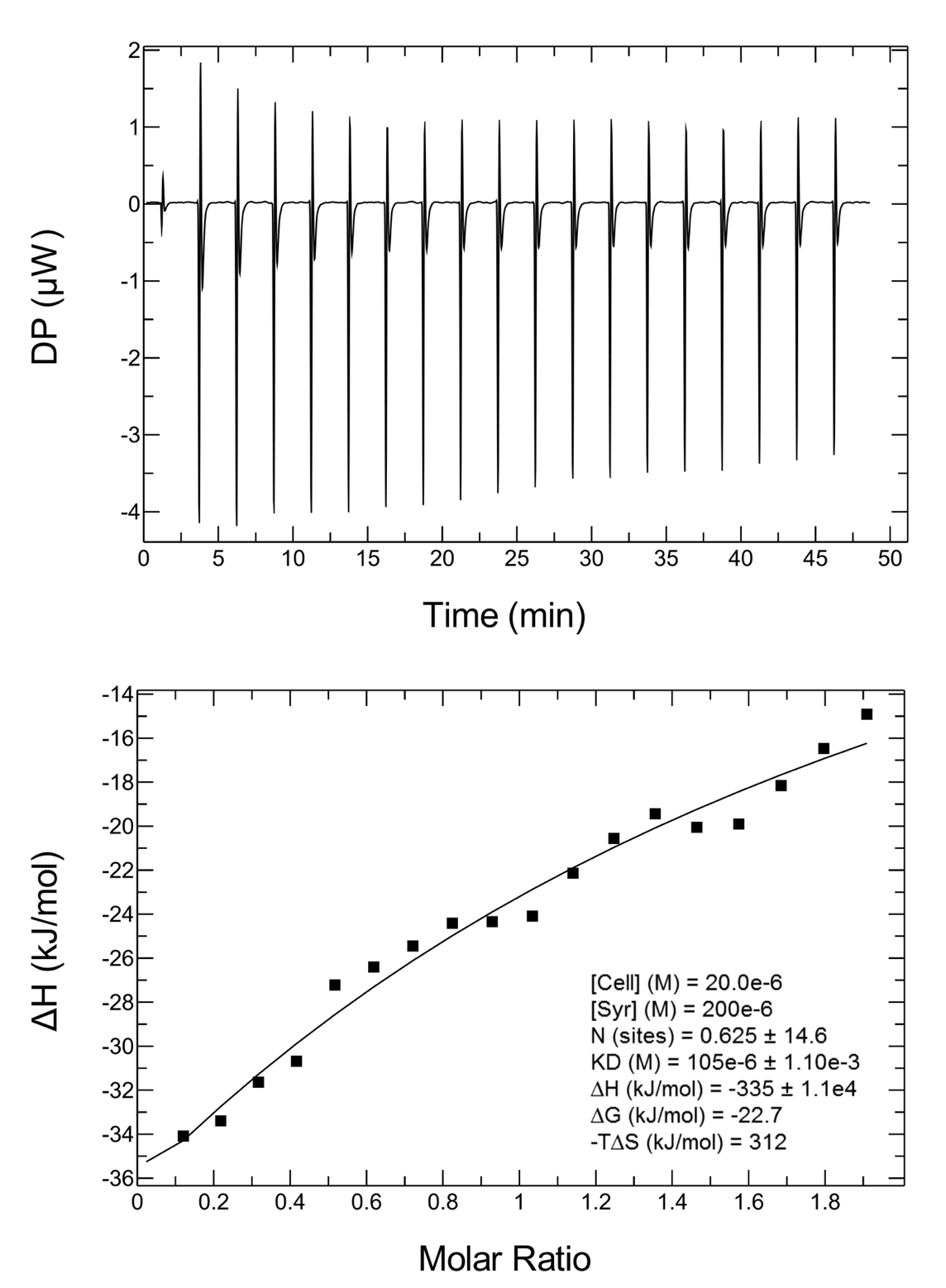
**

**Figure S63.** Specificity analysis of AI-TES-R3R5HD-T against CHO from ITC experiment.

**Specificity: AI-TES-R3R5HD-T (Binding against PRO)**

**
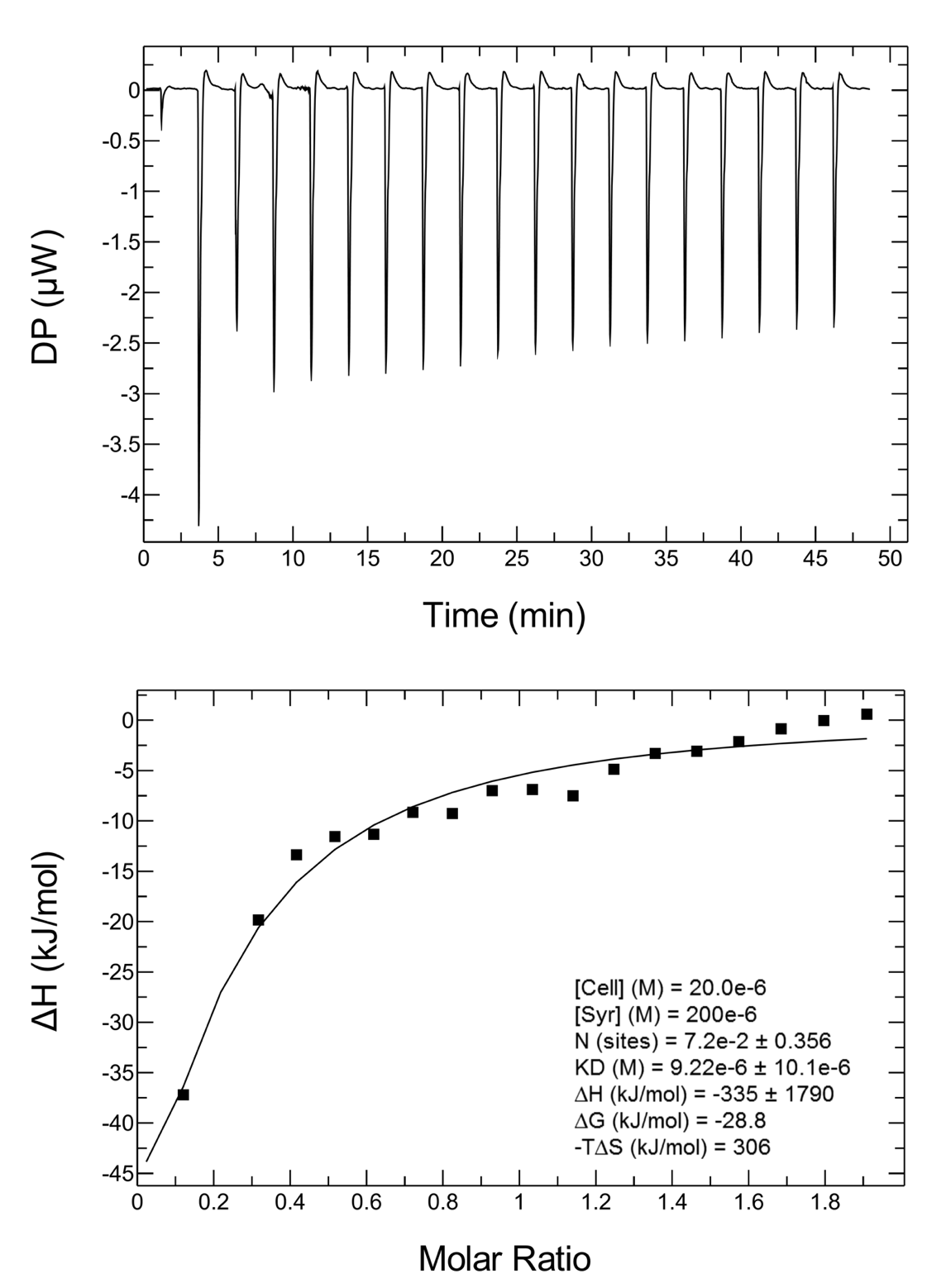
**

**Figure S64.** Specificity analysis of AI-TES-R3R5HD-T against PRO from ITC experiment.

**TES-Reported (Binding against TES)**

**
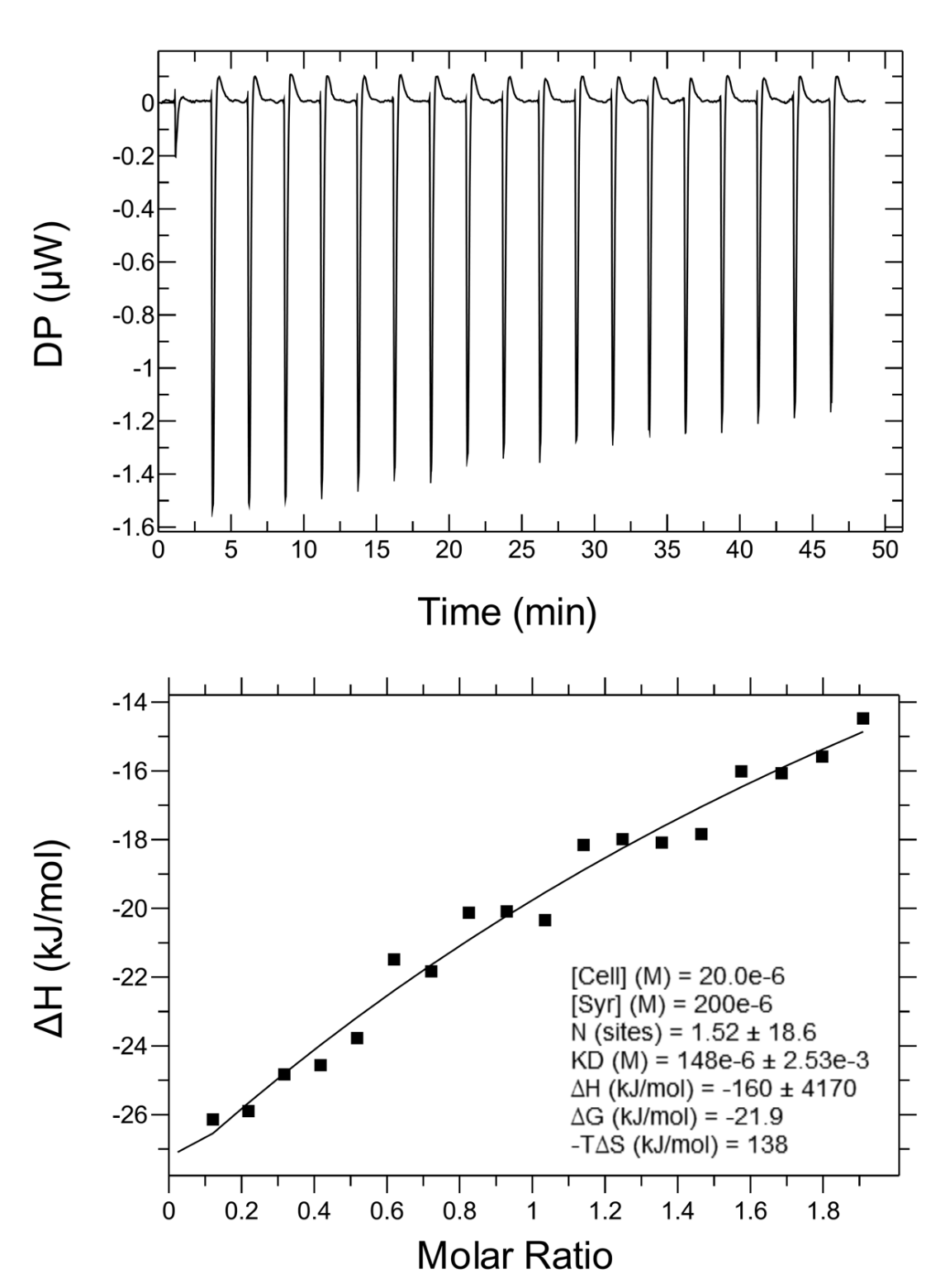
**

**Figure S65.** The affinity of TES-Reported (7) against TES from ITC experiment.

## Figure S66 | Collected Steroid Aptamer Classes Distribution

**
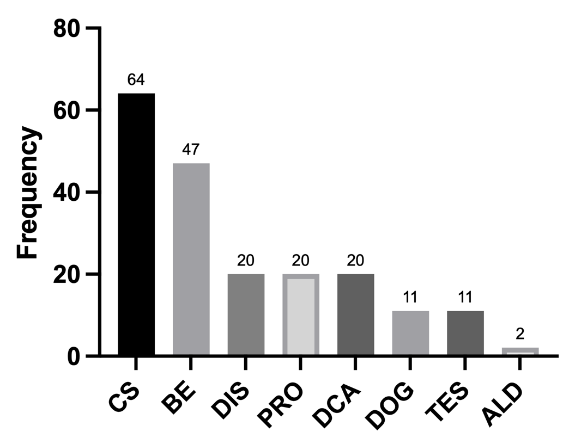
**

**Figure S66.** Distribution of 195 collected steroid aptamers from various sources.

The hydrocortisone (CS) class has the most aptamers of 64, and the TES class has 11 aptamer sequences, which are two case studies in this work. These two extreme cases were selected to test out the imbalanced handling of the model. We have not added any data augmentation to generate more aptamer sequences, and this is because each aptamer was provided from various sources according to the experimental selection with high quality. A nonsensical data augmentation will only decrease the quality of our raw dataset and create more non-useful aptamers.

## Figure S67-S70 | Deep Learning Models Performance Comparison


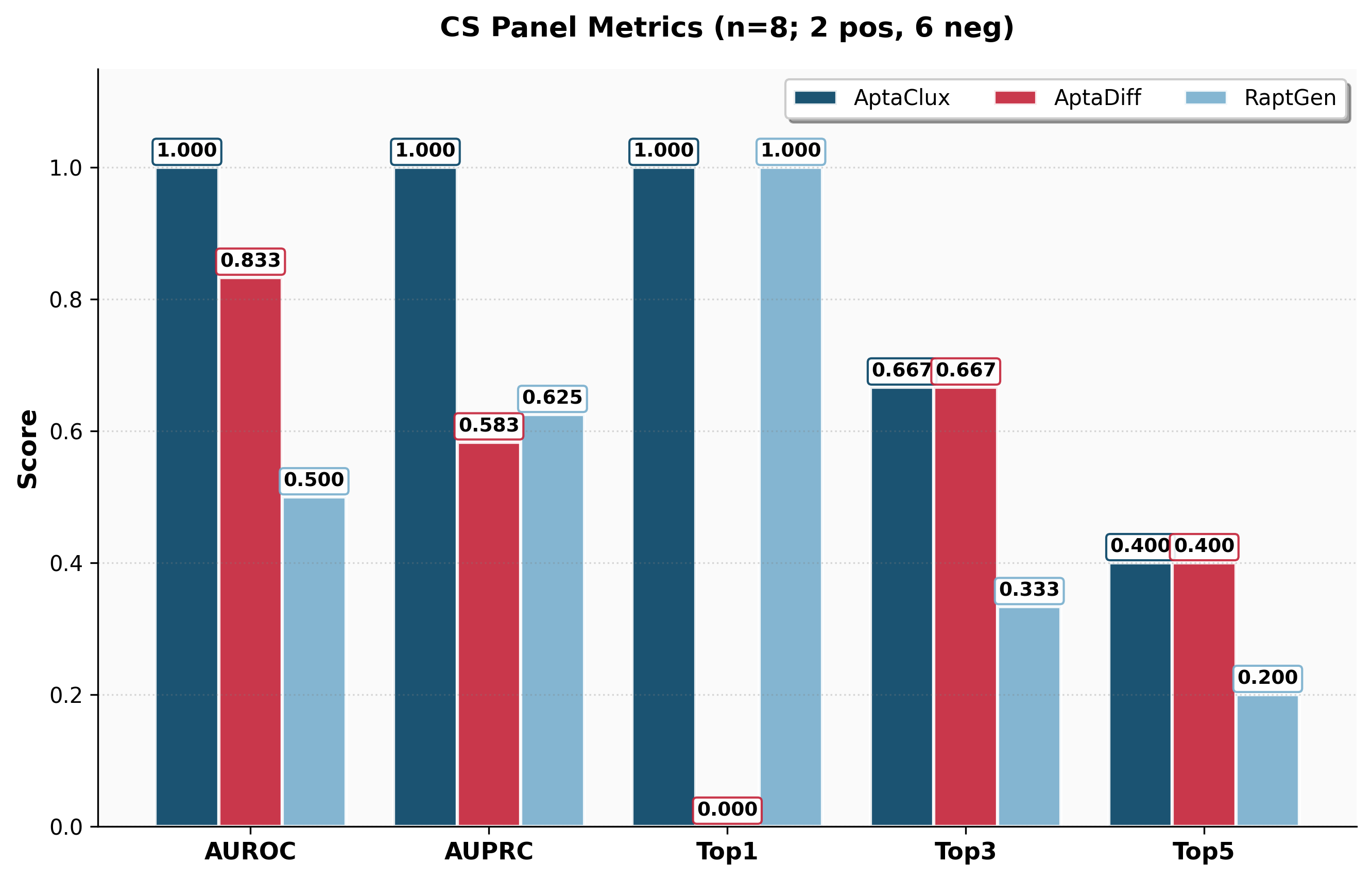


**Figure S67.** CS panel ranking performance (n=8; 2 binders, 6 non-binders).

Bars show AUROC, AUPRC and Top-k hit-rate (k=1/3/5) for AptaClux, AptaDiff and RaptGen; values are annotated on the bars. All models were trained only on CS-R3 with identical 39-nt preprocessing (no adapters) and evaluated on the same labeled panel. For each method, a per-sequence score was defined as −log density of the encoder latent mean (μ) under a Gaussian KDE fitted on that model’s CS-R3 μ’s (bandwidth by 5-fold CV on R3 only); panel labels were used only for evaluation. Key explanation of the criterion selection can be found in Notes S8. Under this protocol, AptaClux achieves AUROC=AUPRC=1.00 and Top-1=1.00; AptaDiff: AUROC=0.83, AUPRC=0.58; RaptGen: AUROC=0.50, AUPRC=0.63.


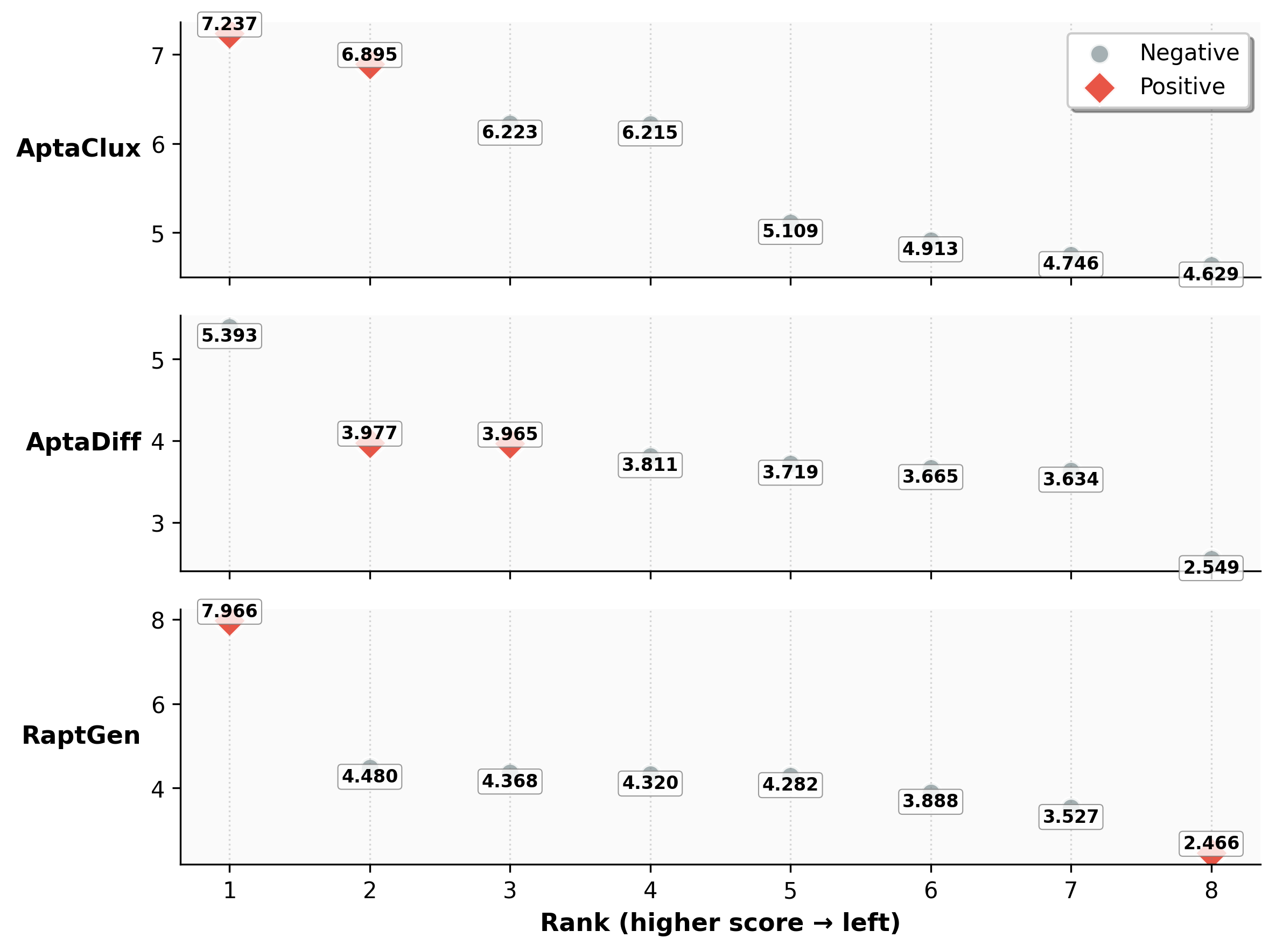


**Figure S68.** Per-method ranking of the CS panel (n=8; 2 binders, 6 non-binders).

Sequences are ordered by score (x-axis; higher → left). Markers show **non-binders** (circles) and **binders** (diamonds); labels are raw scores. The score is identical to Fig. S67. Under this protocol, **AptaClux** ranks both binders **1st–2nd, AptaDiff** ranks them **2nd–3rd,** and **RaptGen** ranks one binder **1st** and the other **8th**.


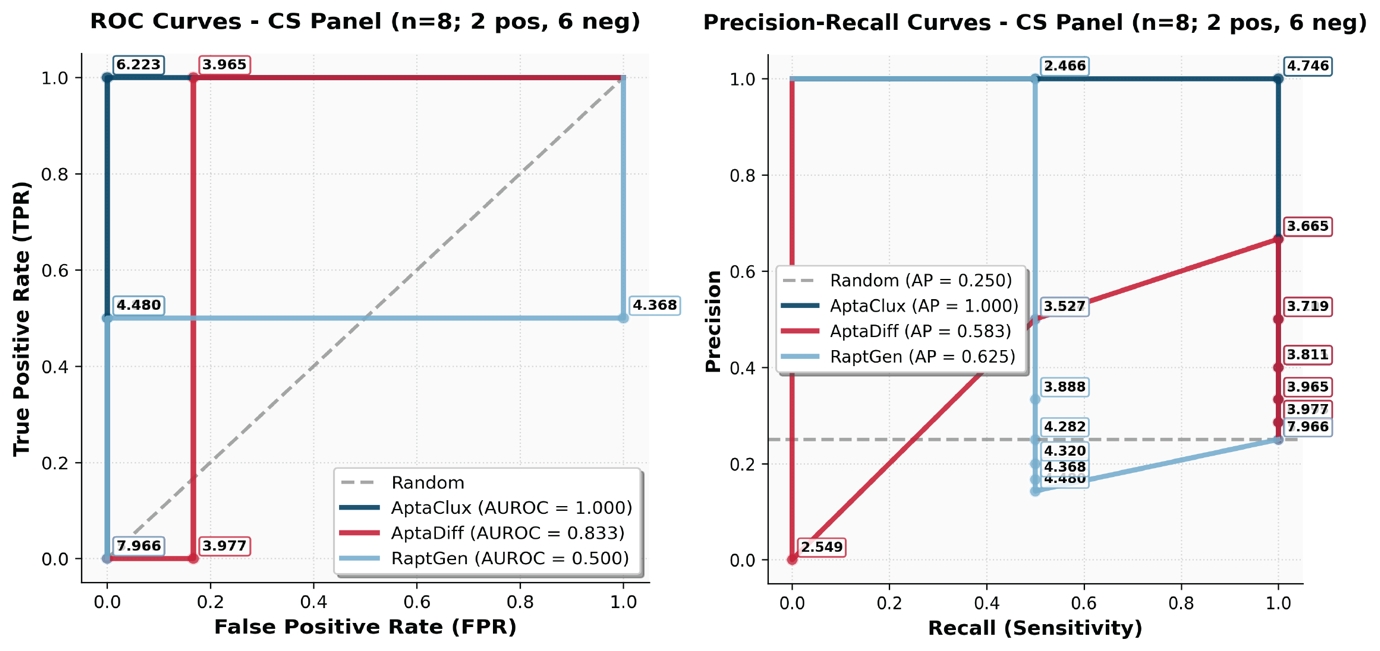


**Figure S69-S70. ROC (left) and precision–recall (PR, right) curves on the CS panel (n=8; 2 binders, 6 non-binders).**

All models were trained on **CS-R3** and scored sequences as **−log density of the encoder latent mean (μ) under a Gaussian KDE fitted on CS-R3 μ’s** (bandwidth by 5-fold CV on R3 only). Due to the small sample size, curves are step-wise; numbers mark score thresholds at breakpoints. Area metrics: **AptaClux** AUROC = 1.00, AP = 1.00; **AptaDiff** AUROC = 0.833, AP = 0.583; **RaptGen** AUROC = 0.500, AP = 0.625. The grey dashed line indicates the random baseline (AUROC = 0.5; AP = 0.25).

## Figure S71-S72 | Experimental Verification of Molecular Dynamics and Dockings Predicted Contacts via ITC


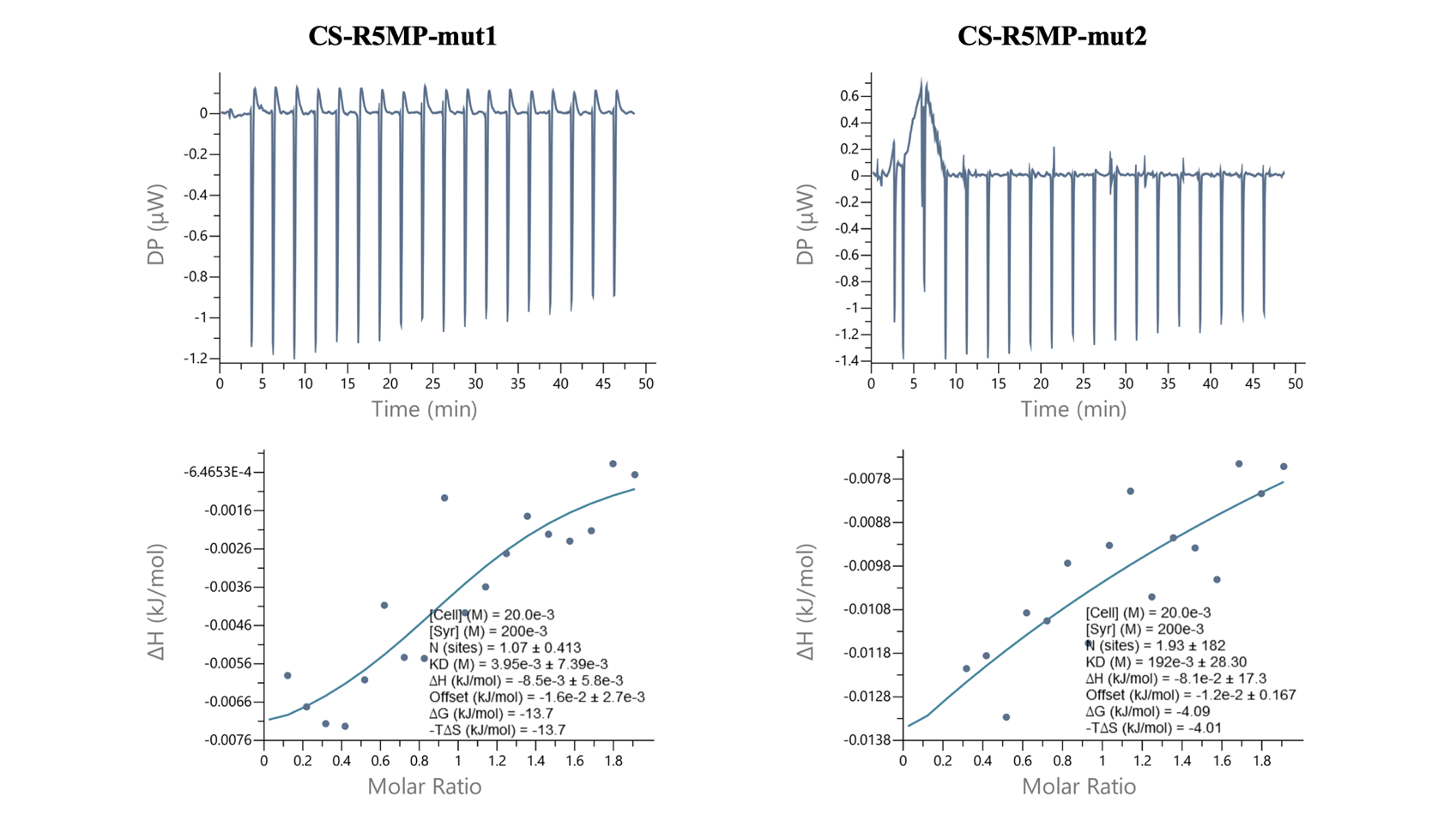


**Figure S71.** Point-mutation ITC validates MD/docking contacts.
Raw thermograms (top; DP vs time) and integrated heats with one-site fits (bottom; ΔH vs molar ratio) for CS-R5MP-mut1 (left) and CS-R5MP-mut2 (right), in the same buffer and injection scheme as the wild-type. Heats of dilution were measured and subtracted; fits and 95% CIs were obtained with MicroCal PEAQ-ITC Analysis. Both mutations weaken binding relative to wild-type CS-R5MP (see Fig. S28), with mut2 showing strongly diminished/near-abolished binding (flattened thermogram and poor saturation of the isotherm), consistent with disruption of the MD/docking-predicted contacts.


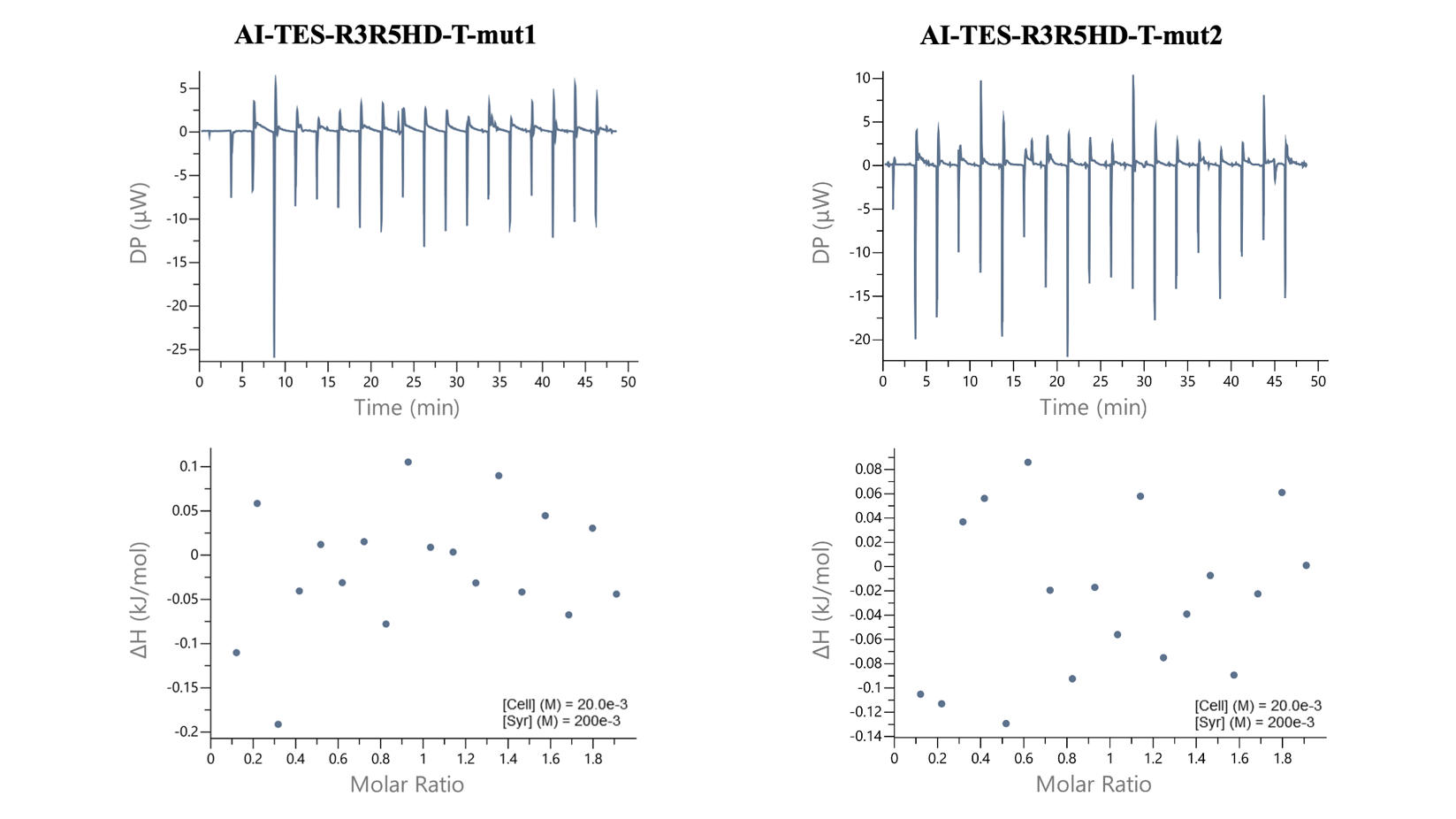


**Figure S72.** Mutational disruption abolishes binding in TES aptamer controls.
Raw thermograms (top; DP vs time) and integrated heats (bottom; ΔH per injection) for AI-TES-R3R5HD-T-mut1 (left) and AI-TES-R3R5HD-T-mut2 (right). Experiments used the same buffer, injection scheme as wild-type. Heats of dilution were measured and subtracted as in Note S1. Unlike wild-type, both mutants show near-zero, unsaturating heats across the titration with no sigmoidal isotherm; one-site fitting is not reliable and no K_D_ is reported (n.b., no measurable binding). The loss of signal is consistent with the MD/docking-predicted contacts being disrupted by the mutations.

## Figure S73-S75 | AptaVAE Initial Library Statistical Validation


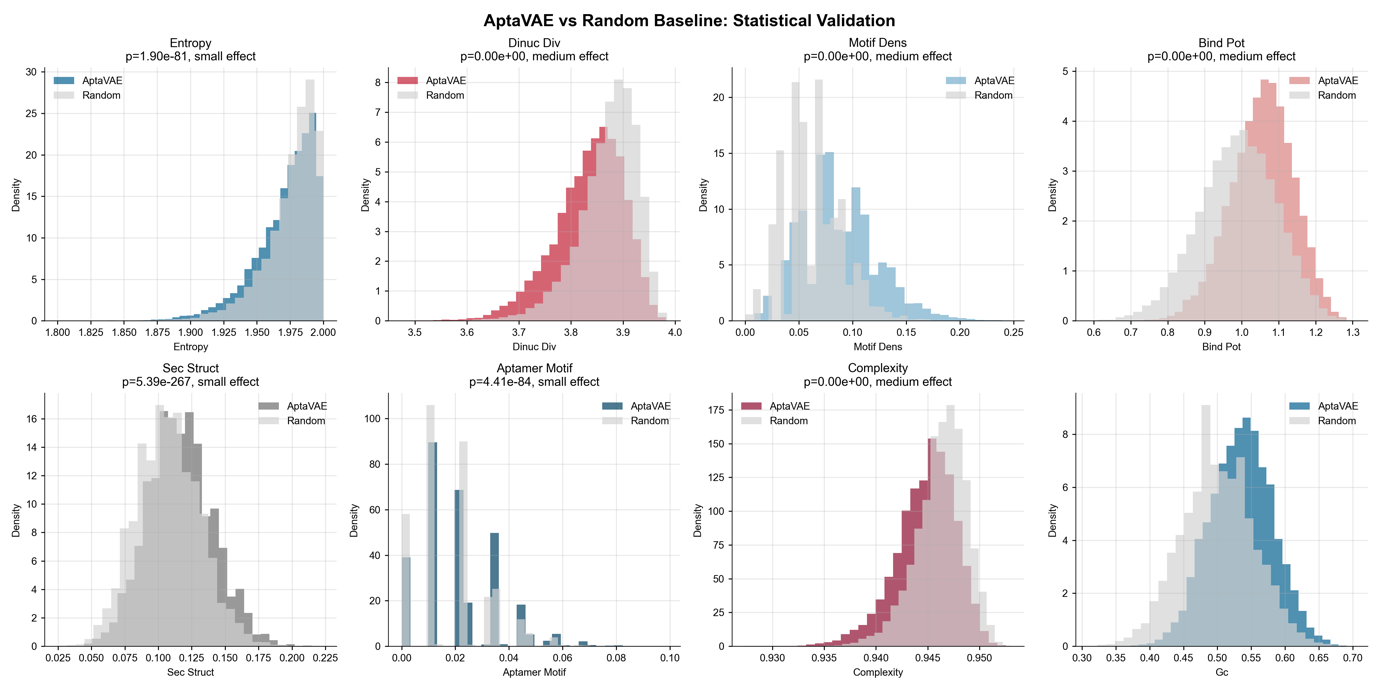


**Figure S73.** AptaVAE initial library (sampling on CS steroid target) vs mask-matched random baseline (N=10,000 each). Density histograms compare eight **initial-pool** features:

(a) **Sequence entropy:** shows higher entropy (peaking around 1.975-2.000) vs Random baseline, indicating AptaVAE generates more information-rich sequences.

(b) **Dinucleotide diversity:** unique 2-mer richness, normalized. Shows lower dinucleotide diversity (peaking around 3.8) vs Random (3.9), suggesting AptaVAE generates sequences with more structured, less random dinucleotide patterns.

(c) **Motif density:** density of common aptamer motifs like G-quadruplex patterns (GGGG) and palindromic sequences. Significantly higher motif density (peaking around 0.08-0.10) vs Random (0.04-0.05), indicating AptaVAE generates sequences with more biologically relevant structural motifs.

(d) **Binder-likeness:** Simple binding potential score based on GC content × entropy; Higher binding potential (peaking around 1.05-1.10) vs Random (0.95-1.00), suggesting AptaVAE sequences are more likely to bind targets.

(e) **Secondary-structure index**: Higher values: more potential for forming secondary structures.

(f) **Aptamer motif:** More concentrated distribution of aptamer motifs, indicating AptaVAE generates sequences with more specific, less uniform aptamer characteristics.

(g) **Sequence complexity**: Lower complexity (peaking around 0.945) vs Random (0.948-0.950), suggesting AptaVAE generates sequences with more structured, less random complexity.

(h) **GC fraction:** Higher GC content (peaking around 0.55) vs Random (0.50), indicating AptaVAE generates sequences with more stable base-pairing potential.


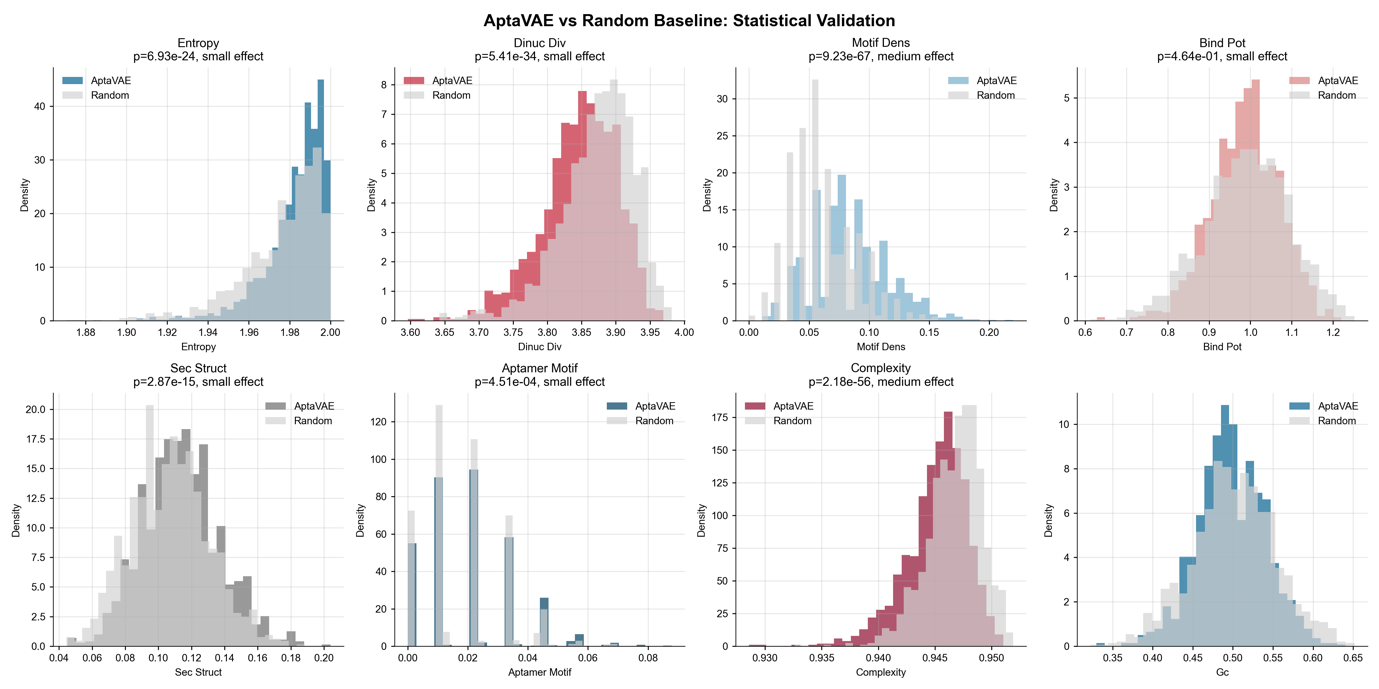


**Figure S74.** AptaVAE initial library (sampling on TES steroid target) vs mask-matched random baseline (N=10,000 each).


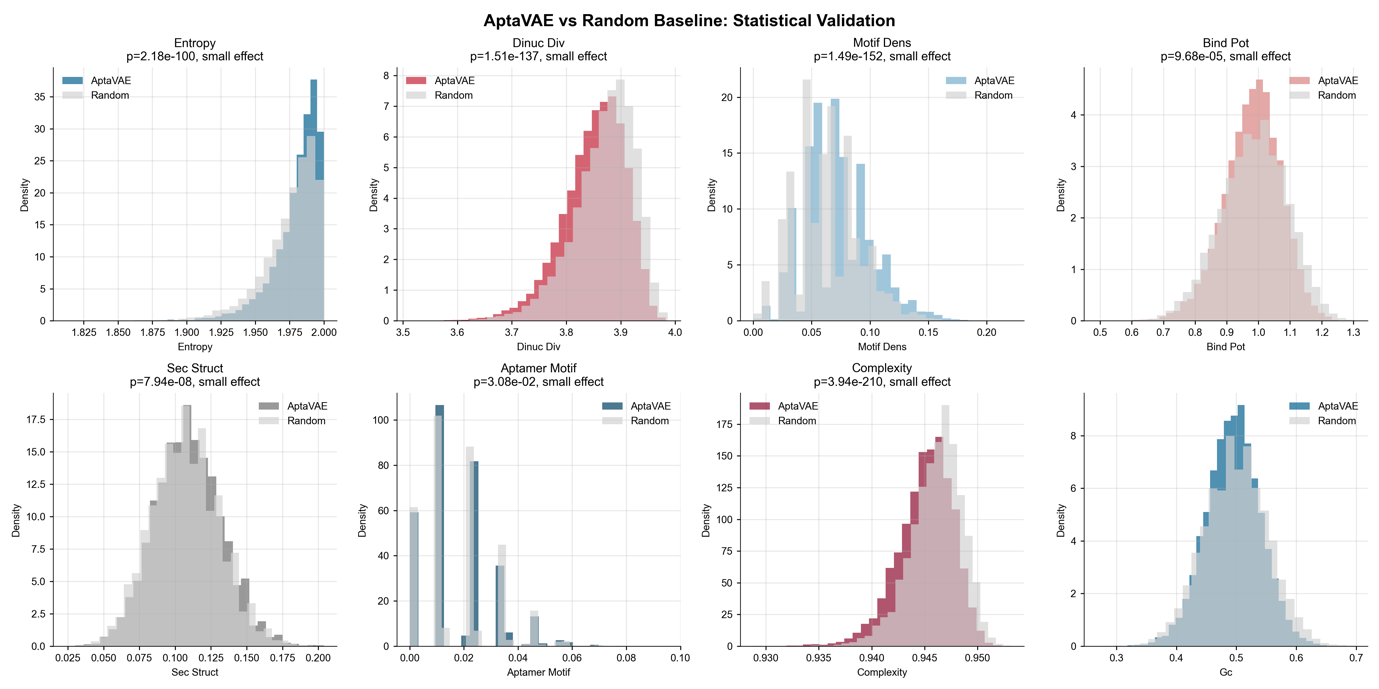


**Figure S75.** AptaVAE initial library (sampling on BE steroid target) vs mask-matched random baseline (N=10,000 each).

## Table S1 | Collected 195 Aptamer Sequences

| **Reference** | **Target** | **Reported Name** | **Aptamer Sequence** | **Bases** |
| --- | --- | --- | --- | --- |
| Yuan et al. (2020) WIPO. Patent WO2020069318 | CS | MP573335.1 | TAGGGAAGAGAAGGACATATGATGGGCCAGGGGCGTGTTATATTCCGTAGGGCTTGACTAGTACATGACCACTTGA | 76 |
|  | CS | MP573336.1 | TCAAGTGGTCATGTAGTAGTCAAGCCCTACGGAATATAACACGCCCCTGGCCCATCATATGTCCTTCTCTTCCCTA | 76 |
|  | CS | MP573337.1 | TCAAGTGGTCATGTA | 15 |
|  | CS | MP573338.1 | ATGTAGTAGTCAAGC | 15 |
|  | CS | MP573339.1 | CAAGCCCTACGGAAT | 15 |
|  | CS | MP573340.1 | GGAATATAACACGCC | 15 |
|  | CS | MP573341.1 | ACGCCCCTGGCCCAT | 15 |
|  | CS | MP573342.1 | CCCATCATATGTCCT | 15 |
|  | CS | MP573343.1 | GTCCTTCTCTTCCCTA | 16 |
|  | CS | MP573344.1 | GGAATGGATCCACATCCATGGATGGGCAATGCGGGGTGGAGAATGGTTGCCGCACTTCGGCTTCACTGCAGACTTGACGAAGCTT | 85 |
| Palma et al. (2019) WIPO. Patent WO2019102217 | CS | MP284932.1 | GGAATGGATCCACATCCATGGATGGGCAATGCGGGGTGGAGAATGGTTGCCGCACTTCGGCTTCACTGCAGACTTGACGAAGCTT | 85 |
|  | CS | MP284935.1 | AAGCTTCGTCAAGTCTGCAGTGAAGCCGAAGTGCGGCAACCATTCTCCACCCCGCATTGCCCATCCATGGATGTGGATCCATTCC | 85 |
| Martin et al. (2014) Anal. Bioanal. Chem. | CS | 15-1a | ATGGGCAATGCGGGGTGGAGAATGGTTGCCGCACTTCGGC | 40 |
|  | CS | CSS.1 | GACGACGCCCGCATGTTCCATGGATAGTCTTGACTAGTCGTC | 42 |
|  | CS | A219 | ATGGGCAATGCGGGGTGGAGAATGGTTGCCGCACTTCGGC | 40 |
|  | CS | B84 | GGGGGTTGGAAGGGGGGGCCGCGGGGTGGGCCATCGTTCG | 40 |
|  | CS | C73 | GGGGTGGTTTGGGGACCGTGGGGAGTGTGTGGAGGGGGATGAG | 43 |
|  | CS | D41 | GACGGGTTGTGGGAAGGGTGTCCCGTGAATGGGGTGC | 37 |
|  | CS |  | GTCGCGTGTGACCAGTGGTGTGTGGAGTATGGAATGGTCG | 40 |
|  | CS |  | TCGGGTACTTCCAAGACCTGGTGGGGAGTGGGAATGGTCG | 40 |
|  | CS |  | GTCGGGGCATTGCCGAGTGGTGTGGAGTTTGGAATGGTCG | 40 |
|  | CS |  | ACCGTGCGGGTGGGGAGGTTGAATCGGCCCGCGTTTCGTA | 40 |
|  | CS |  | TTGGTGTGGAGGTTGGAATGGTCCGGATGGATGGGACGTC | 40 |
|  | CS |  | CGAGGTCAGCAAGCTGTTTGGTGTGGAGTTGGAATGGTCG | 40 |
|  | CS |  | CCGCGTAAGGGTGGCTTTGGGTGGGAGTTGGGAATGGTCG | 40 |
|  | CS |  | ATGGGCAATGCGGGGTGGAGAATGGTTGCCGCACTTCGGC | 40 |
|  | CS |  | CCTGGAGGTGGAGTTGGGAATGGTTCAGGATATGAGCTTG | 40 |
|  | CS |  | CCGTTGTGCGGTGCGTGGTGGTGGAGTATGGGAATGGTCG | 40 |
|  | CS |  | GAGGGTTGGAAGGGAGGGGCCCGGGGTGGGCCATCGTTCG | 40 |
|  | CS |  | ATGGGCAATGCGGGGTGGAGAATGGTTGCCGCACTTCGGC | 40 |
|  | CS |  | TTCCAGATCATGGACGGATTTAGCCCAGTGGGTGCGCGAG | 40 |
|  | CS |  | ACACTACACGGGGTGGAGAAGGGTGGTGCCGTGTAGCTCG | 40 |
|  | CS |  | GAGGCTTGGTGGGTGCTCTTGGATGTGTGAGTATCGCGTC | 40 |
|  | CS |  | TGGATGAGGAGTGAGCTTCGTAGGATTAACTTGCTTCGGC | 40 |
|  | CS |  | CAAGCTGCCGGGGTTTGGAAGGGGTGGCTGCGTTGCGTCG | 40 |
|  | CS |  | TGACGCGGGTGGGGTTTGGGGAGTGGGCGCGTCATCGCTT | 40 |
|  | CS |  | CGGTGGGTTTGGAAGGGGGGACCGCCTATTTGCAGGAGTC | 40 |
|  | CS |  | TGGGGTTTGGGGATGGACAGGGTGGGGAGCCTGTTCTTCG | 40 |
|  | CS | 15-1 | ATGGGCAATGCGGGGTGGAGAATGGTTGCCGCACTTCGGC | 40 |
|  | CS | 15-2 | ACACTACACGGGGTGGAGAAGGGTGGTGCCGTGTAGCTCG | 40 |
|  | CS | 15-3 | GAGGGTTGGAAGGGAGGGGCCCGGGGTGGGCCATCGTTCG | 40 |
|  | CS | 15-4 | ACCGTGCGGGTGGGGAGGTTGAATCGGCCCGCGTTTCGTA | 40 |
|  | CS | 15-5 | GGGTGGTATGGGGGACCGCTCGGAGGTGGAGTAGCGTATG | 40 |
|  | CS | 15-6 | GACGGGTTGTGGGAAGGGTGTCCCGTGAATGGGGTGCGAG | 40 |
|  | CS | 15-7 | CAAGCTGCCGGGGTTTGGAAGGGGTGGCTGCGTTGCGTCG | 40 |
|  | CS | 15-8 | ATGGACAATGCGGGGTGGAGAATGGTTGCCGCACTTCGGC | 40 |
|  | CS | 15-9 | ACACTACACGGGGTGGAGAAGGGTTGTGCCGTGTAGCTCG | 40 |
|  | CS | 15-10 | ACGCGGGCTGTGTGGAGGGTTATGCGCAGCCGGGATTCGC | 40 |
| Wang et al. (2022) Sci. Adv. | CS |  | CTCTCGGGACGACCGGTCTGGGGACCCTGTCTGGGTGTGTGGGTAGTAGGTCGTCCC | 57 |
|  | CS |  | CGACCGGTCTGGGGACCCTGTCTGGGTGTGTGGGTAGTAGGTCG | 44 |
| Yang et al. (2017) ACS Chem. Biol. | DOG | DOGS.2 | CTCTCGGGACGACCCGGATTTTCCGAGTGGAACTAGCTGTGGCGGTCGTCCC | 52 |
|  | DIS | DISS.2 | CTCTCGGGACGACGGGGGTGGCATAGGGTAGGCTAGGGTCACTGTCGTCCC | 51 |
|  | DIS | DISS.3 | CTCTCGGGACGACGTGGCTAGGTAGGTTGCATGCGGCATAGGGGTCGTCCC | 51 |
|  | DIS | DISS.4 | CTCTCGGGACGACGTGACGGTGTGTAGTTGGGTTGTGGCAGGAGTCGTCCC | 51 |
|  | TES | TESS.I | CTCTCGGGACGACGGGATGTCCGGGGTACGGTGGTTGCAGTTCGTCGTCCC | 51 |
|  | TES | TESS.2 | CTCTCGGGACGACCAGGTGCCATTAGCGTCAGTGTGCTACGATGTCGTCCC | 51 |
|  | TES | TESS.3 | CTCTCGGGACGACGGGTGGTCATTGAGTGGTCTTAGGCAGGTAGTCGTCCC | 51 |
|  | CS | CSS.1 | CTCTCGGGACGACGCCCGCATGTTCCATGGATAGTCTTGACTAGTCGTCCC | 51 |
|  | CS | CSS.2 | CTCTCGGGACGACTAGCGTATGCGCCAGAAGTATACGAGGATAGTCGTCCC | 51 |
|  | CS | CSS.3 | CTCTCGGGACGACGCCAGAAGTTTACGAGGATATGGTAACATAGTCGTCCC | 51 |
|  | ALD | ALDOS.I | CTCTCGGGACGACAGATAGTTGTTCTTAGCGATGTTCAGCGTTGTCGTCCC | 51 |
|  | ALD | ALDOS.2 | CTCTCGGGACGACGGTAGGTAGGCCAACTGGGTATTTACTGGTGTCGTCCC | 51 |
| Niu et al. (2022) Sens. Diagn. | CS | 15-1a | ATGGGCAATGCGGGGTGGAGAATGGTTGCCGCACTTCGGC | 40 |
|  | CS | 15-1b | TGCGGGGTGGAGAATGGTTGCCGCA | 25 |
|  | CS | CSS.1-T | GACGACGCCCGCATGTTCCATGGATAGTCTTGACTAGTCGTC | 42 |
|  | CS | CSS.1-mt1 | GACGACACCCGCATGTTCCATGGGTAGTCTTGACTAGTCGTC | 42 |
|  | CS | CSS.1-mt2 | GACGACTCCCGCATGTTCCATGGATAGTCTTGACTAGTCGTC | 42 |
|  | CS | CSS.1-4bp | CGACGCCAGAAGTTTACGAGGATATGGTAACATAGTCG | 38 |
|  | CS | CSS.1-3bp | GACGCCAGAAGTTTACGAGGATATGGTAACATAGTC | 36 |
|  | CS | CSS.1-2bp | ACGCCAGAAGTTTACGAGGATATGGTAACATAGT | 34 |
| Jauset-Rubio et al. (2019) ACS Omega | BE | E1 | TAGGGAAGAGAAGGACATATGATGCCTGACCGGAGGCTGACCGAAGTGAGGAATTCGTACTTATTGACTAGTACATGACCACTTGAGG | 88 |
|  | BE | E2 | TAGGGAAGAGAAGGACATATGATGACAACAATGGTACTGACCGGAGGCTGACCGGAATGGGAATTACAGTTTGACTAGTACATGACCACTTGAGG | 95 |
|  | BE | E3 | TAGGGAAGAGAAGGACATATGATCCTGACCAGAGGCTGACCTAGTTTTCTCGGGAATTACTGTTTGACTAGTACATGACCACTTGAGG | 88 |
|  | BE | E4 | TAGGGAAGAGAAGGACATATGATTCCTGACCGGAGGCTGACCGAAGTGAGGAATTCGTACCTATTGACTAGTACATGACCACTTGAGG | 88 |
|  | BE | E5 | TAGGGAAGAGAAGGACATATGATCCTGACCGGAGGCTGACCTAGTTTTCTCGGGAATTACGGTTTGACTAGTACATGACCACTTGAGG | 88 |
|  | BE | E6 | TAGGGAAGAGAAGGACATATGATGCCTGACCGGAGGCTGACCGAAGTGAGGAATTCGTACCTATTGACTAGTACATGACCACTTTGAGG | 89 |
|  | BE | E7 | TAGGGAAGAGAAGGACATATGATCGTCCTGACCGTAGGCTGACCATTAAAAACTCTATTGATATTGACTAGTACATGACCACTTGAGG | 88 |
|  | BE | E8 | TAGGGAAGAGAAGGACATATGATGCCTGACCGGAGGCTGACCGAAGTGAGGAATTTGTACCTATTGACTAGTACATGACCACTTGAGG | 88 |
|  | BE | E9 | TAGGGAAGAGAAGGACATATGATCGTCCTGACCGGAGGCTGACCGAAGTGAGGAATTCGTACCTATTGACTAGTACATGACCACTTGAGG | 90 |
|  | BE | E10 | TAGGGAAGAGAAGGACATATGATAATCATGTTCTGACCGCAAGGCTGACCTAGTTTTCTCGGGAATTACAGTTTGACTAGTACATGACCACTTGAGG | 97 |
|  | BE | E11 | TAGGGAAGAGAAGGACATATGATAATCATGTTCTGACCGGAGGCTGACCGAAGTGAGGAATTCGTACCTATTGACTAGTACATGACCACTTGAGG | 95 |
|  | BE | E12 | TAGGGAAGAGAAGGACATATGATCCTGACCGCAGGCTGACCCATGCATATCGGGAATTGTACCTTGACTAGTACATGACCACTTGAGG | 88 |
|  | BE | E13 | TAGGGAAGAGAAGGACATATGATCCTGACCGGAGGCTGACCTAGCTTTCTCGGGAATTACAGTTTGACTAGTACATGACCACTTGAGG | 88 |
|  | BE | E14 | TAGGGAAGAGAAGGACATATGATCGTCCTGACCGAAGGCTGACCGAAGTGAGGAATTCGTACCTATTGACTAGTACATGACCACTTGAGG | 90 |
|  | BE | E15 | TAGGGAAGAGAAGGACATATGATAATCATGTTCTGACCGCAAGGCTGACCGAAGTGAGGAATTCGTACCTATTGACTAGTACATGACCACTTGAGG | 96 |
|  | BE | E16 | TAGGGAAGAGAAGGACATATGATCCTGACCGGAGGCTGACCTAGTTTTCTCGGGAATTACTGTTTGACTAGTACATGACCACTTGAGG | 88 |
|  | BE | E17 | TAGGGAAGAGAAGGACATATGATCCTGACCGGAGGCTGACCGAAGTGGGGAATTCGTACCTATTGACTAGTACATGACCACTTGAGG | 87 |
|  | BE | E18 | TAGGGAAGAGAAGGACATATGATGCCTGACCGGAGGCTGACCGAAGTGGGGGAATTCGTACCTATTGACTAGTACATGACCACTTGAGG | 89 |
|  | BE | E19 | TAGGGAAGAGAAGGACATATGATCGTCCTGACCGTAGGCTGACCTAGTTTTCTCGGGAATTACAGTTTGACTAGTACATGACCACTTGAGG | 91 |
|  | BE | E20 | TAGGGAAGAGAAGGACATATGATAACAACAATGGCACTGACCGGAGGCTGACCGGAATGGGAATTGACTAGTACATGACCACTTGAGG | 88 |
|  | BE | E21 | TAGGGAAGAGAAGGACATATGATGCCTGACCGGAGGCTGACCAAAGTGAGGAATTCGTACCTATTGACTAGTACATGACCACTTGAGG | 88 |
|  | BE | E22 | TAGGGAAGAGAAGGACATATGATCACAACAATGGTACTGACCGGAGGCTGACCGGAATGGGAATTGACTAGTACATGACCACTTGAGG | 88 |
|  | BE | E23 | TAGGGAAGAGAAGGACATATGATGCCTGACCGGAGGCTGACCGAAGTGAGGAATTGCGTACCTATTGACTAGTACATGACCACTTGAGG | 89 |
|  | BE | E24 | TAGGGAAGAGAAGGACATATGATAAATATGCGAAAGCGTCCTGACCGAAGGCTGACCGGAGTGGGAGTTGACTAGTACATGACCACTTGAGG | 92 |
|  | BE | E25 | TAGGGAAGAGAAGGACATATGATAATCATGTTCTGACCGCAAGGCTGACCGGAGTGGGAATTGACTAGTACATGACCACTTGAGG | 85 |
|  | BE | E26 | TAGGGAAGAGAAGGACATATGATCTCGACCGGAGGCTGACCGAAGTGAGGAATTCGTACCTATTGACTAGTACATGACCACTTGAGG | 87 |
|  | BE | E27 | TAGGGAAGAGAAGGACATATGATCCATATCCGAAGGGTCCTGACCGGAGGCTGACCGGAGTGGGAGTTGACTAGTACATGACCACTTGAGG | 91 |
|  | BE | E28 | TAGGGAAGAGAAGGACATATGATACATATCCGAAGGGTCCTGACCGGAGGCTGACCGGAGTGGGAATTGACTAGTACATGACCACTTGAGG | 91 |
|  | BE | E29 | TAGGGAAGAGAAGGACATATGATCCTGACCGGAGGCTGACCGAAATATTGTCGGGAATTACAGTTTGACTAGTACATGACCACTTGAGG | 89 |
|  | BE | E30 | TAGGGAAGAGAAGGACATATGATGACAACAATGGTACTGACCGGAGGCTGACCGGAGTGGGAATTGACTAGTACATGACCACTTGAGG | 88 |
|  | PRO | P1 | TAGGGAAGAGAAGGACATATGATACCTCCGAAGTATCATGCGAAGCATGTCCCGAATTTCATTCGTTCTCGCGACTTGACTAGTACATGACCACTTGAGG | 100 |
|  | PRO | P2 | TAGGGAAGAGAAGGACATATGATGTCTGAGGTACTCACTTCTCACGTACGTTTCCCTCTTGATTTGACTAGTACATGACCACTTGAG | 87 |
|  | PRO | P3 | TAGGGAAGAGAAGGACATATGATCAACTGAAAGCCTTGATGCGCAGCCATATCCGAAGGGTCCTTGTTAAGATAATACTTAACCTCTTGACTAGTACATGACCACTTGAGG | 111 |
|  | PRO | P4 | TAGGGAAGAGAAGGACATATGATTATATGCTCTGCGTCCTTTCTCGCAACTATATTGCTTCCCCTTGACTAGTACATGACCACTTGAGG | 89 |
|  | PRO | P5 | TAGGGAAGAGAAGGACATATGATACCTCCGAAGTATCATGCGGAGCATGTCCCGAATTTCATTCGTTCTCGTGACTTGACTAGTACATGACCACTTGAGG | 100 |
|  | PRO | P6 | TAGGGAAGAGAAGGACATATGATGTCTGAGGTACTCACTTCTCACGTACGTTTCCCTCCTGAATTGACTAGTACATGACCACTTGAGG | 88 |
|  | PRO | P7 | TAGGGAAGAGAAGGACATATGATAAATACTGGCGGGGGGTCCACTCGGATTAGGGGAAGTACCCGAAATTGATAATCTTGAGGTAGTCTTCTTGACTAGTACATGACCACTTGAGG | 116 |
|  | PRO | P8 | TAGGGAAGAGAAGGACATATGATGTGCCGTGAATACAGGCCCTTCTCCGCTCCGCGTTCCCCGTTTGACTAGTACATGACCACTTGAGG | 89 |
|  | PRO | P9 | TAGGGAAGAGAAGGACATATGATCAACTGAAAGCCTTGATGCGTAGCCATATCCGGAGGGTCCTTGTTAAGATAATACTTAACCTCTTGACTAGTACATGACCACTTGAGG | 111 |
|  | PRO | P10 | TAGGGAAGAGAAGGACATATGATGTGCCGTGAATACAGGCCCTTCTCCGCTCCGCGTTCCCTTGACTAGTACATGACCACTTGAGG | 86 |
|  | PRO | P11 | TAGGGAAGAGAAGGACATATGATAATCATGTCTCGCCTGCAGGCGACTTCTCACGTACGTTTCCCTCTTGAATTGACTAGTACATGACCACTTGAGG | 97 |
|  | PRO | P12 | TAGGGAAGAGAAGGACATATGATACCTCCGAAGTATCATGCGAAGCATGTCCCGAATTTCATTCGTTCTCGAGACTTGACTAGTACATGACCACTTGAGG | 100 |
|  | PRO | P13 | TAGGGAAGAGAAGGACATATGATACCTCCGAAGTATCATGCGAAGCATGTCCCGAATTTCATTCGTTCTCGTGATTGACTAGTACATGACCACTTGAGG | 99 |
|  | PRO | P14 | TAGGGAAGAGAAGGACATATGATGTGCCGTGAATACAGGCCCTTCTCCGCTCCGCGTTCCGTTTTTGACTAGTACATGACCACTTGAGG | 89 |
|  | PRO | P15 | TAGGGAAGAGAAGGACATATGATACCTCCGAAGTATCATGCGAAGCATGTCCCGAATTTCATTCGTTCTCGTTAATTGACTAGTACATGACCACTTGAGG | 100 |
|  | PRO | P16 | TAGGGAAGAGAAGGACATATGATTGTCCCGCCGAGGAAGGCGTTCTCAGCTGCGTACGCAACTTTCCCTTTGACTAGTACATGACCACTTGAGG | 94 |
|  | PRO | P17 | TAGGGAAGAGAAGGACATATGATGTCTGAGGTACTCACTTCTCACGTACGTTTCCCCTCTTGAATTGACTAGTACATGACCACTTGAGG | 89 |
|  | PRO | P18 | TAGGGAAGAGAAGGACATATGATGTGCCGTGAATACAGGCCCTTCTCCGCTCCGCGTTCCCCTTATTGACTAGTACATGACCACTTGAGG | 90 |
|  | TES | T1 | TAGGGAAGAGAAGGACATATGATAGCTTCATATCCTGGCAGGGTCCATCTCTGCGTAGCTCCCTTGACTAGTACATGACCACTTGAGG | 88 |
|  | TES | T2 | TAGGGAAGAGAAGGACATATGATTATATGCTCTGCGTCCCTTCTCGCAACTATATTGCTTCCCTTGACTAGTACATGACCACTTGAGG | 88 |
|  | TES | T3 | TAGGGAAGAGAAGGACATATGATTATATGCTTTGCGTCCTTTCTCGCAACTATATTGCTTCCCTTGACTAGTACATGACCACTTGAGG | 88 |
|  | TES | T4 | TAGGGAAGAGAAGGACATATGATCCTTGCCATGTTGGGACATCGTTTTACGGGCCTCTTCAGAATTACTGTTTGACTAGTACATGACCACTTGAGG | 96 |
|  | TES | T5 | TAGGGAAGAGAAGGACATATGATGTGCCGTGAATACAGGCCCTTCTCCGCTCCGCGTTCCGATTTGACTAGTACATGACCACTTGAGG | 88 |
|  | TES | T6 | TAGGGAAGAGAAGGACATATGATGTGCCGTGAATACAGGCCCTTCTCCGCTCCGCGTTCCGCTTTGACTAGTACATGACCACTTGAGG | 88 |
|  | TES | T7 | TAGGGAAGAGAAGGACATATGATTATATGCTCTGCGTCCTTTCTCGCAACTACATTGCTTCCCTTGACTAGTACATGACCACTTGAGG | 88 |
|  | BE | E11.1 | TAGGGAAGAGAAGGACATATGATAATCATGTTCTGACC GGAGGCTGACCGAAGTGAGGAATTCGTACCTATTGACT AGTACATGACCACTTGA | 95 |
|  | BE | E26.1 | TAGGGAAGAGAAGGACATATGATCTCGACCGGAGGCT GACCGAAGTGAGGAATTCGTACCTATTGACTAGTACAT GACCACTTGA | 87 |
|  | BE | E28.1 | TAGGGAAGAGAAGGACATATGATACATATCCGAAGGG TCCTGACCGGAGGCTGACCGGAGTGGGAATTGACTAGT ACATGACCACTTGA | 91 |
|  | PRO | P5.1 | TAGGGAAGAGAAGGACATATGATACCTCCGAAGTATC ATGCGGAGCATGTCCCGAATTTCATTCGTTCTCGTGACT TGACTAGTACATGACCACTTGA | 100 |
|  | PRO | P6.1 | TAGGGAAGAGAAGGACATATGATGTCTGAGGTACTCA CTTCTCACGTACGTTTCCCTCCTGAATTGACTAGTACAT GACCACTTGA | 88 |
|  | TES | T6.1 | TAGGGAAGAGAAGGACATATGATGTGCCGTGAATACA GGCCCTTCTCCGCTCCGCGTTCCGCTTTGACTAGTACAT GACCACTTGA | 88 |
| Yang et al. (2012) J. Am. Chem. Soc. | DOG |  | GGCTCTCGGGACGTGGATTTTCCACAAACGAAGTGTCCC | 39 |
|  | DOG |  | GGCTCTCGGGACGTAGGATTTTCCTACAACGAAGTGTCCC | 40 |
|  | DIS |  | GGCTCTCGGGACGTGGATTTTCCGCATACGAAGTTGTCCC | 40 |
|  | BE |  | GGCTCTCGGGACGACATGGATTTTCCATCAACGAAGTGCGTCCGTCCC | 48 |
|  | DIS |  | GGCTCTCGGGACGACGAGGATTTTTCCTCTAACGAAGTGGGTTGTCCC | 48 |
|  | DCA |  | GGCTCTCGGGACGCTGGGTTTTCCCAGGACGAAGTCCGTCCCC | 43 |
|  | DIS |  | GGCTCTCGGGACGACTCGGATTTTCCGTGAACGAAGTGGGTTGTCCC | 47 |
|  | DCA |  | GGCTCTCGGGACGACTTGGATTTTCCCTCCACGAAGTGGGTCGTCCC | 47 |
|  | DIS |  | GGCTCTCGGGACGACTTGGTTTCCATGAACGAAGTGGGTCGTCC | 44 |
|  | DIS |  | GGCTCTCGGGACGACATGGATTTTCCATTAACGAAGTGAGTTGTCCC | 47 |
|  | DIS |  | GGCTCTCGGGACGACGAGGATTCTCCTCTAACGAAGTGGGTTGTCCC | 47 |
|  | BE |  | GGCTCTCGGGACGAACCGGGATTTTCCCATTACGAAGTGAGCCGTCCC | 48 |
|  | BE |  | GGCTCTCGGGACGACATGGATTCTCCATTAACGAAGTGGGTCCGTCCC | 48 |
|  | BE |  | GGCTCTCGGGACGACATGGATTTTCCATCAACGAAGTGTGTTGTCCC | 47 |
|  | BE |  | GGCTCTCGGGACGACGAGGATTTTCCATTGACGAAGTTCCGTCCC | 45 |
|  | BE |  | GGCTCTCGGGACGCGGGGATTTTCCGCTAACGAAGTGCTCCC | 42 |
|  | BE |  | GGCTCTCGGGACGTAGGATTTTCCTACAACGAAGTGTCCC | 40 |
|  | BE |  | GGCTCTCGGGACGTGGATTTTCCGCATACGAAGTTGTCCC | 40 |
|  | BE |  | GGCTCTCGGGACGCCTGGATTTTCCGGGTACGAAGTGCGTCGTCCC | 46 |
|  | BE |  | GGCTCTCGGGACGACATGGATATTCCATCAACGAAGTGGGTCCGTCCC | 48 |
|  | BE |  | GGCTCTCGGGACGACGCGGAATTTTCCAAGAACGAAGTAATCGTCCC | 47 |
|  | BE |  | GGCTCTCGGGACGCGGGGATTTTCCGCTAACGAAGTGGGTCCC | 43 |
|  | DCA |  | GGCTCTCGGGACGACAAGGATTTTCCTAGAACGAAGTTGGTCGTCCC | 47 |
|  | DCA |  | GGCTCTCGGGACGACAAGGATTTTCCTTGGACGAAGCCTGTGTCCC | 46 |
|  | DCA |  | GGCTCTCGGGACGACCGGGATTTTCCGGACACGAAGTGGGTCGTCCC | 47 |
|  | DCA |  | GGCTCTCGGGACGACTAGGATTTTCCTGTCACGAAGTGGGTCGTCCC | 47 |
|  | DCA |  | GGCTCTCGGGACGACTGGGATTTTCCTGAAACGAAGTACGTCGTCCC | 47 |
|  | DCA |  | GGCTCTCGGGACGACTTGGATTTTCCAAAAACGAAGTGTGTCATCCC | 47 |
|  | DCA |  | GGCTCTCGGGACGACTTGGATTTTCCAGTCACGAAGTCCGTCGTCCC | 47 |
|  | DCA |  | GGCTCTCGGGACGACTTGGATTTTCCGAGAACGAAGTGTGTCGTCCC | 47 |
|  | DCA |  | GGCTCTCGGGACGAGAGGATTTTCCTCATACGAAGTCGGTCGTCCC | 46 |
|  | DCA |  | GGCTCTCGGGACGCGTGGATTTTCCTGTCACGAAGTTCGTCGTCCC | 46 |
|  | DCA |  | GGCTCTCGGGACGGCCTGGATTTTCCCGAAACGAAGTGCGTCGTCCC | 47 |
|  | DCA |  | GGCTCTCGGGACGACATGGATTTTCCGTGTACGAAGTAAGTCCC | 44 |
|  | DCA |  | GGCTCTCGGGACGACTTGGATTTTCCATGTACGAAGTATTTCGTCCC | 47 |
|  | DCA |  | GGCTCTCGGGACGCTAGGTTTCCATATACCGCAATGCGCCGTCCC | 45 |
|  | DCA |  | GGCTCTCGGGACGCTGGGATTTTCCTCAAACGAAGTGTGTCGTCCC | 46 |
|  | DCA |  | GGCTCTCGGGACGCTTGGATTTTCCAAGGACGAAGTCCGTCCC | 43 |
|  | DCA |  | GGCTCTCGGGACGGACAAGGATTTCCTTGAACGAAGTAAGTTGTCCC | 47 |
|  | DIS |  | GGCTCTCGGGACGACTGGGATTTTCCCGTCACGAAGTGTGCCGTCCC | 47 |
|  | DIS |  | GGCTCTCGGGACGCGGGGATTTTCCGCTAACGAAGTGGGTCCC | 43 |
|  | DIS |  | GGCTCTCGGGACGACACGGATTTTCCTCCGACGAAGTGAGTCGTCCC | 47 |
|  | DIS |  | GGCTCTCGGGACGACATGGATTTTCCATAAACGAAGTGGGTCGTCCC | 47 |
|  | DIS |  | GGCTCTCGGGACGACATGGATTTTCCTGGAACGAAGTGAGTCGTCCC | 47 |
|  | DIS |  | GGCTCTCGGGACGACCAGGATTTTCCTTTCACGAAGTAGGTCGTCCC | 47 |
|  | DIS |  | GGCTCTCGGGACGACCGGGATTTTCCGGTTACGAAGTGCTTCGTCCC | 47 |
|  | DIS |  | GGCTCTCGGGACGACGTGGATTTTCCACTAACGAAGTGGGTCGTCCC | 47 |
|  | DIS |  | GGCTCTCGGGACGACTTGGATTTTCCATGAACGAAGTGGGTCCGTCCC | 48 |
|  | DIS |  | GGCTCTCGGGACGCAGGGATTTTCCGTCCACGAAGTATGTCGTCCC | 46 |
|  | DOG |  | GGCTCTCGGGACGCGAGGATTTCCTCAAACAAAGTGGGTGTCCC | 44 |
|  | DOG |  | GGCTCTCGGGACGGATTTTCCTCGAACGAAGTTCGTGTCCC | 41 |
|  | DOG |  | GGCTCTCGGGACGGGGATTTTCCCCCGACGAAGTCCGTCGTCCC | 44 |
|  | DOG |  | GGCTCTCGGGACGTGGATTTTCCACTAACGCAGTGAGTCCC | 41 |
|  | DOG |  | GGCTCTCGGGACGTGGATTTTCCGCATACGAAGTTGTCCC | 40 |
|  | DOG |  | GGCTCTCGGGACGTGGGTTTTCCACAAACGAATTAGGTGTCCC | 43 |
|  | DOG |  | GGCTCTCGGGACGTTGGATTTTCCAACAACGAACTCGTGTCCC | 43 |
|  | DCA | DCAS.I | CTCTCTCGGGACGCTGGGTTTTCCCAGGACGAAGTCCGTCCCGA | 44 |
|  | DIS | DISS.I | CTGCTCTCGGGACGTGGATTTTCCGCATACGAAGTTGTCCCGAG | 44 |
|  | DOG | DOGS. 1 | CTGCTCTCGGGACGTGGATTTTCCACAAACCAGAATGGTGTCCCGAGA | 48 |
|  | BE | BES.I | CTCTCGGGACGACATGGATTTTCCATCAACGAAGTGCGTCCGTCCCG | 47 |
|  | CS | CSS.1 | GACGACGCCCGCATGTTCCATGGATAGTCTTGACTAGTCGTC | 42 |
|  | CS | 15-1 | GAATGGATCCACATCCATGGATGGGCAATGCGGGGTGGAGAATGGTTGCCGCACTTCGGCTTCACTGCAGACTTGACGAAGCTT | 84 |
|  | BE | BES.1 | GGCTCTCGGGACGACATGGATTTTCCATCAACGAAGTGCGTCCGTCCC | 48 |
| Dalirirad et al. (2020) ACS Omega | CS |  | CTCTCGGGACGACGCCCGCATGTTCCATGGATAGTCTTGACTAGTCGTCCC | 51 |

**Table S1.** 195 raw steroid aptamer sequences belonging to eight different steroid classes from published literature ranging from the year 2012 to the year 2022. Collected 195 steroid aptamers with references. Each sequence was paired with a score representing its relative binding capacity to its target molecule. Higher scores indicated a greater likelihood of binding to the corresponding target. Scores were either directly obtained from the reported studies or calculated based on the relative frequency of the sequences in HT-NGS results from the specified SELEX round. Sequences appearing more frequently in the NGS data were assigned higher relative scores. Known high-affinity binders were assigned a maximum score of 0.99 to prevent loss function divergence during model training. Note: the counts and the sequencing round are not included in this table, can be found in more detail in the project GitHub repository (<https://github.com/zibin-zhao/DL-SELEX>). Target abbreviations can be found in the chemical structure notes (Table S7).

## Table S2 | AptaVAE Latent Dimension Comparison Evaluation

| **Dimension​** | **ARI​** | **AMI​** | **FM​** |
| --- | --- | --- | --- |
| **256** | 0.2332 | 0.3385 | 0.3312 |
| **128** | 0.2503 | 0.3664 | 0.3455 |
| **64** | 0.4005 | 0.5339 | 0.4808 |
| **32** | 0.6383 | 0.7473 | 0.6886 |
| **16** | 0.6919 | 0.7978 | 0.7368 |
| **8** | 0.8814 | 0.9354 | 0.8995 |

**Table S2.** Performance metrics of clustering results across different latent space dimensions. The table summarizes the Adjusted Rand Index (ARI), Adjusted Mutual Information (AMI), and Fowlkes-Mallows Index (FM) values for latent dimensions ranging from 256 to 8. As the dimensionality decreases, clustering performance improves across all metrics, with the highest scores observed at a latent dimension of 8 (ARI: 0.8814, AMI: 0.9354, FM: 0.8995). This trend suggests that lower-dimensional latent spaces capture more distinct clustering features, enhancing the separation and interpretability of the data.

## Table S3 | AptaVAE Generated Steroid Pre-Defined Library

| **Target** | **Sequence** | **Bases** |
| --- | --- | --- |
| CS | GGGNCGNCNNGGATTTTNTNNGCNCAANNTGGNTNNNNNNNNNNNN | 46 |
| TES | GGGACGACNGGNNTTTNNNNNNNNCNANGTGNNNCGTCCNCNTCCNNNNNNNNNNNNNNNNNNNNN | 66 |
| BE | GGGACGNCNGGNATTTNCCNNNNNCNAANTGNGTCNNCCCNNNNNNNNNNNNNNNNNN | 79 |
| PRO | GGGANGACNAGGATNTTCCNNNNACGNAGTGNGTCNNNCCCNNNNNNNNNNNNNNNNNNNNNNNNN | 68 |
| DIS | GGGNCGACNNGGATTTTCCCNNNACNAAGTGGGNNNNNNTCNNNCCNNNNNN | 52 |
| DCA | GGGACGACNNGGATTTTCCNNNNACNANGTGGGTCGTCCCNNNNNN | 51 |
| ALD | GGGACGACNGGNATTTTNNCTNANCNANGTGGNNCGTCCNCNTCCC | 46 |
| DOG | GGGACGACGTGTTTTTNCCAAANACGGAGTNNGTNNTCNCCGTCCC | 46 |
| **COM** | GGGACGACNNGNATTTTCCNNNNNCNANGTGNGNCNNCCNCNNNNN | 46 |

**Table S3.** ‘COM’ represents the steroid common library with confined bases of all eight steroids over 50% similarity. The other abbreviations are in chemical structure notes (Table S7).

## Table S4 | Summary Table of Evaluation on Frequency and Enrichment with FRET

| **ID** | **Sequence** | **R7_counts** | **R8_counts** | **R7_freq** | **R8_freq** | **Enrichment** | **Binding?** |
| --- | --- | --- | --- | --- | --- | --- | --- |
| **Top Frequency** | | | | | | |  |
| **3** | GCGTAGCGGCGGATTCCGATTATTCACCAAGT | 2344696 | 1751419 | 0.2451 | 0.1854 | 0.7556588 | **Yes** |
| **4** | GCGTAGCGGAGTTTTCGATTTCGATGCCAAAGT | 867652 | 329843 | 0.09071 | 0.03491 | 0.3845784 | No |
| **57** | GCGTAGCGTTTTTCCAGATTGTTTATCAAGT | 146829 | 155355 | 0.01535 | 0.01644 | 1.0703787 | No |
| **Top Enrichment** | | | | | | |  |
| **344238** | GCGATAGCGGTGTTGATTACATTACCATCAGT | 0 | 28 | 0 | 0.00000296 | 29.337303 | No |
| **339126** | GCGAGTCGTCAGTTACGATTTCCTATTTAGT | 0 | 26 | 0 | 0.00000275 | 27.314041 | No |
| **372064** | GCGAACTGTCGATTAGTTAGATTCCCAAGT | 0 | 25 | 0 | 0.00000265 | 26.30241 | No |

**Table S4.** Summary table of the FRET experiment in assessing the frequency and enrichment as the scoring function. The results shown in the binding column depict that, the top frequency and top enrichment are not always the true binder from the HT-NGS data. No clear relationship between binding ability to the top frequency or top enrichment.

## Table S5 | AptaClux Direct Bases Edit Distance Comparison

| **Top Hits** | **R5** | | **R3** | |
| --- | --- | --- | --- | --- |
|  | **AptaClux** | **Conventional** | **AptaClux** | **Conventional** |
| **100** | 8.8 | 10.95 | 9.45 | 11.35 |
| **1,000** | 9.135 | 11.254 | 9.324 | 11.814 |
| **10,000** | 9.1038 | 11.2273 | 9.4067 | 11.8004 |

**Table S5.** Comparison of top hit percentages for aptamer selection approaches between AptaClux (this work) and conventional methods across different dataset sizes. The table presents performance metrics for R5 and R3 regions with top hits evaluated at thresholds of 100, 1,000, and 10,000 sequences. AptaClux demonstrates consistently higher efficiency in identifying top hits within smaller datasets (e.g., 100 sequences: R5 = 8.8%, R3 = 9.45%) compared to conventional methods (e.g., 100 sequences: R5 = 10.95%, R3 = 11.35%), showcasing its potential for rapid and effective selection in high-throughput scenarios.

## Table S6 | Summary Table of Affinity (K_D_) Results from ITC

| **Unit (****μM)** | **TES** | **CS** | **BE** | **DHEA** | **CHO** | **PRO** |
| --- | --- | --- | --- | --- | --- | --- |
| **CS-R5MP-T** | 232 | 2.41 | 8.73 | 31.2 | 21.8 | 12.9 |
| **CS-R7MP-T** | 30.8 | 11.1 | 37 | 29.2 | 16.9 | 14.7 |
| **AI-CS-R3CC-****T** | 5.14 | 0.567 | 70.9 | 57 | 213 | No binding |
| **AI-CS-R5CC-T** | 108 | 1.03 | 7.48 | 134 | 170 | No binding |
| **AI-TES-R3R5HD-T** | 0.323 | 22.1 | 6.22 | 217 | 103 | 9.38 |
| **TES-R7MP-T** | 1 | 224 | 32.6 | 422 | 152 | 15.6 |
| **CS-R5MP** |  | 3.88 | NA | NA | NA | NA |
| **CS-R7MP** |  | 28.5 | NA | NA | NA | NA |
| **AI-CSR3CC** |  | 0.892 | NA | NA | NA | NA |
| **AI-CSR5CC** |  | 2.78 | NA | NA | NA | NA |
| **AI-TES-R3R5HD** | 2.58 |  | NA | NA | NA | NA |
| **TES-R7MP** | 2.49 |  | NA | NA | NA | NA |
| **NGS-2** |  | 3.73 | NA | NA | NA | NA |
| **CS-Reported** |  | 12.5 | NA | NA | NA | NA |
| **TES-Reported** | 146 |  | NA | NA | NA | NA |

**Table S6.** Summary of Isothermal Titration Calorimetry (ITC) results for all aptamer sequences generated through DL-SELEX, tested against various steroid targets. The table highlights the affinity binding (K_D_ values in µM) for each aptamer and its corresponding steroid target, with values marked in red to indicate the strongest binding specificity. Entries labelled as "No binding" denote cases where the affinity could not be determined experimentally due to insufficient interaction. The results demonstrate the effectiveness of DL-SELEX in generating aptamers with high specificity toward their intended steroid targets while also assessing cross-reactivity with non-target steroids.

## Table S7 | Steroids Chemical Formula and SMILES Representation

| **Steroid** | **Formula** | **SMILES (Canonical)** | **Source** |
| --- | --- | --- | --- |
| CS | C21H30O5 | CC12CCC(=O)C=C1CCC3C2C(CC4(C3CCC4(C(=O)CO)O)C)O | https://pubchem.ncbi.nlm.nih.gov/compound/Cortisol |
| CCS | C21H30O4 | CC12CCC(=O)C=C1CCC3C2C(CC4(C3CCC4C(=O)CO)C)O | https://pubchem.ncbi.nlm.nih.gov/compound/5753 |
| DIS | C19H30NaO6S | CC12CCC3C(C1CCC2=O)CC=C4C3(CCC(C4)OS(=O)(=O)O)C.O.[Na] | https://pubchem.ncbi.nlm.nih.gov/compound/16072213 |
| BE | C18H24O2 | CC12CCC3C(C1CCC2O)CCC4=C3C=CC(=C4)O | https://pubchem.ncbi.nlm.nih.gov/compound/5757 |
| PRO | C21H30O2 | CC(=O)C1CCC2C1(CCC3C2CCC4=CC(=O)CCC34C)C | https://pubchem.ncbi.nlm.nih.gov/compound/5994 |
| DCA | C24H39NaO4 | CC(CCC(=O)[O-])C1CCC2C1(C(CC3C2CCC4C3(CCC(C4)O)C)O)C.[Na+] | https://pubchem.ncbi.nlm.nih.gov/compound/23668196 |
| DOG | C27H40O8 | CC12CCC3C(C1CCC2C(=O)COC4C(C(C(C(O4)CO)O)O)O)CCC5=CC(=O)CCC35C | https://pubchem.ncbi.nlm.nih.gov/compound/630295 |
| TES | C19H28O2 | CC12CCC3C(C1CCC2O)CCC4=CC(=O)CCC34C | https://pubchem.ncbi.nlm.nih.gov/compound/6013 |
| ALD | C21H28O5 | CC12CCC(=O)C=C1CCC3C2C(CC4(C3CCC4C(=O)CO)C=O)O | https://pubchem.ncbi.nlm.nih.gov/compound/5839 |

**Table S7.** ‘CCS’ represents corticosterone with one less ‘-O’ oxygen than CS (hydrocortisone). For comparison purposes recorded in this paper for clarity.

## Table S8 | Aptamer Sequences Used in This Work

|  | **Sequence** |
| --- | --- |
| **CS-R5MP-T** | cgggacgacGGGGATTTTTGGTGCACTATGTGGTTCGTTCGGGGTGGGctgcgttctccattctgg |
| **CS-R7MP-T** | cgggacgacGGGGATTTTATTTGCACTATGTGGCTCGTTCGGGGTGTGctgcgttctccattctgg |
| **AICSR3CC-T** | tcgggacgacGGGGATTTTATTTGCACTAGGTGGGTCGGTCTTTTTTTGctgcgttctccattctgg |
| **AICSR5CC-T** | tcgggacgacGGGGATTTTGGTTGCACTATGTGGGTCGGTCTTTTTGGGctgcgttctccattctgg |
| **AI-TESR3R5HD-T** | ggacgacGGGGATTTGGGGTTTTCTATGTTTTTCGTCCTTTTTTTGctgcgttctccattctgg |
| **TES-R7MP-T** | ggacgacGTGGATTTTGATGTTTCTATGTTTTTCGTCCATGTCGGCctgcgttctccattctgg |
| **CS-R5MP** | ggctctcgggacgacGGGGATTTTTGGTGCACTATGTGGTTCGTTCGGGGTGGGctgcgttctccattctgg |
| **CS-R7MP** | ggctctcgggacgacGGGGATTTTATTTGCACTATGTGGCTCGTTCGGGGTGTGctgcgttctccattctgg |
| **AI-CSR3CC** | ggctctcgggacgacGGGGATTTTATTTGCACTAGGTGGGTCGGTCTTTTTTTGctgcgttctccattctgg |
| **AI-CSR5CC** | ggctctcgggacgacGGGGATTTTGGTTGCACTATGTGGGTCGGTCTTTTTGGGctgcgttctccattctgg |
| **AI-TESR3R5HD** | ggctctcgggacgacGGGGATTTGGGGTTTTCTATGTTTTTCGTCCTTTTTTTGctgcgttctccattctgg |
| **TES-R7MP** | ggctctcgggacgacGTGGATTTTGATGTTTCTATGTTTTTCGTCCATGTCGGCctgcgttctccattctgg |
| **NGS-2** | GCTGCAGCGTAGTCGGCCATTCGATTCATTTCACAAGTTGCAGC |
| **CS-Reported** | CGACCGGTCTGGGGACCCTGTCTGGGTGTGTGGGTAGTAGGTCG |
| **TES-Reported** | TAGGGAAGAGAAGGACATATGATGTGCCGTGAATACAGGCCCTTCTCCGCTCCGCGTTCCGCTTTGACTAGTACATGACCACTTGAGG |

**Table S8.** Aptamer sequences used in this work. Lower case letter represents the primer used for this aptamer.

## Table S9 | CS Panel Aptamers for DL Models Performance Comparison

| **ID** | **Name** | **sequence** | **Target** | **Label (K=CS)** | **Kd_vs_CS (µM)** | **Source_round** |
| --- | --- | --- | --- | --- | --- | --- |
| **0** | CS-R5MP-T | GGGGATTTTGGTTGCACTATTTGGGTCGGTCTTTTTGGG | CS | 1 | 2.41 | R5 |
| **1** | AI-CSR5CC-T | GGGGATTTTGGTTGCACTATGTGGGTCGGTCTTTTTGGG | CS | 1 | 1.03 | R5 |
| **2** | AI-TESR3R5HD-T | GGGGATTTGGGGTTTTCTATGTTTTTCGTCCTTTTTTTG | TES | 0 | 22.1 | R5 |
| **3** | TES-R7MP-T | GTGGATTTTGATGTTTCTATGTTTTTCGTCCATGTCGGC | TES | 0 | 224 | R7 |
| **4** | AI-TES-R3CC | GGGGATTTGGGGGGTTCTATGTTTTTCGTCCTTTTTTTG | TES | 0 | na | R3 |
| **5** | AI-TES-R5CC | GGGGATTTGGGGGGTTCTAGGTTTTTCGTCCTTTTTTTG | TES | 0 | na | R5 |
| **6** | AI-TES-R7CC | GGGGATTTGGGGTTGTCTATGTTTTACGTCCTTTTTGTG | TES | 0 | na | R7 |
| **7** | AI-TES-R7HD | GGGGATTTCGGGACGTCTATGTTTTACGTCCTTTTTTTG | TES | 0 | na | R7 |

**Table S9.** CS panel aptamers for comparing DL model performance.

## Note S1 | Simplified and Standardized Materials and Workflow for Selection Experiment in DL-SELEX

qPCR of the library: The initial library preparation employs qPCR with Premix Taq (1x), forward and reverse primers (50 nM each), and template (1 nM–1 pM). SYBR Green (1x) is used for fluorescence detection. The reaction is set to a final volume of 20 µL, with PCR cycling conditions of 98°C for 30 seconds, followed by 30 cycles of 98°C for 10 seconds, 55°C for 10 seconds, and 72°C for 3 seconds. A melting curve analysis is performed from 50°C to 95°C.

Annealing of the initial library for capture library (1st Round): The library and biotinylated capture strand (100 pmol each) are mixed with Buffer 3.1 (1x) in a 50 µL reaction volume. The annealing protocol involves denaturation at 95°C for 10 minutes, slow cooling to 25°C (0.5% ramp), and incubation at 25°C for 10 minutes to ensure stable hybridization.

Selection process: streptavidin-coated agarose resin (GeneScript, China) is used to immobilize the biotinylated capture strand. For each experiment, 200 μL of the resin is loaded onto the gravity mini column (Bio-Rad, USA). The resin is washed thoroughly with PBS (5–6 volumes) before loading the annealed library. The library (annealing product, diluted to 200 μL with PBS) is incubated with the resin for 10 minutes, and the flow-through is collected and reloaded to maximize capture efficiency. The resin is washed 10 times with PBS to remove unbound sequences. For counter-selection, a counter-ligand mix (100 µM each) is optionally applied, and the flow-through is collected. Finally, the target ligand (100 µM, 200 µL) is introduced, and the binding sequences are eluted.

Asymmetric PCR (asPCR) and library regeneration: The selected sequences are first analyzed with qPCR, as mentioned above. The qPCR products are then column purified. The purified qPCR products are amplified using asPCR with Premix Taq (2x), forward primer (200 nM), and reverse primer (20 nM) for generation of single-stranded DNA library for next round selection. The column-purified PCR product was diluted to a final concentration of 0.1 ng/µL in a 50 µL asPCR reaction volume. PCR cycling conditions include 98°C for 30 seconds, followed by 30 cycles of 98°C for 10 seconds, 55°C for 10 seconds, and 72°C for 3 seconds. Four reactions (4 × 50 µL) are prepared per iteration.

Annealing for subsequent rounds: For subsequent selection rounds, the asPCR product (200 µL) is annealed with 100 pmol of the capture strand (1 µL from a 100 µM stock solution) for target binding. The 200 µL product would be loaded directly into the column for the next selection round.

*Fluorescent resonance energy transfer (FRET) for target-induced dissociation (TID) pre-assay for aptamer candidates*

The fluorescent resonance energy transfer (FRET) experiment was conducted as a pre-assay to characterize the target-induced dissociation (TID) capacity of all aptamer sequences using a fluorescent readout. The system consisted of two components: a BHQ1-labeled quencher as the capture oligonucleotide and a FAM-labeled aptamer sequence. Upon target binding, the target displaced the capture strand annealed to the aptamer, resulting in increased fluorescence. The first step involved determining the dissociation constant between the capture strand and the aptamer to achieve an ~80–90% quenching effect, essential for the competitive assay. The dissociation constant was assessed through serial dilutions of the capture strand, starting at 500 nM, with a fixed aptamer concentration of 50 nM, including positive control. A detailed flowchart is provided in Supplementary Fig. S17. Target concentrations were tested at ratios of 2×, 20×, 200×, 1000×, 2000×, and 20,000× relative to the 50 nM aptamer concentration. The aptamer and its optimal capture strand ratio were annealed on a PCR machine with a program the same as the annealing for selection mentioned above Target solutions were mixed with equal volumes of the annealed oligonucleotide solution and incubated at room temperature in the dark for 40 minutes. Fluorescence was measured in a black 96-well plate using a Varioskan™ LUX multimode microplate reader with FAM excitation/emission wavelengths of 493/520 nm.

*Binding assay using isothermal calorimetry (ITC) for aptamer candidates*

The binding affinity (KD) of all aptamer candidates was measured using a Malvern Panalytical MicroCal PEAQ-ITC. Before the assay, aptamers were dissolved and diluted in a standard 1× PBS solution, while steroid targets were prepared by dissolving them in 100% ethanol and subsequently diluting them in the same PBS buffer. All ITC results were generated using the machine-associated MicroCal PEAQ-ITC Analysis software. We employed a one-site fitting model, which is the most commonly used model for calorimetry experiments. Confidence intervals for N (sites), K_D, and ΔH were automatically determined by the software’s default algorithm using preset upper and lower bounds; these represent estimated confidence intervals reported by the MicroCal software.

To ensure clean binding-affinity estimates for each ssDNA aptamer sequence, we adjusted the initial concentrations for each aptamer–target pair, starting from [target] = 200 µM and [aptamer] = 20 µM. After multiple trials, our goal was to achieve a stoichiometry close to one binding site for the aptamer–target complex. We also performed essential control experiments to account for heat-of-dilution effects. For each aptamer sequence, the following controls were run: (1) titrant → buffer: target (in ethanol-diluted PBS) injected into PBS; (2) buffer → sample: PBS injected into aptamer (in PBS); (3) buffer → buffer: ethanol injected into PBS; and (4) vehicle → sample: ethanol injected into aptamer (in PBS).

**Buffer condition selection rationale:**

The selection and characterization experiments were conducted under identical conditions: 25°C in phosphate-buffered saline (PBS) without magnesium or calcium ions. It is well-established that divalent metal ions like magnesium and calcium ions significantly enhance the stability of the secondary structure of aptamer and further benefit the binding process (44). In this platform, we aim to create a more generalized and flexible experimental framework for generating high-affinity aptamers under less restrictive conditions. Hence, the body fluid-like PBS with low ionic strength was used in this work.

**Overall workflow:**

**
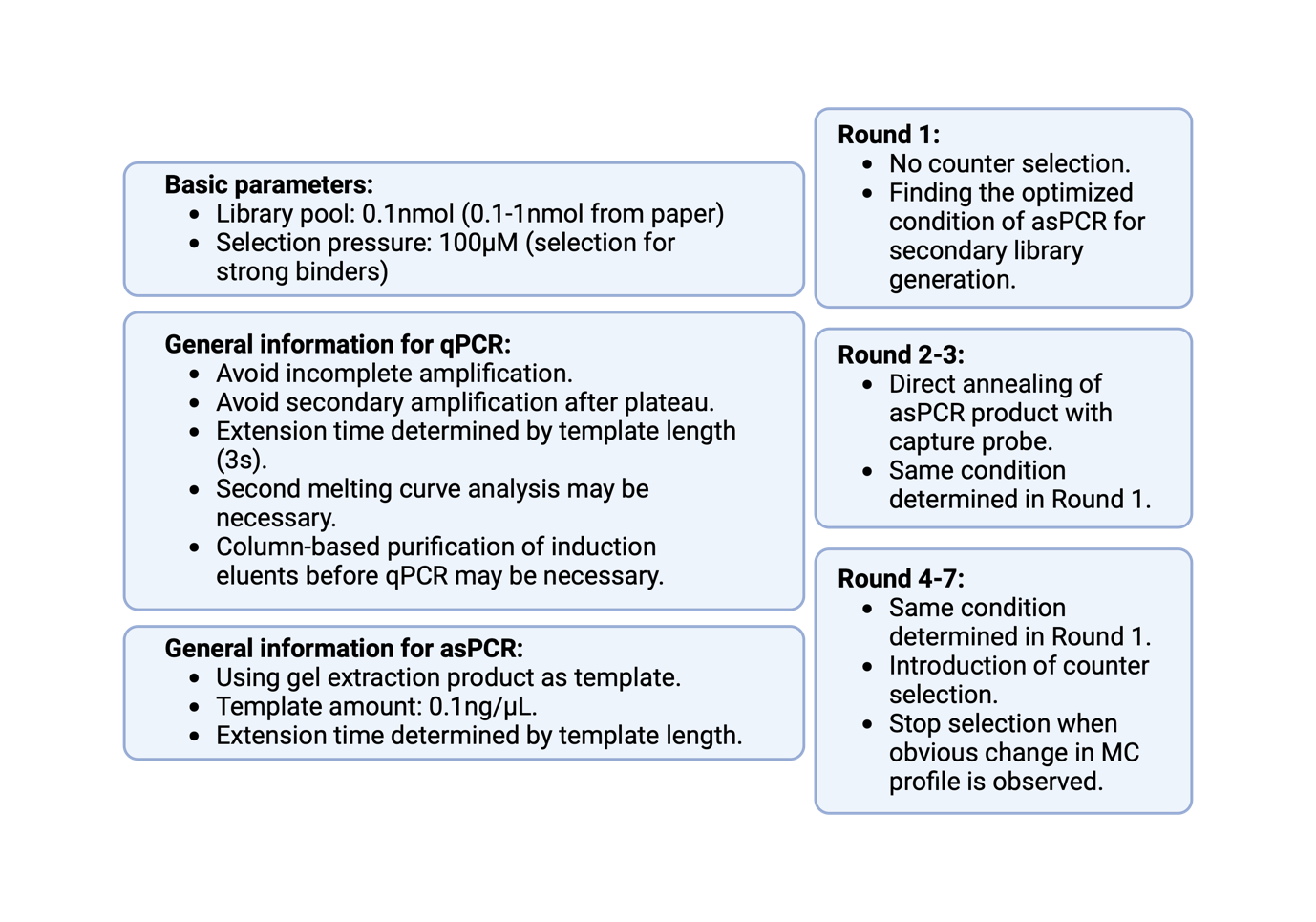
**

1. qPCR of library

|  | Final Amount |
| --- | --- |
| Premix Taq 2x | 1x |
| Forward primer | 500 nM |
| Reverse primer | 500 nM |
| Template | 1 nM ~ 1 pM/  100 times dilution of selection product. |
| SYB 100x | 1x |
| ddH_2_O | Add to final volume 20 μL |

PCR settings:

1. 98 ℃ for 30 s
2. 98℃ 10 sec; 55℃ 10 sec; 72℃ 3 sec; 30 cycles
3. melting curve 50 °C to 95 °C
4. Annealing for capture library for 1^st^ round selection

|  | Final Amount |
| --- | --- |
| Library | 2 μM |
| Capture strand | 2 μM |
| Buffer 3.1 | 1 x |
| ddH_2_O | Add up to 50 μL as final volume |

PCR settings: 95℃ 10min, Ramp 0.5% to 25 ℃，25℃ 10min

1. Selection
2. Take 200 μL of streptavidin-coated agarose resin to the column. Rinse it with 1 volume of PBS each time, rinse for 5-6 times.
3. Load the annealed library. If the volume is less than 200 uL, add PBS to make it 200 μL. Incubate the resin with the annealed library for 10 minutes. Flow the column and collect flow through.
4. Reload the flow-through from the last step. Flow the column and discard the flow through.
5. Wash the column with 1 volume of PBS for 10 times. Collect the last flow though.
6. (optional but recommended, counter-selection) Load the counter ligand mix (100 μM each), and Collect the flow through. Wash the column with PBS for 2 times.
7. Load target ligand (200 μL 100uM) to the column. Collect the flow through.
8. asPCR of selection product regeneration of library

|  | Final amount | Note |
| --- | --- | --- |
| Premix Taq 2x | 1x |  |
| Forward primer | 200 nM |  |
| Reverse primer | 20 nM |  |
| template | 0.1 ng/μL | Column purified PCR product, gel extraction if necessary. |
| ddH_2_O | Add to final volume 50 μL |  |

Each time, prepare 4x50 μL of reaction.

PCR settings:

1. 98 ℃ for 30 s
2. 98℃ 10 sec; 55℃ 10 sec; 72℃ 3 sec; 30 cycles
3. Annealing for 2^nd^ and above round of selection:

|  |  | Volume |
| --- | --- | --- |
| asPCR product |  | 200 μL |
| Capture strand | 100 pmol | 1μL from 100μM stock |

## Note S2 | Naming Convention for DL-SELEX generated Aptamer Sequences

The naming convention used in this figure and the rest of this study for aptamers is as follows: “AI” indicates our two-step DL-SELEX-generated aptamer, while aptamers without "AI" refer to those generated using the one-step AptaVAE with conventional NGS analysis using AptaSuite (39). “CS” and “TES” represent the cognate ligands of the aptamers (hydrocortisone and testosterone, respectively). “Rx” denotes aptamers derived from round “x” NGS data, e.g. “R3” is round 3 NGS data, “CC” represents the clustering method of cluster consensus of AptaClux, and “HD” represents the high-density clustering method of AptaClux. “MP” represents the most probable sequence from the conventional analysis, and “-T” indicates aptamers truncated using the global structural truncation program designed for this study.

## Note S3 | Selection Biases Discussion

We suggest that the phenomenon of why CS-R7MP-T is not the one with the highest affinity and specificity. This is likely due to selection biases that emerged in the later rounds of SELEX, where the conserved pool of aptamers became increasingly dominant. This phenomenon will be discussed in more detail in the discussion section. Additionally, the decrease in specificity observed in the later SELEX rounds can be attributed to the fact that the library was designed based on the shared characteristics of all steroids. As the rounds progressed, aptamers that were commonly selected began to dominate, favoring general steroid structures over the specific target. This highlights the effect of the broader steroid library on the selection process.

For example, an aptamer from round 7 (CS-R7MP) showed worse performance than those from rounds 3 or 5 (Fig. 3a). A possible explanation for this is the accumulation of PCR bias, where PCR amplifies multiple products unevenly, favoring certain sequences over others. This bias can arise from intrinsic differences in amplification efficiency or from self-annealing of the most abundant sequences, which can inhibit further amplification in later stages (46). In the early rounds of selection, the enrichment of high-affinity sequences dominates the library evolution, making these sequences progressively more prevalent. However, in later rounds, while affinity sequences continue to be enriched, PCR bias increasingly influences the library’s composition. As a result, further increasing the number of rounds may disproportionately amplify sequences with a preference for PCR, even if they have lower affinity. This can reduce the diversity of the binding events, potentially hindering the selection of high-performance aptamers (46).

## Note S4 | Model Assumption and Primer Refinement Discussion

*Data selection and assumptions*

Deep learning models require sufficient and relevant data to function effectively. We began with steroids due to their similar backbone of four fused carbon rings. As mentioned, the primary assumption of our model is that the common structural backbone within the steroid family corresponds to specific aptamer binding sites in a higher-dimensional space, which can be learned by the model’s latent space. The distribution of collected pairs was uneven (Supplementary Fig. S66). To test the model’s ability to handle data imbalance, we chose hydrocortisone (CS), the most abundant aptamer, and testosterone (TES), the nearest least abundant, as case studies.

*Primer refinement*

Aptamers with primer binding sites are essential for the SELEX process. However, these primer sequences can contribute to non-specific binding, increasing the likelihood of false positives (26). During pre-processing, it became evident that primers from different sources contained redundant information tailored to specific research environments. Since primers could impact model accuracy, we developed an automated primer refinement program for processing steroid aptamers. The program truncated bases at the point where the global secondary structure of each sequence was disrupted, preserving structural integrity while minimizing redundant primer data (Supplementary Fig. S2).

## Note S5 | Binding Position Prediction Discussion

Interestingly, while the binding positions aligned with the predicted regions, they did not always correspond to identical bases. This suggests that binding interactions may not be restricted to a single binding point. This observation is consistent with findings in our previous work (45), which demonstrated that ligand-aptamer interactions are not always driven by the lowest energy landscape. Instead, metastable conformations often play a significant role in binding, highlighting that meta-states of ligand-aptamer interactions can contribute more to binding efficacy than a fixed mode. As a result, manual design libraries may lack the necessary complexity to produce high-affinity aptamers or effectively advance biosensing applications.

## Note S6 | Ground Truth Sequence Selection Discussion

An important consideration is the selection of ground truth for model evaluation. It would be unreasonable to use the reported hydrocortisone (CS) or testosterone (TES) aptamers as ground truth because they were not selected under the same experimental conditions, and their sequence quality is inferior to the final-round aptamers selected in our experiments. Instead, we selected the later-round experimentally validated aptamers as the ground truth for earlier-round aptamer evaluation. This approach assumes that a later-round ground truth sequence must have been present in earlier rounds, as dictated by evolutionary progression in SELEX. Comparing within the same round does not offer meaningful insights into predictive ability. Thus, we compared the model-generated aptamers from early rounds to the validated binders from later rounds. The cluster center was assumed to represent the consensus sequence within the latent space, with earlier identification of the ground truth indicating better model performance.

## Note S7 | Molecular Dynamics and Simulation Reveal Key Binding Sites

For example, predicted binding regions for hydrocortisone (CS) and testosterone (TES) matched experimental findings closely. MD simulations for CS aptamer CS-R5MP identified accurate interactions at bases 36G – 39G, validating the accuracy of AptaVAE. Similarly, truncated aptamers like AI-TES-R3R5HD-T demonstrated improved binding due to identified critical stem-loop regions. These results underscore AptaVAE's effectiveness in accurately identifying aptamer-ligand interactions. Although experimental validation is time-intensive, computational simulations offer valuable preliminary insights into binding interactions by capturing multiple dynamic states (45). Binding interactions often involve metastable conformations rather than a fixed mode, highlighting the complexity and need for deep-learning models capable of capturing these relationships.

## Note S8 | Design Logic and Evaluation Protocol for DL Model Comparison (AptaClux, AptaDiff, RaptGen)

**Datasets and labels**

- Training round: CS R3 (39-nt variable region; adapters disabled).
- Evaluation set: labeled CS panel of 8 sequences (2 CS binders, 6 CS non-binders; TES sequences used as negatives; all labels ITC-validated).

**Identifiers.** Each sequence has a stable seq_id (or panel_id) and raw 39-nt sequence; secondary structure is represented as dot-bracket for AptaClux (to match the model’s training input).

**Preprocessing (identical across methods)**

- 39-nt variable region; adapters removed/disabled during import.
- For AptaClux: input features are [seq one-hot (4×39)] + [dot-bracket one-hot (3×39)] → 273-D.
- For AptaDiff and RaptGen: use each repository’s standard VAE encoder to obtain a latent mean μ per sequence (dimensionality may differ by model (default was employed); we always use the full μ vector the repo outputs).

For ranking metrics, we need one scalar per sequence. Rather than redesigning any model, we read out a scalar from the existing representation in a way that is symmetric for all three models:

1. Train each model’s encoder on CS-R3 only.
2. Encode all R3 sequences → latent means μ₁,…,μₙ.
3. Fit a Gaussian Kernel Density Estimator (KDE) on these μ’s (bandwidth selected by 5-fold CV on R3 only; no labels used).
4. For any evaluation sequence (x) with latent mean μ(x), define the score:

$$score\left( x \right)=-logp_{KDE\left( CS_{R3} \right)}(\mu\left( x \right))$$

Assumption here: Higher score = rarer under the R3 latent distribution ⇒ treated as more binder-like.

Note: For AptaClux we have also considered negative ELBO as an alternative scalar, but we standardize on the KDE score across all models to keep the comparison single-protocol.

**Training specifics (encoders only)**

- AptaClux: existing VAE encoder (same architecture as used in the study); trained on CS-R3 with 273-D inputs.
- AptaDiff: repository VAE (vae/scripts/real.py); trained on CS-R3.
- RaptGen: repository encoder trained on CS-R3 (standard settings).
- No diffusion/BO steps are used for any model (generation is not needed for ranking).
- Fixed random seed per run; early stopping/epoch budget kept in the same order-of-magnitude across methods, all trained files and results were accessible via <https://github.com/zibin-zhao/DL-SELEX>.

**Evaluation metrics (computed on the labeled CS panel)**

Let $S$ be the set of 8 panel sequences and $y\in\{0, 1\}$ the binder label.

- AUROC: area under ROC derived from (score, y); insensitive to monotone transforms of the score.
- AUPRC (Average Precision): area under precision–recall curve for the positive class.
- Top-k hit rate: fraction of positives among the top-k ranked sequences (k = 1, 3, 5).
- Metrics are reported per method with the same labeled panel; no panel labels are used for training or bandwidth selection.

**Selection/label logic**

- Positives (n=2): ITC-validated CS binders.
- Negatives (n=6): TES sequences confirmed by ITC to have low affinity (specificity) to CS.
- Labels are mapped to panel_id and merged with the per-sequence scores for metric computation.
- All CS aptamer panel’s aptamers are summarized in Table S9.

## Note S9 | Experimental Verification of Predicted Contacts via ITC Notes


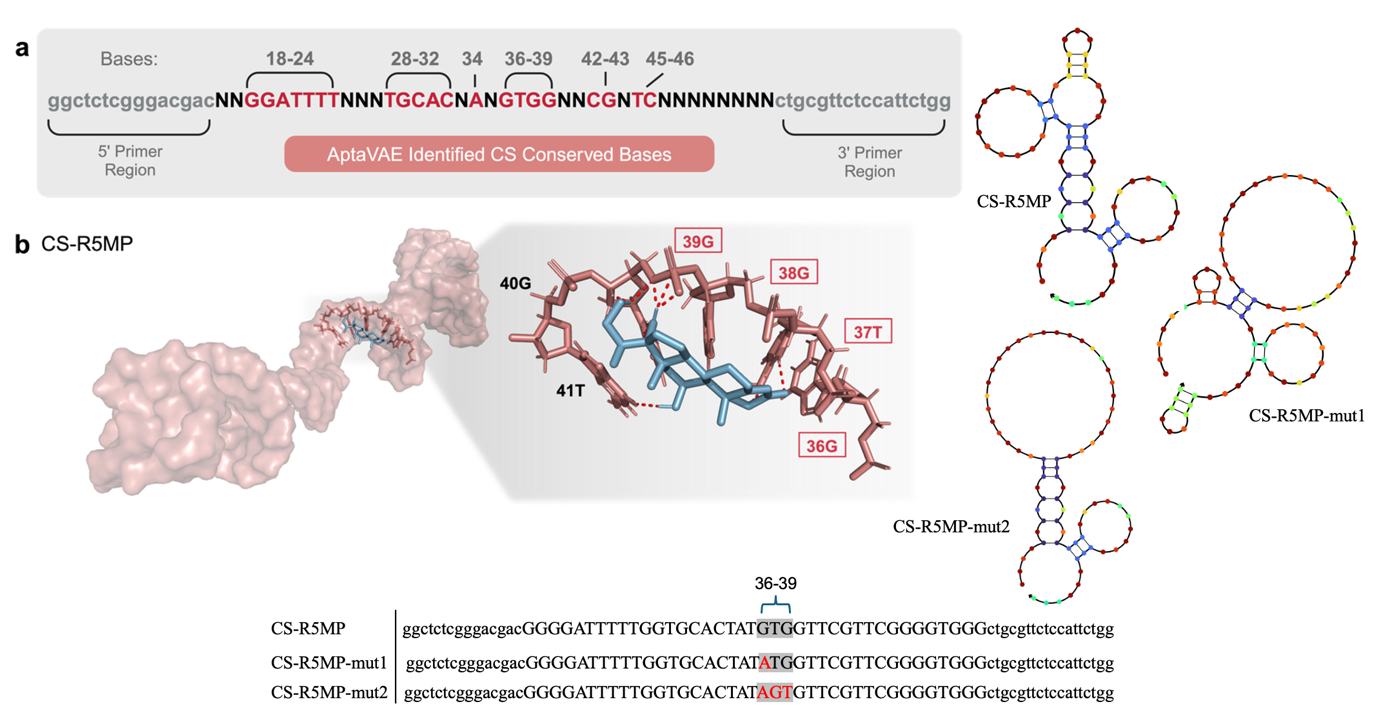


**CS target aptamers case study.** To test whether the MD/docking-predicted contact bases are functionally important, we introduced **point mutations** at the MD-identified sites. **Mut-1** perturbs a single contact to weaken binding with minimal fold disruption, whereas **Mut-2** was designed to disrupt the local 36–39 motif and thereby abolish binding. Consistent with these predictions, ITC (Fig. S71) shows a large **loss of affinity** relative to wild-type CS-R5MP (K_D_ **≈ 2.41 µM): Mut-1** exhibits K_D_ **≈ 3.95 mM,** and **Mut-2** K_D_ ≈ **192 mM**. The stepwise deterioration from Mut-1 to Mut-2 supports the MD/docking assignment of the 36–39 contacts as key contributors to ligand recognition, secondary structures were predicted with NUPACK (nupack.org).

**
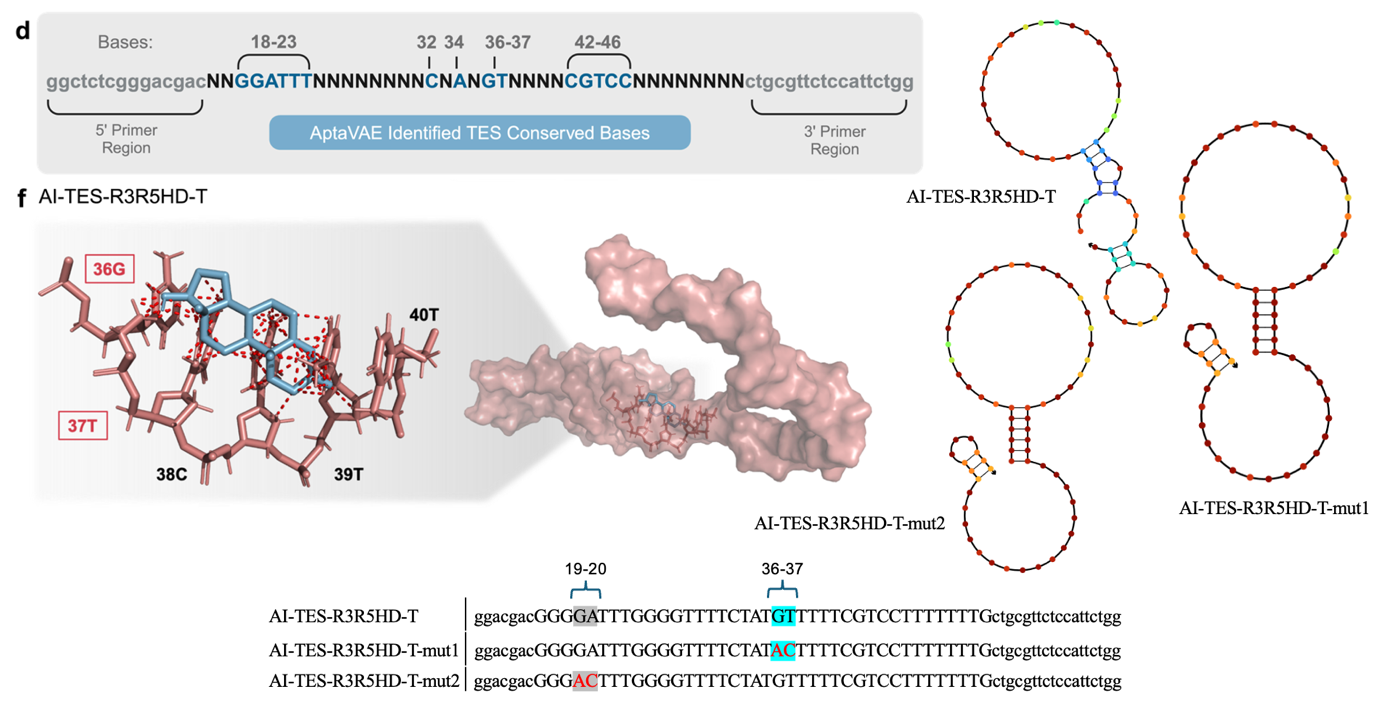
**

**TES case study.** Mut-1 was designed to disrupt the first stem–loop centered at positions 36–37, and Mut-2 to disrupt the second stem–loop at 19–20 (numbering refers to the full-length aptamer). The wild-type AI-TES-R3R5HD-T binds with K_D_ ≈ 0.323 µM, whereas Mut-1 and Mut-2 showed no measurable binding by ITC (oscillating, non-saturating isotherms), consistent with loss of the predicted secondary-structure elements required for affinity, sequence secondary structures were predicted with NUPACK (nupack.org).

## Note S10 | Modified Attention Mechanism Formalization

For single head,

Let $Q=XW_{Q}, K=YW_{K}, V=YW_{V}$, with head size $d_{h}$.
We modify scaled dot-product attention by adding a **query passthrough** term:

$$\hat{Z}=softmax(\frac{QK^{T}}{\sqrt{d_{h}}})V+\lambda\frac{Q}{\sqrt{d_{h}}}$$

- When ($\lambda=0$) this reduces to the standard attention.
- The factor ($\frac{1}{\sqrt{d_{h}}}$) keeps both summands on the same scale as in standard attention.

For multi-head, apply per head and concatenate as usual.

**References**

1. Yang LF, Ling M, Kacherovsky N, et al. Aptamers 101: aptamer discovery and *in vitro* applications in biosensors and separations. Chem. Sci. 2023; 14:4961–4978

2. Mahmoudian F, Ahmari A, Shabani S, et al. Aptamers as an approach to targeted cancer therapy. Cancer Cell Int 2024; 24:108

3. Wandtke T, Wędrowska E, Szczur M, et al. Aptamers—Diagnostic and Therapeutic Solution in SARS-CoV-2. IJMS 2022; 23:1412

4. Yoo H, Jo H, Oh SS. Detection and beyond: challenges and advances in aptamer-based biosensors. Mater. Adv. 2020; 1:2663–2687

5. DeRosa MC, Lin A, Mallikaratchy P, et al. In vitro selection of aptamers and their applications. Nat Rev Methods Primers 2023; 3:54

6. Brown A, Brill J, Amini R, et al. Development of Better Aptamers: Structured Library Approaches, Selection Methods, and Chemical Modifications. Angew Chem Int Ed 2024; 63:e202318665

7. Wang L, Alkhamis O, Canoura J, et al. Rapid Nuclease-Assisted Selection of High-Affinity Small-Molecule Aptamers. J. Am. Chem. Soc. 2024; 146:21296–21307

8. Singh NK, Wang Y, Wen C, et al. High-affinity one-step aptamer selection using a non-fouling porous hydrogel. Nat Biotechnol 2024; 42:1224–1231

9. Yu H, Canoura J, Byrd C, et al. Improving aptamer affinity and determining sequence–activity relationships via motif-SELEX. J. Am. Chem. Soc. 2025; 147:9472–9486

10. Yu H, Alkhamis O, Canoura J, et al. Advances and Challenges in Small‐Molecule DNA Aptamer Isolation, Characterization, and Sensor Development. Angew. Chem. Int. Ed. 2021; 60:16800–16823

11. Komarova N, Kuznetsov A. Inside the Black Box: What Makes SELEX Better? Molecules 2019; 24:E3598

12. Kohlberger M, Gadermaier G. SELEX: Critical factors and optimization strategies for successful aptamer selection. Biotech and App Biochem 2022; 69:1771–1792

13. Bashir A, Yang Q, Wang J, et al. Machine learning guided aptamer refinement and discovery. Nat Commun 2021; 12:2366

14. Chen JC, Chen JP, Shen MW, et al. Generating experimentally unrelated target molecule-binding highly functionalized nucleic-acid polymers using machine learning. Nat Commun 2022; 13:4541

15. Iwano N, Adachi T, Aoki K, et al. Generative aptamer discovery using RaptGen. Nat Comput Sci 2022; 2:378–386

16. Di Gioacchino A, Procyk J, Molari M, et al. Generative and interpretable machine learning for aptamer design and analysis of in vitro sequence selection. PLoS Comput Biol 2022; 18:e1010561

17. Rube HT, Rastogi C, Feng S, et al. Prediction of protein–ligand binding affinity from sequencing data with interpretable machine learning. Nat Biotechnol 2022; 40:1520–1527

18. Alipanahi B, Delong A, Weirauch MT, et al. Predicting the sequence specificities of DNA- and RNA-binding proteins by deep learning. Nat Biotechnol 2015; 33:831–838

19. Wang Z, Liu Z, Zhang W, et al. AptaDiff: de novo design and optimization of aptamers based on diffusion models. Briefings in Bioinformatics 2024; 25:bbae517

20. Emami N, Ferdousi R. AptaNet as a deep learning approach for aptamer–protein interaction prediction. Sci Rep 2021; 11:6074

21. Shin I, Kang K, Kim J, et al. AptaTrans: a deep neural network for predicting aptamer-protein interaction using pretrained encoders. BMC Bioinformatics 2023; 24:447

22. Lee EC, Fragala MS, Kavouras SA, et al. Biomarkers in Sports and Exercise: Tracking Health, Performance, and Recovery in Athletes. Journal of Strength and Conditioning Research 2017; 31:2920–2937

23. Yang K-A, Chun H, Zhang Y, et al. High-Affinity Nucleic-Acid-Based Receptors for Steroids. ACS Chem. Biol. 2017; 12:3103–3112

24. Yang K, Mitchell NM, Banerjee S, et al. A functional group–guided approach to aptamers for small molecules. Science 2023; 380:942–948

25. Vorobyeva M, Davydova A, Vorobjev P, et al. Key Aspects of Nucleic Acid Library Design for in Vitro Selection. IJMS 2018; 19:470

26. Zhu C, Feng Z, Qin H, et al. Recent progress of SELEX methods for screening nucleic acid aptamers. Talanta 2024; 266:124998

27. Yuan C, Wei A. WO2020069318A1 - Cortisol binding aptamer - Google Patents. 2018;

28. Palma M, Xu X, Clement P. WO2019102217A1 - Biosensor device and assembly methods - Google Patents. 2017

29. Martin JA, Chávez JL, Chushak Y, et al. Tunable stringency aptamer selection and gold nanoparticle assay for detection of cortisol. Anal Bioanal Chem 2014; 406:4637–4647

30. Wang B, Zhao C, Wang Z, et al. Wearable aptamer-field-effect transistor sensing system for noninvasive cortisol monitoring. Science Advances 2022; 8:eabk0967

31. Niu C, Ding Y, Zhang C, et al. Comparing two cortisol aptamers for label-free fluorescent and colorimetric biosensors. Sens. Diagn. 2022; 1:541–549

32. Jauset-Rubio M, Botero ML, Skouridou V, et al. One-Pot SELEX: Identification of Specific Aptamers against Diverse Steroid Targets in One Selection. ACS Omega 2019; 4:20188–20196

33. Yang K-A, Pei R, Stefanovic D, et al. Optimizing Cross-reactivity with Evolutionary Search for Sensors. J. Am. Chem. Soc. 2012; 134:1642–1647

34. Dalirirad S, Han D, Steckl AJ. Aptamer-Based Lateral Flow Biosensor for Rapid Detection of Salivary Cortisol. ACS Omega 2020; 5:32890–32898

35. Ji Y, Zhou Z, Liu H, et al. DNABERT: pre-trained Bidirectional Encoder Representations from Transformers model for DNA-language in genome. Bioinformatics 2021; 37:2112–2120

36. Chithrananda S, Grand G, Ramsundar B. ChemBERTa: Large-Scale Self-Supervised Pretraining for Molecular Property Prediction. 2020

37. Hoinka J, Backofen R, Przytycka TM. AptaSUITE: A Full-Featured Bioinformatics Framework for the Comprehensive Analysis of Aptamers from HT-SELEX Experiments. Molecular Therapy - Nucleic Acids 2018; 11:515–517

38. Wang W, Feng C, Han R, et al. trRosettaRNA: automated prediction of RNA 3D structure with transformer network. Nat Commun 2023; 14:7266

39. Abraham MJ, Murtola T, Schulz R, et al. GROMACS: High performance molecular simulations through multi-level parallelism from laptops to supercomputers. SoftwareX 2015; 1–2:19–25

40. Eberhardt J, Santos-Martins D, Tillack AF, et al. AutoDock Vina 1.2.0: New Docking Methods, Expanded Force Field, and Python Bindings. J. Chem. Inf. Model. 2021; 61:3891–3898

41. Trott O, Olson AJ. AutoDock Vina: Improving the speed and accuracy of docking with a new scoring function, efficient optimization, and multithreading. J Comput Chem 2010; 31:455–461

42. Hu G, Zhou H-X. Magnesium ions mediate ligand binding and conformational transition of the SAM/SAH riboswitch. Commun Biol 2023; 6:791

43. Plach M, Schubert T. Biophysical Characterization of Aptamer-Target Interactions. Aptamers in Biotechnology 2019; 174:1–15

44. Sun J, Philpott M, Loi D, et al. Correcting PCR amplification errors in unique molecular identifiers to generate accurate numbers of sequencing molecules. Nat Methods 2024; 21:401–405

45. Lin H, Zhao Z, Feng X, et al. DNA hydrogel-interfaced organic electrochemical transistor for the investigation of binding-induced conformational change of small molecule aptamers. ACS Appl. Mater. Interfaces 2025; 17:51723–51736

46. Chen Z, Hu L, Zhang B-T, et al. Artificial intelligence in aptamer–target binding prediction. International Journal of Molecular Sciences 2021; 22:3605

47. Rodríguez Serrano AF, Hsing I-M. Prediction of Aptamer–Small-Molecule Interactions Using Metastable States from Multiple Independent Molecular Dynamics Simulations. J. Chem. Inf. Model. 2022; acs.jcim.2c00734

48. van Gunsteren WF, Daura X, Hansen N, et al. Validation of Molecular Simulation: An Overview of Issues. Angew Chem Int Ed 2018; 57:884–902

49. Zulkeflee Sabri M, Azzar Abdul Hamid A, Mariam Sayed Hitam S, et al. In-silico selection of aptamer: A review on the revolutionary approach to understand the aptamer design and interaction through computational chemistry. Materials Today: Proceedings 2019; 19:1572–1581
