# Supplementary figures and images for: Structure-enhanced deep learning accelerates aptamer selection for small molecule families like steroids
Source: Brief Bioinform. 2025 Dec 18;26(6):bbaf680. doi: 10.1093/bib/bbaf680 (PMC12713628; doi:10.1093/bib/bbaf680)

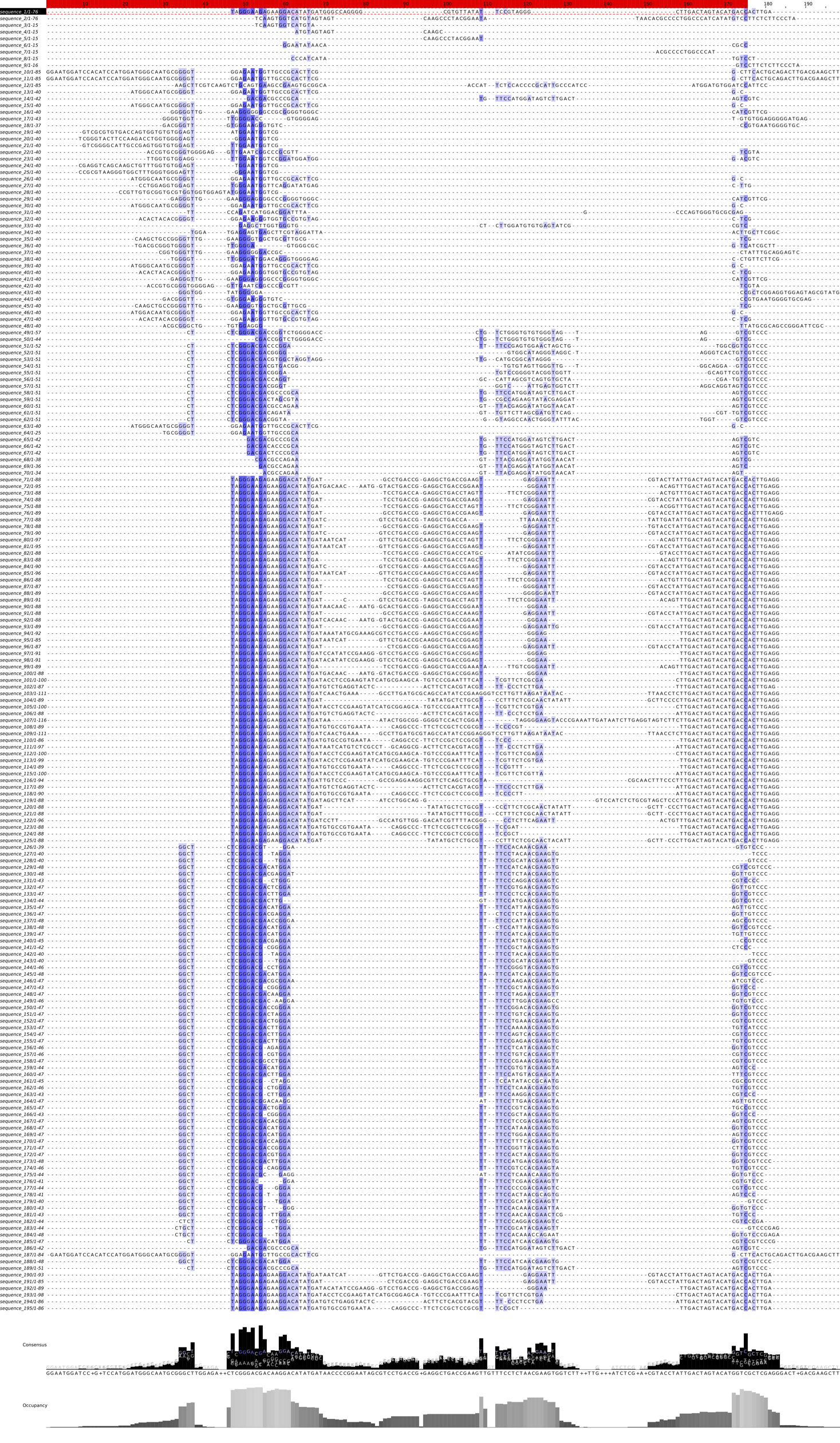

Supplement: SI-Extended-Figure-S19_bbaf680 [file si-extended-figure-s19_bbaf680.jpeg]
